# Microwave-assisted synthesis of new triangulenium dyes for lifetime imaging microscopy (FLIM)

**DOI:** 10.1038/s41598-025-31688-6

**Published:** 2025-12-21

**Authors:** Carmen Viedma-Barba, Esther M. Ortega-Naranjo, Federico Movilla, Antonio Reinoso, Marta Andrades-Amate, Pablo Peñalver, Juan C. Morales, Juan A. González-Vera, Marta Gutiérrez-Rodríguez, Angel Orte

**Affiliations:** 1 Instituto de Química Médica, IQM-CSIC, 28006 Madrid, Spain; 2 Nanoscopy-UGR Lab, Dept. Fisicoquímica, Facultad de Farmacia, Universidad de Granada, Granada, 18071 Spain; 3 Departamento de Bioquímica y Biología Molecular II, Facultad de Farmacia, Universidad de Granada, Granada, 18071 Spain; 4 Instituto de Parasitología y Biomedicina López-Neyra, CSIC, PTS Granada, Avda. del Conocimiento, 17, Armilla, 18016 Spain

**Keywords:** Microwave synthesis, Dyes, Cell imaging, Advanced microscopy, Solvatochromism, Photophysics, Biological techniques, Biophysics, Chemistry, Materials science, Optics and photonics

## Abstract

We introduce a novel microwave-assisted method for the rapid and efficient synthesis of triangulenium dyes, enabling the creation of a broader range of these fluorophores, which are characterized by very long fluorescence lifetimes. We prepared different azadioxatriangulenium (ADOTA) and diazaoxatriangulenium (DAOTA) derivatives and thoroughly characterized their photophysical properties. We investigated their solvatochromic properties and the effects of the solvent, aided by computational chemistry results. The new ADOTA and DAOTA dyes were applied in multiple *in cellulo* microscopy modalities, including confocal, fluorescence lifetime imaging microscopy (FLIM), and super-resolution imaging. ADOTA derivatives predominantly accumulated in mitochondria, while DAOTAs showed pronounced nuclear staining and participation in endocytic pathways. Notably, these dyes enable clear discrimination of subcellular compartments via lifetime contrast in FLIM: mitochondria, where ADOTAs undergo electron transfer-driven quenching and display short lifetimes; nuclei and DNA, where lifetimes remain long; and endocytic vesicles, where partial dye aggregation leads to lifetime reduction.

**Supplementary Information:**

The online version contains supplementary material available at 10.1038/s41598-025-31688-6.

## Introduction

Triangulenium dyes are highly stable, planarized triarylmethyl cations. The triangulenium core possesses a carbocationic charge delocalized over an extended aromatic system, giving rise to strong fluorescence emission. Triangulenium dyes can be synthetically modified, through a series of modular pathways, to adjust their properties and for the evaluation of structure-function relationships^1–4^. These dyes offer a wide range of attractive structural, physicochemical, and spectral properties^5^. From the initial synthesis of stable derivatives of the trioxatriangulenium ion (TOTA),^6^ the introduction of aza bridges in the core led to highly efficient azadioxatriangulenium (ADOTA) and diazaoxatriangulenium dyes (DAOTA)^7^. One of the most important photophysical features of this kind of dyes is the very long fluorescence lifetime, τ_f_, of around 20 ns.

Such long-lived fluorescent triangulenium dyes provide an excellent platform for time-resolved sensing and bioimaging^8,9^. These dyes have been successfully employed in fluorescence polarization assays,^10,11^ as pH sensors,^12^ G-quadruplex DNA binders,^16^ and even water sensing^19^. The sensing mechanism is usually driven by photoinduced electron transfer (PET) quenching, which can be modulated by the different substituents within the triangulenium moiety^2,14,20^. ADOTA and DAOTA dyes present a rich redox behavior, with aza bridges increasing the reduction potential and leading to a lower electron-accepting capacity^2,20^. Thus, the design of new triangulenium dyes involves the careful control of electron donating or withdrawing groups, or the addition of different functional groups, such as bulkier alkyl or aryl groups. Notably, side groups can also modulate intramolecular charge transfer (ICT) processes, altering the photophysical properties of the dyes. ICT, PET quenching, and thus potential sensing mechanisms can also be altered by general and specific solvent effects. In fact, the efficiency of the PET quenching can be modified by hydrogen bonding with the solvent via proton coupled electron transfer (PCET)^19^. Although some triangulenium dyes have been reported to dimerize and form ion pairs in non-polar solvents,^21^ to date no profound investigation of the general and specific solvent effects on triangulenium fluorescence properties has been performed.

Triangulenium dyes are synthesized from readily available precursors through sequences of highly selective nucleophilic aromatic substitution reactions and subsequent intramolecular ring closure. Nevertheless, the synthesis of these dyes is often hindered by long reaction times and modest yields, which can limit their broader applicability and scalability^3,4,7,8,11,22–24^. These challenges underscore the need for improved synthetic methodologies to facilitate access to diverse triangulenium derivatives for advanced bioimaging and biolabeling applications.

**Fig. 1:**
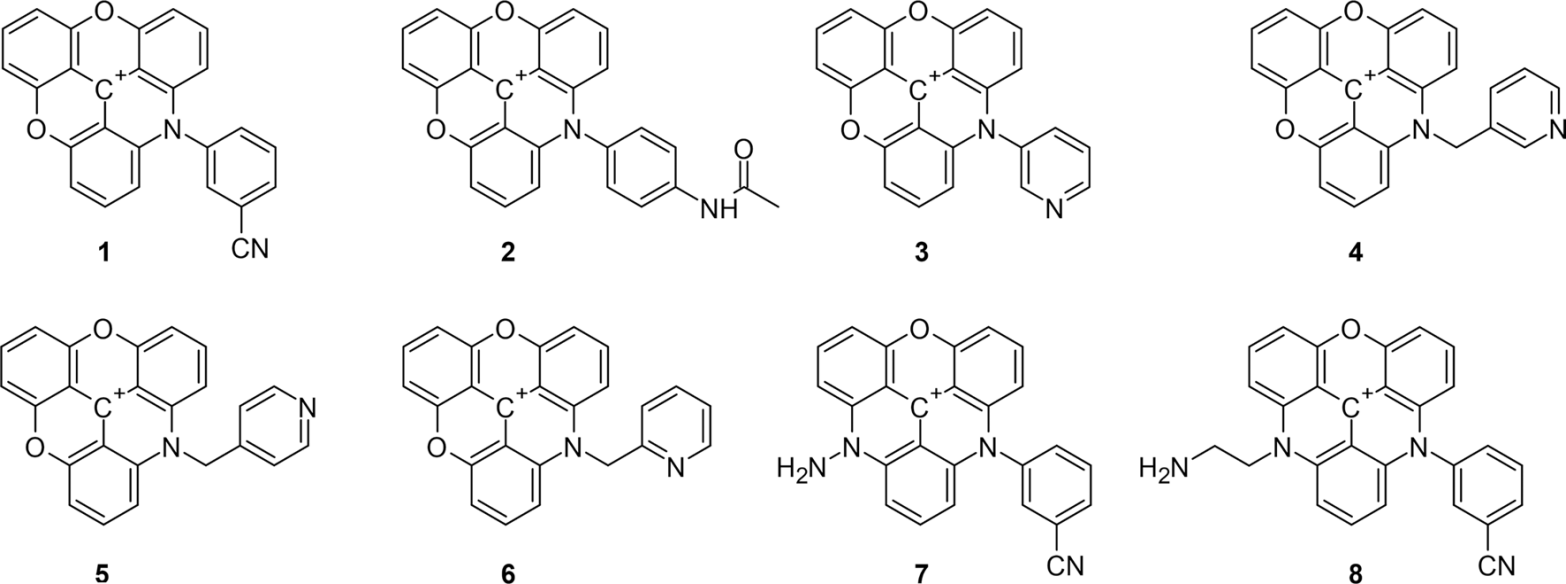
Structures of the ADOTAs **1**–**6** and DAOTAs **7** and **8** synthesized in this work.

Herein, we present for the first time a microwave-assisted method for the synthesis of novel ADOTA and DAOTA dyes (Fig. 1) that provides a fast and efficient route to a variety of substituted triangulenium dyes. This work broadens the palette of available fluorophores with long τ_f_ values and provides a thorough investigation of their solvatochromic behavior through different formalisms, such as that proposed by Catalán,^25^ with special attention to unravel the solvent features that further modulate and influence the luminescence properties of the dyes. Importantly, their very long τ_f_ values make these dyes especially useful in fluorescence lifetime imaging microscopy (FLIM), as they expand the sensitivity range of lifetime contrast and fostering accurate time-gated filtering analysis.

## Results and discussion

### Synthesis

Although microwave-assisted synthesis has been employed to obtain various types of dyes,^26^ its application to the synthesis of triangulenium dyes remains unexplored. Starting from the reported triarylcarbenium precursor **9**, the novel process involves two nucleophilic aromatic substitution reactions (S_N_Ar), both performed under microwave conditions, and a ring-closing step^26^. To investigate the potential advantages of this approach, we first conducted a comparative analysis of the conventional heating method *versus* the microwave-assisted method for the synthesis of novel ADOTA (**1**) and DAOTA (**7**) dyes, bearing a 3-benzonitrile.

It is well established that incorporating anilines—whose nucleophilicity is lower than that of aliphatic amines—into the triangulenium core requires more demanding reaction conditions, including longer reaction times and higher temperatures^18^. The reaction of precursor **9** with 3-aminobenzonitrile was carried out at 80 °C (Table 1).

**Table 1:**
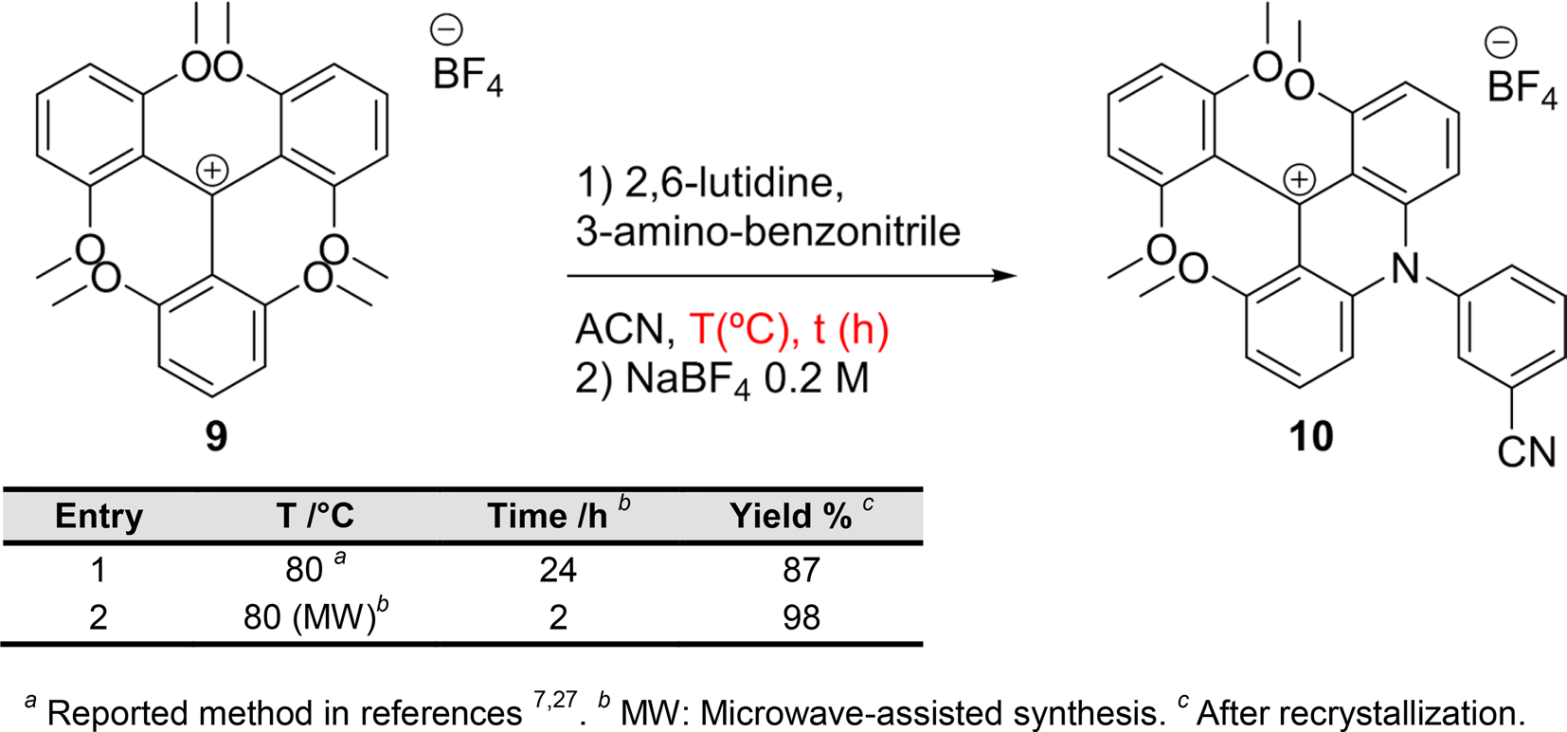
Comparative study of the conventional heating method *versus* the microwave-assisted method for the synthesis of **10** as precursor of novel ADOTA and DAOTA dyes.

^*a*^ Reported method in references^7,27^. ^*b*^ MW: Microwave-assisted synthesis. ^*c*^ After recrystallization.

**Fig. 2:**
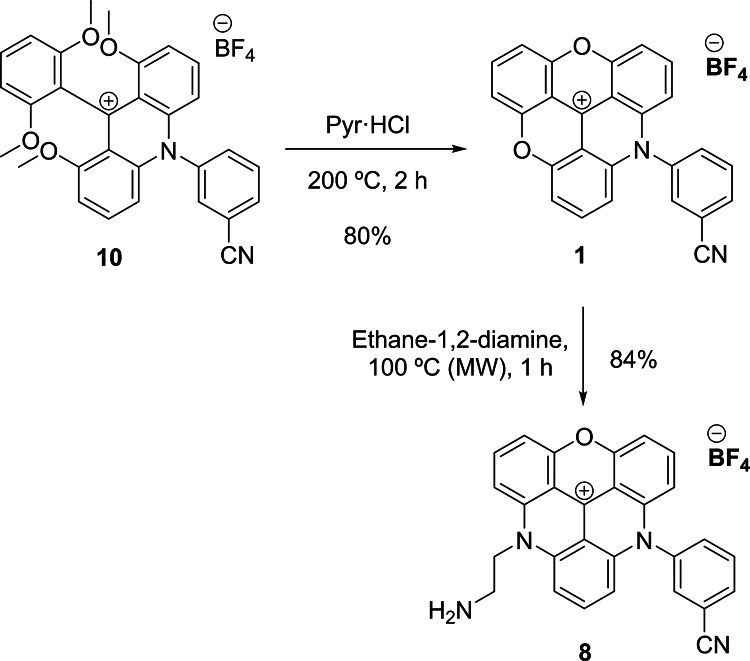
Synthesis of novel ADOTA **1** and DAOTA **8**.

Using conventional heating, the reaction took place in 24 h and the novel acridinium salt **10** was obtained with an 87% yield (Table 1, entry 1). However, under microwave conditions (Table 1, entry 2), the reaction proceeds 10 times faster (2 h) and affords the acridinium salt **10** in nearly quantitative yields and 99% purity, highlighting the benefits of the microwave-assisted synthesis including significantly shorter reaction times and higher efficiency. Next, the ring-closing reaction of the acridinium salt **10** was carried out in molten pyridinium chloride^28^ to obtain the novel ADOTA dye **1** with an 80% yield (Fig. 2). The second S_N_Ar reaction was also explored for the first time using a microwave-assisted strategy, by reaction of ADOTA **1** with ethane-1,2-diamine (Fig. 2). Under microwave-assisted conditions at 100 °C, the novel DAOTA dye **8** was obtained within just 1 h with an excellent yield (84%) and 99% purity after recrystallization.

Encouraged for the excellent results in terms of speed and efficiency, we extended the novel microwave-assisted synthesis approach to the preparation of the new ADOTA **3**–**6** and DAOTA **7**. Therefore, the microwave-assisted synthesis of the acridinium salts **11**–**15** was carried out at 80 °C. The reaction times ranged from 1 min to 2 h, depending on the electronic and steric properties of the substituents attached to the primary amines, and in all the cases, excellent yields and purities were obtained (Table 2).

**Table 2:**
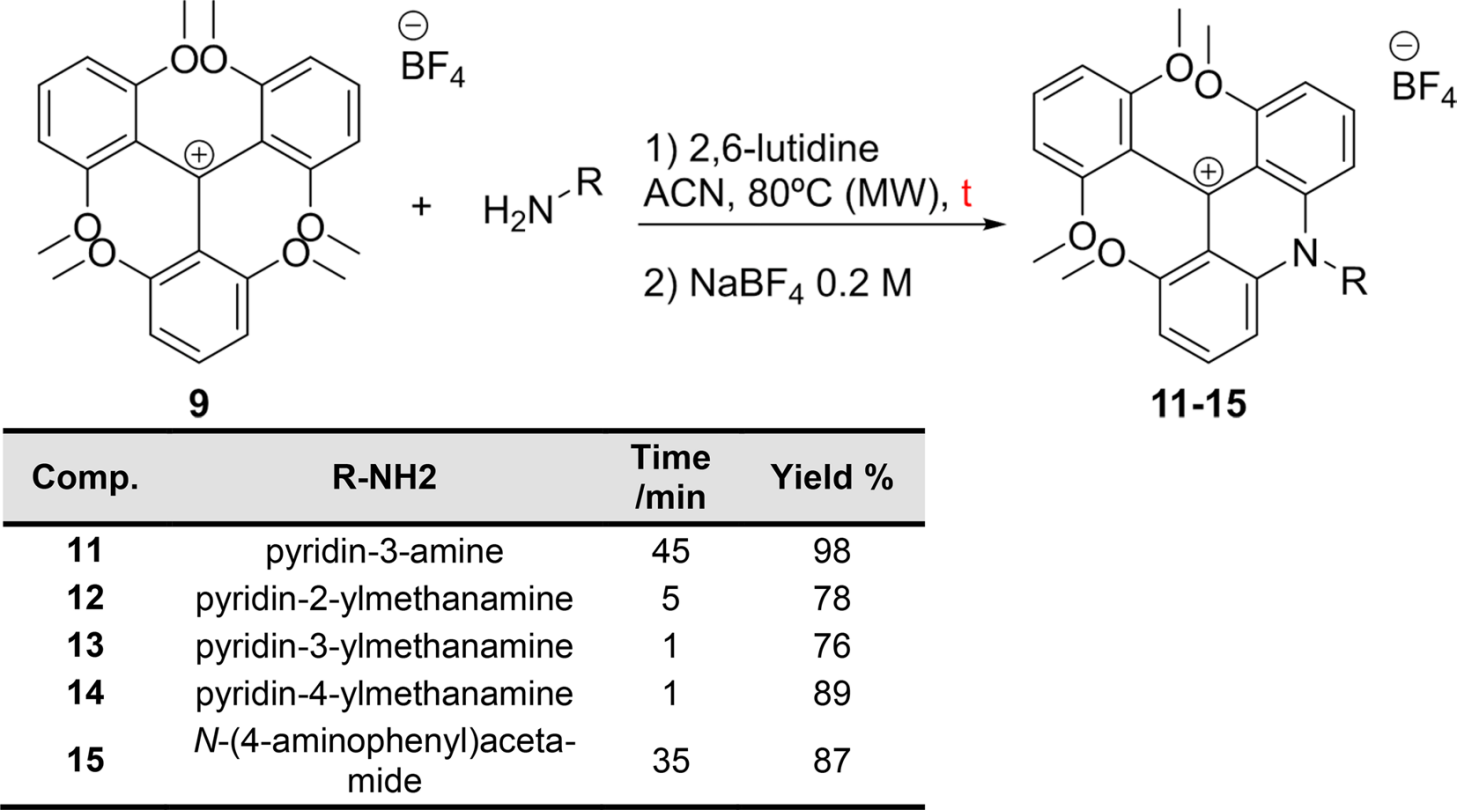
Microwave-assisted method for the synthesis of **11**–**15** as precursors of novel ADOTA and DAOTA dyes.

**Fig. 3:**
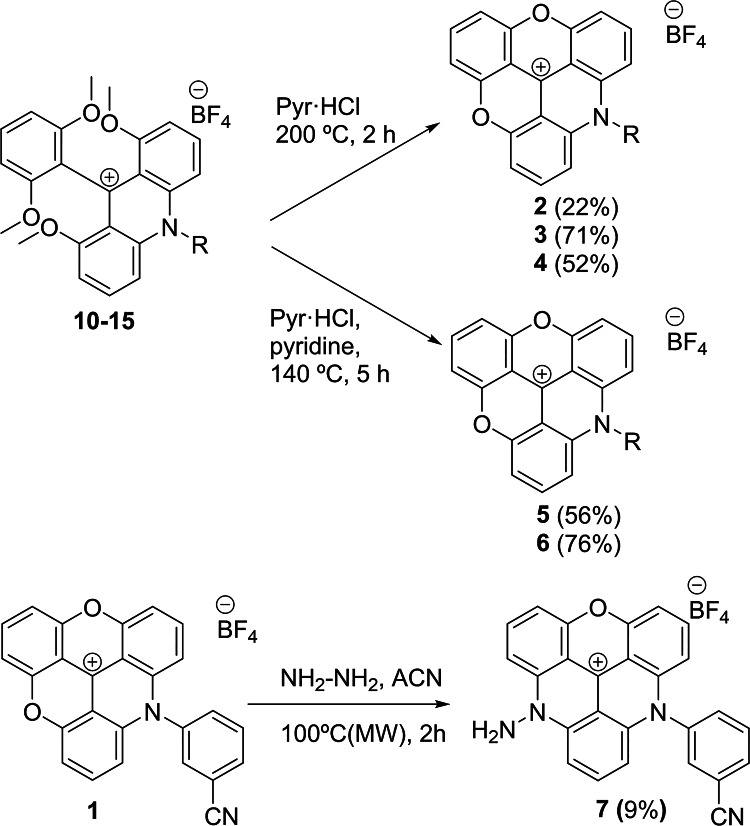
Synthesis of ADOTAs **2**–**6** and DAOTA **7**.

Next, the ring-closing reaction afforded the ADOTA dyes **3–6** with good to excellent yields and 99% purities (Fig. 3 and Supporting Information, SI). The DAOTA dye **7** was obtained from the corresponding ADOTA **1** by a second S_N_Ar reaction with hydrazine at 100 °C under microwave heating in 2 h, with a 9% yield and 99% purity (Fig. 3).

Compared to the reported reaction times and yields for triangulenium dyes with similarly nucleophilic substituents prepared by conventional heating or room temperature,^7,27^ the novel microwave-assisted methodology here described offers improved speed and efficiency, marking a clear step forward in the synthesis of this dye family.

### Photophysical properties

**Fig. 4:**
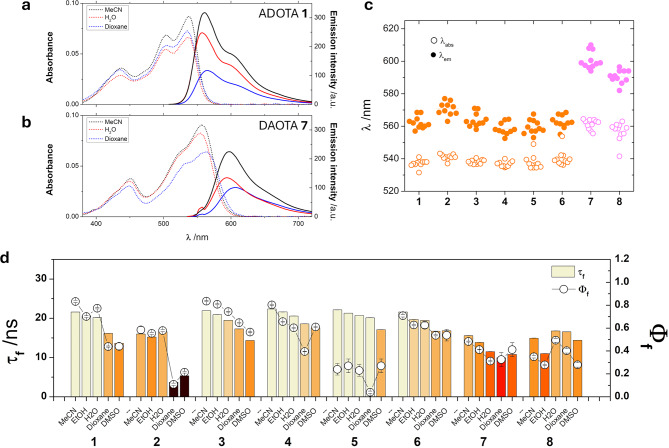
Photophysical characterization of ADOTAs **1**–**6** and DAOTAs **7** and **8**. **a**,** b**) Representative absorbance (dotted lines) and emission spectra (solid lines) of (**a**) ADOTA **1** (λ_ex_ = 505 nm) and (**b**) DAOTA **7** (λ_ex_ = 555 nm) at 10 µM in MeCN (black), H_2_O (red), or dioxane (blue). c) Absorbance (open symbols) and emission maxima (closed symbols) of ADOTAs **1**–**6** (orange) and DAOTAs **7**–**8** (magenta) in all the studied solvents. **d**) Intensity-weighted average fluorescence lifetime (τ_f_, bars) and quantum yield values (Φ_f_, symbols) of ADOTAs **1**–**6** and DAOTAs **7**–**8** in selected solvents. Error bars represent standard deviations (*n* = 3, for τ_f_; *n* = 8, for Φ_f_).

The photophysical properties of synthesized triangulenium dyes were investigated to assess the effect of the different side groups and their response to the microenvironment, using a range of twelve solvents of different polarity and acid-base properties (see Tables S1-S8 in the SI). All the studied dyes exhibited comparable excitation and emission wavelengths, with absorption maximum values ranging from 531.5 to 553.8 nm for ADOTAs and from 541.5 to 564.5 nm for DAOTAs. These wavelengths were assigned to the S_0_→S_1_ transition, in which a vibronic structure is visible. The S_0_→S_2_ transition was detected in the 430 nm region. See Fig. 4a-b for representative spectra. The emission maximum values ranged from 552.5 to 577 nm for ADOTAs and from 582 to 610 nm for DAOTAs (Fig. 4c), with larger Stokes shift values for the latter. These values agree with those reported for previously published triangulenium dyes, supporting that the main triangulenium core serves as the emissive moiety and that the effect of side groups is small. Fluorescence quantum yields (Φ_f_) were typically between 0.2 and 0.7, with lifetimes (τ_f_) in the 15–20 ns range (Fig. 4d and Tables S1-S8). A strong correlation between τ_f_ and Φ_f_ indicates that nonradiative deactivation is the main modulator of both parameters. Interestingly, *k*_*nr*_ rates exhibited dependence with the solvent, increasing its value in more apolar solvents (Figure S1).

An in-depth analysis of the fluorescence decay times provides valuable insights into the behavior of the dyes in various solvents (see Supporting Information for datasheets). ADOTAs **3** and **4** displayed predominantly monoexponential decay traces in all solvents except 1,4-dioxane, whereas the remaining ADOTAs exhibited biexponential decays in long-chain alcohols and in dioxane. In most of these solvents, the weight of a short decay time was below 20% (below 10% considering the intensity of emission), thus constituting a low contribution. In many cases, the decay traces recorded in toluene showed a pronounced contribution from scattered light, indicative of the presence of colloidal particles. In fact, ADOTAs **2**, **3** and **4** were not soluble in toluene. DAOTAs **7** and **8**, in contrast, consistently exhibited multiexponential decay behavior. These findings support the hypothesis that decreasing solvent dipolarity promotes dye aggregation. Although the triangulenium moiety carries a positive charge that should lead to electrostatic repulsion and thus hinder aggregation, the formation of ion pairs in triangulenium dyes has been reported in highly apolar solvents such as benzene or methylcyclohexane,^21,29^ whereas the dyes remain molecularly dispersed in solvents like dichloromethane. However, we did not detect definitive evidence of ion pairing-driven band splitting in the absorption spectra at the working concentrations.

Quenching through PET is likely behind the lower τ_f_ and Φ_f_ values observed in DAOTAs modified with amines, **7** and **8**, and the acetylated ADOTA **2**. A related compound, carrying a *para*-primary amine in the phenyl group (N-*p*-aminophenyl-ADOTA), was previously reported to be highly quenched in MeCN due to strong PET from the primary amine to the triangulenium moiety^2^. Acetylation of the amine in dye **2** mostly prevents the PET to occur, partially recovering the characteristic luminescent properties of triangulenium dyes. PET is particularly hindered in solvents with hydrogen donating properties, like water and alcohols, but the lifetime and quantum yield of **2** is lower in solvents like DMSO and dioxane, where the electrons of the amide –NH– interact less with the solvent, causing PET upon excitation. Similarly, PET-based quenching by amines is not as effective in DAOTAs due to their lower electron accepting ability and higher (negative) reduction potential compared to ADOTAs, due to the extra electrons in the core^14,20^. Moreover, the modulation of PET quenching in water is likely behind the biexponential decay traces in ADOTA **2** and DAOTAs **7** and **8**.

To get more insights into the solvent effects over the photophysical properties of the dyes, we decided to perform an in-depth study of the solvatochromic behavior of the dyes, following the formalism established by J. Catalán, who set four solvent scales for general solvent effects (solvent dipolarity, SdP, and solvent polarizability, SP) and specific solvent effects (solvent acidity, SA, and solvent basicity, SB)^25^. This study (see SI for details and Figure S2) revealed that solvent polarizability is the dominant factor influencing the excited state stability of most dyes, leading to red shifts in both absorption and emission. The fact that the triangulene moiety is charged and the very long photoluminescence lifetime values are the main reasons behind the solvatochromic dependency on solvent polarizability. Solvent acidity was also found to regulate emission, causing blue shifts. This is caused by specific solvent acidic interactions with the –O– and –N– basic bridges in the triangulenium moiety, reducing the donation of electrons to the core and thus decreasing the stabilization of the excited state. Interestingly, the effect of solvent acidity or basicity on the photoluminescence τ_f_ values was found for dyes in which partial PET quenching occurs, namely ADOTA **2** and DAOTAs **7** and **8**. This suggests that specific proton transfer interactions can modulate the efficiency of the PET process. In fact, modulation of PET quenching by specific acid-base properties has been proposed for designing pH probes^14^ and pH-responsive materials^13,15,30^ based on the triangulenium core.

**Fig. 5:**
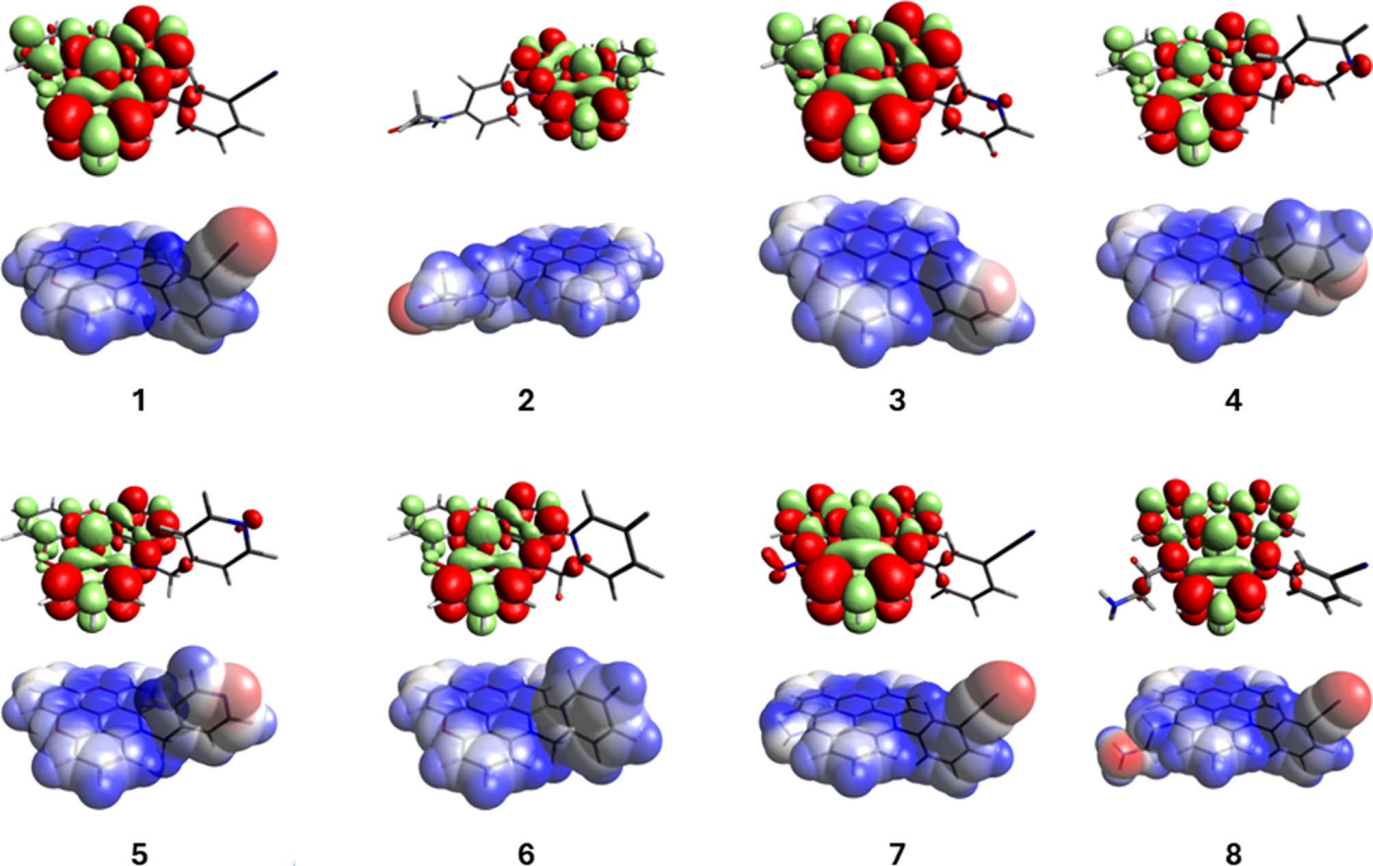
Density difference plots for the excitation electronic transitions (above) and electrostatic potential plotted over the Van der Waals surface (EPS) for dyes **1**–**8**. The difference density representation depicts the zones of electron density enhancement (green) or depletion (red) during the excitation transition, thus making it easy to understand the absorption phenomena as a simpler single-electron picture, in contrast to the most common canonical bonding orbital representation (in Figure S3 in the SI).

Finally, TD-DFT calculations were performed to optimize the ground and excited state geometries and calculate the orbital contribution to the most probable transitions to study the potential changes in electron density generated during excitation. Figure 5 shows the net changes in electron density distribution for the excitation transition and the electrostatic potential surface of the ground state (EPS) for each dye. The density difference plots show a general trend where the transition alters mostly the electron density on the triangulenium ring with a small contribution from the aromatic side groups. An exception is DAOTA **8**, which shows a small side contribution from the σ-bond between C-α and C-β of the amine, and from the hydrogen next to the nitrogen of the triangulenium ring (Fig. 5). This explains the large effect of solvent acidity on the properties of DAOTA **8**, since hydrogen bonding with the amine group largely affects the electron density on the σ-bond and will shift the electron density over the hydrogen, deshielding it and thus increasing its acidity. This deshielding effect could be clearly seen on the H^1^-NMR spectrum, where both of the methylene hydrogens on the β-position showed a chemical shift of 4.59 ppm (see NMR spectra on the SI, Annex 1), a much higher value compared to the expected for ethylenediamine (~ 2.75 ppm) or N-ethylpiperidine (~ 2.35 ppm)^31^. In contrast, for DAOTA **7** in which the amine is directly linked to the triangulenium, no contribution of the solvent acidity was observed (Figure S2).

Overall, our results show that, although not appreciable net charge separation occurs during the transition, side groups make a non-negligible contribution to the transition and modulate the photophysical properties through interactions with the surroundings. Interestingly, in all cases, the side group contribution is allocated in the same molecular fragment that presents the more electronegative atom (represented as a red region on the EPS), thus indicating a clear involvement of the electron rich molecular fragments in the overall absorption transition. It is important to note how, for DAOTA **8**, a strong blue zone could be seen around the H-β due to the lack of electron density, a fact that strongly supports the increase in acidity previously discussed.

### Cell imaging

The long fluorescence lifetimes of triangulenium dyes offer exceptional opportunities for FLIM and TG imaging^9,32^. Triangulenium derivatives have previously been employed for FLIM imaging of G-quadruplex structures^18^. Therefore, we evaluated the new ADOTA and DAOTA derivatives (**1**–**8**) for FLIM imaging in HeLa cell cultures, focusing initially on their subcellular localization. Among the various strategies available for mitochondrial targeting,^33,34^ the most widely used relies on delocalized lipophilic cations,^35,36^ which accumulate efficiently in mitochondria due to the organelle’s highly negative membrane potential. The lipophilicity of the dyes, in terms of estimated logP,^37^ ranges from 2.35 for DAOTA **8** to 3.58 for ADOTA **1**, placing them within the optimal range for effective mitochondrial accumulation (Table

 S9)^40^. Consequently, we performed a colocalization study of dyes **1**–**8** with the commercial mitochondria staining probe MitoTracker Deep Red (MT).

Prior to imaging, we assessed the cytotoxicity of the dyes to determine suitable working concentrations. The EC_50_ values for dyes **1**–**8** ranged from 3.6 to 10.2 µM after 72 h of incubation (Table S10). Imaging experiments with varying incubation concentrations established 2 µM as the optimal value, confirming rapid cellular uptake and high photostability of the dyes (Figure S4).

**Fig. 6:**
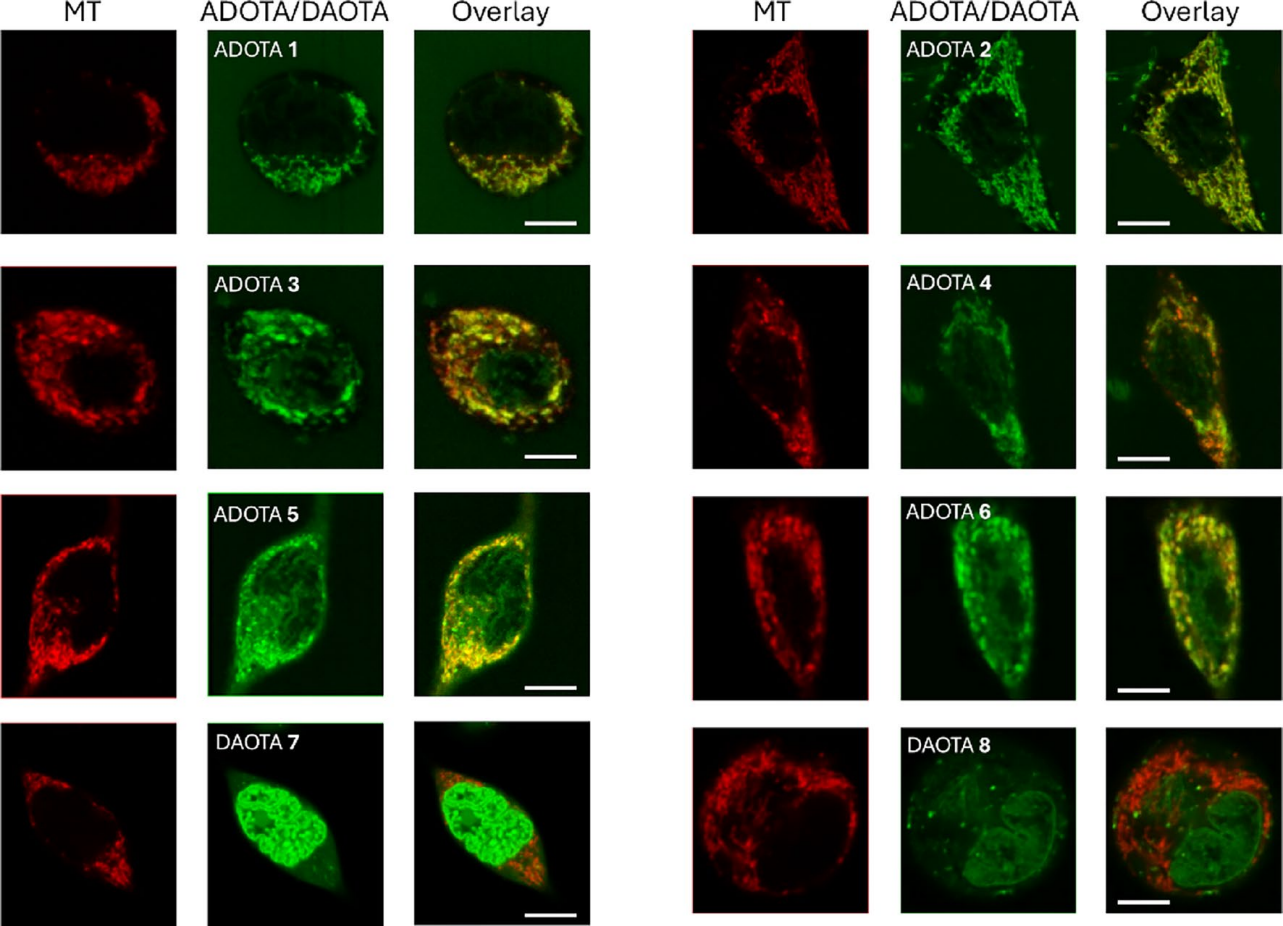
Colocalization imaging of dyes **1**–**8** (2 µM; green channel, λ_ex_ = 485 nm, detection bandpass at 605/50 nm) and MT (red channel, λ_ex_ = 640 nm, detection bandpass at 685/70 nm) in HeLa cells. Scale bars represent 10 μm.

**Fig. 7:**
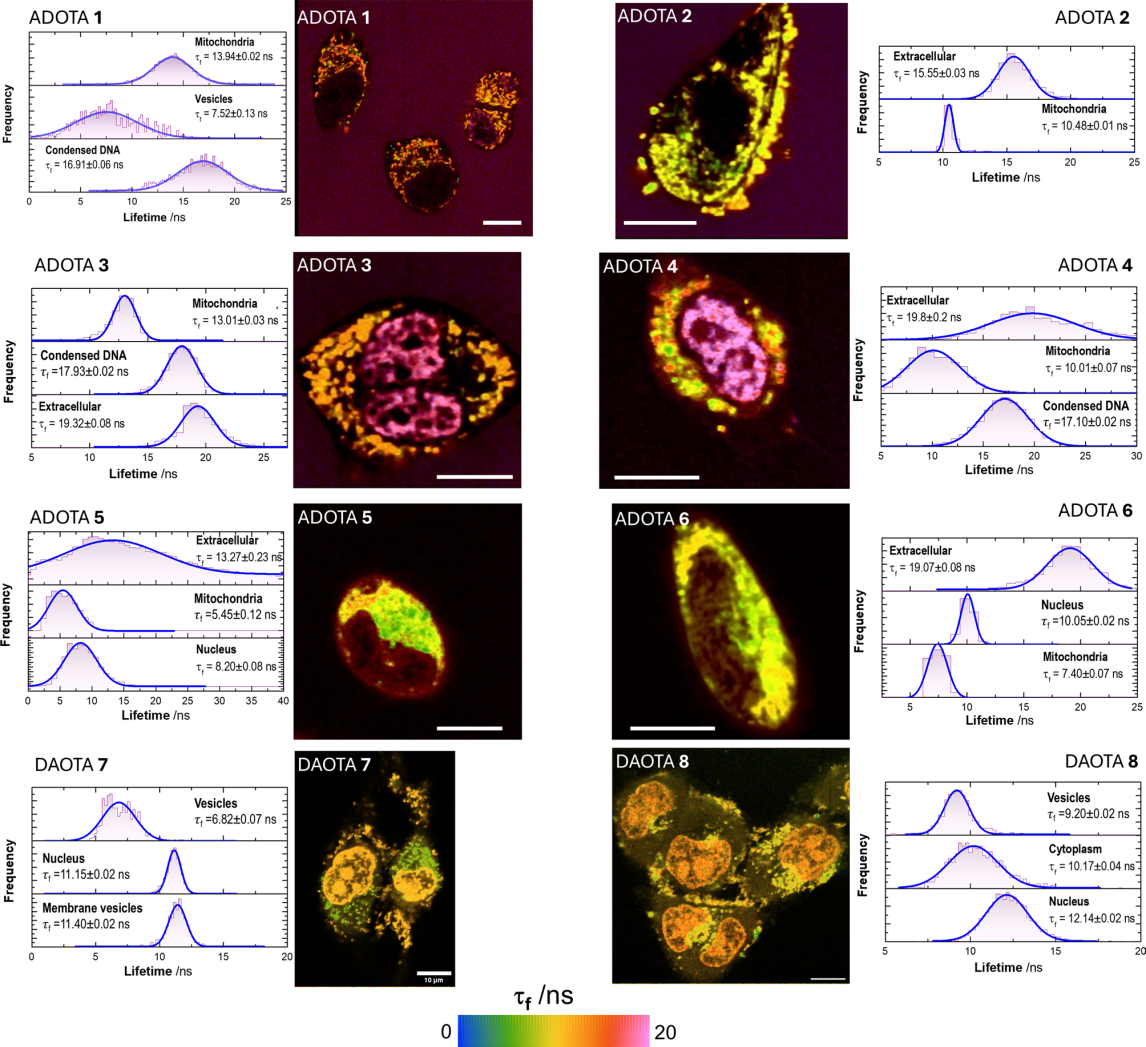
Representative FLIM images of dyes **1**–**8** (2 µM) in HeLa cells (λ_ex_ = 485 nm; detection band 580–630 nm). The pseudocolor scale represents the τ_f_ values between 0 and 20 ns. Scale bars in the images represent 10 μm. The lifetime distributions obtained from different regions of interest ROIs (cellular nuclei, condensed DNA, cell cytoplasm, membranes, mitochondria, or even the extracellular space for dyes exhibiting slower uptake) of all the collected images (*n* > 10 for each dye) are shown. The lifetime histograms were fitted to Gaussian distributions and the central position values of the distributions are reported in the image.

We then studied the colocalization of our dyes with MT using dual-color confocal microscopy. Mander’s coefficients were calculated for each channel (Table 3) and averaged over at least ten images acquired at different focal planes, providing statistically representative results. All ADOTA derivatives (**1**–**6**) displayed strong colocalization with MT (Figs. 6 and S5), supporting their expected subcellular localization in mitochondria. For most ADOTAs, except ADOTA **2**, additional fluorescence was observed in the nucleus, reducing the quantified colocalization values. ADOTA **2** exhibited the lowest colocalization with MT due to accumulation in internalized vesicles near the plasma membrane (Figure S5). In contrast, DAOTAs **7** and **8** were primarily localized in the cell nucleus, where they stained DNA and nucleoli, resulting in low colocalization with MT. Additionally, ADOTAs **1** and **5** and DAOTAs **7** and **8** were observed in small spherical vesicles consistent with endosomes and lysosomes. The dyes’ structural features –moderate basicity and logP values > 2–can also promote endosomal and lysosomal accumulation, as predicted by QSAR models^41,42^. Although the positive charge of the dyes does not favor lysosomal incorporation via lysosomotropism, ^43^ the formation of aggregates or ion pairs may facilitate uptake through endocytic pathways. As discussed above, ADOTA **2** and DAOTAs **7** and **8** were the only compounds exhibiting dual lifetime components in aqueous solution. Accordingly, the visualization of endocytic vesicles in ADOTA **2** (Figure S5) and small vesicular structures in DAOTAs **7** and **8** (Figs. 7 and S6) is consistent with the presence of aggregated dye fractions. Similar behavior has been reported previously, where a DAOTA dye bearing two morpholino groups accumulated in the nucleus and partially in mitochondria. However, although not discussed by the authors, the dye also was observed to accumulate in small vesicles that did not correlate with lysosomes^18^.

**Table 3:** Colocalization fractions (Mander’s coefficients) in the triangulenium and MT channels for dyes **1**–**8**, averaged across 10 different images at different focal planes.

Compnd.	Green channel ^a^	Red channel ^b^	DNA stain ^c^
**1**	0.63±0.12	0.71±0.11	Partial
**2**	0.43±0.20	0.43±0.19	N
**3**	0.72±0.11	0.71±0.07	Partial
**4**	0.69±0.17	0.82±0.07	Partial
**5**	0.61±0.09	0.79±0.18	Partial
**6**	0.73±0.12	0.92±0.06	Partial
**7**	0.09±0.03	0.27±0.11	Y
**8**	0.15±0.08	0.25±0.09	Y

^*a*^ Triangulenium channel, 605/50 nm. ^*b*^ MT channel, 685/70 nm. ^*c*^ Y/N/Partial indicating whether the dye also stains nuclei and DNA.

We subsequently performed FLIM imaging of dyes **1**–**8** in HeLa cells. All compounds displayed complex lifetime behavior, yielding pronounced contrast among distinct cellular compartments (Figs. 7 and S6). Lifetime distributions were extracted from representative regions of interest in all images by visual inspection (Fig. 6). Interestingly, ADOTAs **1**–**6** exhibited markedly reduced lifetimes within mitochondria. These quenched τ_f_ values were significantly lower than those measured in any of the solvents studied, suggesting the presence of additional nonradiative decay pathways inside the organelle. Such quenching is unlikely to arise solely from variations in microenvironmental polarity or viscosity. Instead, it may reflect specific interactions with mitochondrial components—such as membrane-associated proteins, lipids, or redox-active species—that facilitate energy or electron transfer from the excited dye. In fact, both ADOTA and DAOTA dyes in the excited state are known to act as efficient electron acceptors, which results in fluorescence quenching^20,44^. These results are in excellent agreement with the energy calculated for the LUMO orbitals of all dyes (Figure S3), all of which are lower in energy than the reduction potential of NADH vs. vacuum (–4.76 eV), thus facilitating the quenching of the excited state through a redox reaction^45^. Therefore, the reducing environment of the mitochondrial matrix can be directly probed with our dyes using FLIM imaging. Mitochondrial accumulation may also be driven by the presence of mitochondrial G-quadruplex DNA structures^46^ to which ADOTA dyes can bind^18^.

Regarding nuclear accumulation, DAOTAs **7** and **8** clearly translocated to the nucleus, where they prominently stained the nucleoli. Likewise, upon enhancing image contrast, dyes **1**, **3**, and **4** also exhibited partial nuclear localization and apparent DNA binding. The τ_f_ values of these dyes within the nucleus and DNA-rich regions were relatively long, comparable to those measured in the extracellular medium (Figs. 7 and S6) and to the values obtained in aqueous solution (Fig. 4d). To further probe the nature of this interaction, we examined the fluorescence lifetimes of the dyes in the presence of calf thymus DNA (CT-DNA) in vitro. Binding to CT-DNA did not significantly affect the τ_f_ values (Figure S7), which remained high even in the presence of DNA. This observation is consistent with the FLIM data from HeLa cells, suggesting that nuclear binding does not induce strong quenching or perturbation of the excited-state dynamics.

We also extracted fluorescence lifetime distributions from small vesicular structures observed in cells stained with ADOTA **1** and DAOTAs **7** and **8** (Figs. 7 and S6). In all cases, the dyes exhibited consistently short fluorescence lifetimes within these organelles. As discussed above, this pronounced lifetime reduction likely arises from local aggregation or the formation of ion-pair complexes within the vesicular environment. Such behavior is consistent with partial dye aggregation in endosomal or lysosomal compartments, where confinement, local pH, and ionic strength may further promote these interactions.

Finally, we tested the usefulness of the dyes in other advanced microscopy techniques, i.e. state-of-the-art super-resolution microscopy, specifically structured illumination microscopy (SIM) and stimulated emission depletion (STED). In SIM experiments, we focused on DAOTA **7** due to its excellent nucleus internalization and high brightness. SIM images (Figure ﻿S8) exhibited excellent resolution levels for chromatin and nuclei staining. For STED experiments, we focused on the dyes with the largest mitochondrial accumulation (ADOTAs **3**–**6**). However, after several attempts to optimize the experimental conditions, resolution levels did not improve under STED mode in any of the cases (Figure S9), indicating low depletability of the excited dyes^47^.

## Conclusions

We report a novel microwave-assisted synthesis of triangulenium derivatives, offering a rapid and efficient route to a variety of substituted triangulenium dyes. The synthetic utility of this approach is demonstrated by the preparation of novel ADOTA and DAOTA derivatives with previously unexplored substitutions. Notably, this methodology drastically reduces reaction times while maintaining high yields. Reactions for ADOTA derivatives are completed within minutes, and for DAOTA, within hours—significantly shorter than conventional protocols that require several days. The synthetic methodology described here paves the way for the expansion of the palette of available ADOTAs and DAOTAs with long τ_f_ values, thereby broadening their application in biolabeling and bioimaging techniques.

These exceptionally long τ_f_ values make triangulenium dyes especially interesting for specific applications in FLIM imaging. To better understand the effects of the environment on fluorescence emission features, we fully characterized the photophysical properties of the new dyes in different solvents. It is important to note that the luminescent properties of the dyes are robust, exhibiting mild solvatochromism, mainly controlled by the polarizability of the solvent.

FLIM imaging of living HeLa cells demonstrated the excellent performance of the dyes, which also presented high brightness. The most relevant feature is the strong contrast provided by the dyes’ τ_f_ values across different subcellular localizations. Specifically, these triangulenium dyes accumulated in mitochondria, which is consistent with their positive charge and moderate hydrophobicity. Within this organelle, the dyes undergo electron-transfer-driven quenching, as both ADOTAs and DAOTAs can act as electron acceptors in the mild reducing environment of the mitochondria. This quenching results in lower τ_f_ values, thereby providing a unique contrast mechanism for the identification of mitochondria in FLIM imaging. Unlike conventional probes used in confocal or even FLIM microscopy, these dyes uniquely provide lifetime contrast across subcellular localizations when used as a single fluorescent probe. Our dyes exhibit lifetime variations of up to 10 ns across these different subcellular localizations, resulting in exceptional sensitivity in FLIM imaging. This work will foster further development of triangulenium dyes with similar lifetime features and a broader range of emission wavelengths, as well as their application in the design of biological sensors specifically tailored for FLIM imaging.

## Methods

### General procedure A. Microwave-assisted synthesis of acridinium salts 10–15

2,6-lutidine (1.90 equiv) was added to a solution of tris(2,6-dimethoxyphenyl)methylium^18^ **9** (1 equiv) and the corresponding primary amine (2 equiv) in acetonitrile (0.1 M). The mixture was heated to 80 °C in a Biotage Initiator + 2.0 microwave reactor using the standard absorbance level (400 W maximum power). When the reaction was completed, and after cooling to room temperature, an aqueous solution of NaBF_4_ (0.2 M) was added and extracted with dichloromethane. The organic layer was dried over Na_2_SO_4_, filtered and evaporated under reduced pressure. The residue was recrystallized from dichloromethane-diethyl ether, obtaining the desired products as a red powder. (See SI for the characterization of compounds **10**–**15**).

### General procedure B. Intramolecular ring close. Synthesis of ADOTA compounds 1–6

**Method B1.** A modification procedure to that reported by R. Vilar et al. was used^18^. In a round-bottom flask, pyridinium chloride (128 equiv) was added and heated at 200 °C until complete melting occurred. Then, the corresponding acridinium salt (1 equiv) was added and the mixture was heated at 200 °C for 2 h. The product was precipitated by the addition of a solution of NaBF_4_ 0.2 M and cooled to room temperature. The red precipitated was filtered off and washed with water. The solid was dissolved in acetonitrile and concentrated in vacuo. Then, the material was dissolved in dichloromethane, dried over Na_2_SO_4_, filtered and concentrated in vacuo, obtaining a red solid. The residue was recrystallized from acetonitrile-diethyl ether, obtaining the desired products as a red powder.

**Method B2.**^14^ In a round-bottom flask were added the corresponding acridinium salt (1mmol), pyridine hydrochloride (10 g) and pyridine (5 ml). The mixture was stirred at 140 °C for 5 h. Then, the reaction mixture was poured onto ice/water. The addition of HBF_4_·Et_2_O led to the formation of a red precipitate that was filtrated. The solid was recrystallized from acetonitrile-diethyl ether, obtaining the desired products as a red powder.

### General procedure C. Microwave-assisted synthesis of DAOTA dyes 7–8

In a 10 mL microwave vial, the corresponding ADOTA triangulenium (1 equiv) was added and dissolved in acetonitrile (0.08 M). Then, the corresponding primary amine (25–50 equiv) was added gradually. The mixture was heated to 100 °C in a Biotage Initiator + 2.0 microwave reactor using the standard absorbance level (400 W maximum power). Reaction time depends on the nature of the substituent (From 1 h to 2 h). Then, the reaction was cooled at room temperature and the solvent was removed *in vacuo* (see the SI for the characterization of compounds **7**–**8**).

### Spectrocopy methods

Absorption spectra were collected on a Cary 4000 (Agilent Technologies) spectrophotometer. Emission spectra were acquired on a Jasco FP-8300 spectrofluorometer. Samples were excited at two different wavelengths, separated by approximately 10 nm to verify spectral consistency and provide independent measurements of quantum yields. Time-resolved fluorimetry was performed on a PicoQuant FluoTime 200, equipped with a 530 nm pulsed laser (Picoquant, P-FA-530B), working at a repetition rate of 5 MHz, as set by a PDL-800 laser driver (PicoQuant), providing a 200 ns temporal detection window. Full details of the methods can be found in the SI.

### DFT calculations

Electronic structure Density Functional Theory (DFT) calculations were performed using the ORCA 6.0.1 package, employing the dispersion-corrected wB97X-D4 functional, with def2-TZVP Ahlrichs basis set and the corresponding auxiliary basis sets were used for all atoms, and taking advantage of the Resolution of Identity approximation for coulomb and HF exchange integrals (RIJCOSX approximation). Full details are described in the SI.

### Cell microscopy and image analysis

HeLa cells were cultured and seeded in 8-well µ-slides (Ibidi) for imaging as described in the SI. FLIM and STED imaging were performed using an Abberior Expert Line instrument (Abberior Instruments GmbH, Heidelberg, Germany) based on an Olympus IX-71 confocal microscope, equipped with an UPlanSApo 100× oil-immersion objective (NA 1.4), and a Multiharp 150 4 N time-correlated single-photon counting (TCSPC) module (PicoQuant GmbH, Germany). For FLIM, triangulenium dyes were excited with a 485-nm pulsed laser, working at 5 MHz for a TCSPC time window of 200 ns, and detected on an avalanche photodiode (APD) after a 605/50 nm bandpass filter. Colocalization experiments employed a second excitation laser at 640 nm for MitoTracker Deep Red, MT (Sigma Aldrich) mitochondria staining dye, and its emission collected on a second APD after a 685/70 nm bandpass filter. For STED imaging, a pulsed, 775-nm depletion laser was employed. Further instrumental details can be read in the SI.

Colocalization analysis was performed using Fiji^48^ (distribution of ImageJ) for constructing RGB overlaid images and estimating the Mander’s colocalization coefficients as the fraction of colocalized pixels with respect to the total selected pixels for each channel, using an automatic intensity threshold, based on the *moments* algorithm. The average values from at least 10 different images were obtained for each triangulenium dye derivative.

FLIM images were analyzed using SymphoTime 64 software (PicoQuant), by performing pixelwise fittings of the fluorescence decay traces to a biexponential decay function, using an iterative reconvolution method and an instrument response function (IRF) calculated from the global traces. First, a 2 × 2 spatial binning and an 8-channel time binning in the TCSPC scale was performed to enhance the individual decay traces. Finally, the FLIM images were reconstructed by adjusting the intensity (grayscale) and lifetime (color) scales in Fiji , using the intensity-weighted average lifetime from the biexponential fitting. Lifetime distributions were obtained by manual selection of ROIs in Fiji, differentiating between mitochondria, vesicular organelles, nuclei, condensed DNA or the extracellular medium in dyes with slow cellular uptake. The lifetime distributions of ROIs of the same nature were added up from all the collected images of each dye.

Super-resolution structured illumination microscopy (SIM) was performed on a Nikon AX system built upon a Nikon Eclipse Ti2, equipped with multi-line lasers, Nikon D-LEDI multi-line LEDs and Hamamatsu ORCA Flash 4.0 sCMOS monochromatic sensor camera. HeLa cells were fixed upon 1 h incubation with the dye, using *p*-formaldehyde and ProLong™ Diamond antifade mountant medium.

### Supporting information available

The Supporting Information file (PDF) includes supplementary experimental section, detailed information on the synthesis and characterization of the new dyes, including^1^H- and^13^C-NMR spectra, Supporting Figures S1-S9, and Supporting Tables S1-S10. Additional Supporting Information files include data sheets with xyz coordinates from TD-DFT geometry optimization of the 8 dyes (XLSX), and the results from the fits of fluorescence decay traces of all the dyes in all the tested solvents (XLSX), including decay times, relative amplitudes and, χ^2^ values.

## Supplementary Information

Below is the link to the electronic supplementary material.

Supplementary Material 1

Supplementary Material 2

Supplementary Material 3

## Data Availability

The datasets generated during and/or analysed during the current study are available from the corresponding author on reasonable request.

## Declarations

### Notes

This paper is dedicated to Dr. Rosario Herranz, whose leadership and mentorship have been a source of inspiration.
