# Supplementary material for: Microwave-assisted synthesis of new triangulenium dyes for lifetime imaging microscopy (FLIM)
Source: Sci Rep. 2025 Dec 21;16:1925. doi: 10.1038/s41598-025-31688-6 (PMC12804676; doi:10.1038/s41598-025-31688-6)
Supplement: Supplementary file 3 — Supplementary Material 3 [file 41598_2025_31688_MOESM3_ESM.pdf]

New triangulenium dyes for lifetime imaging microscopy (FLIM)

Carmen Viedma-Barba[1], Esther M. Ortega-Naranjo[2], Federico Movilla,[2] Antonio Reinoso[3], Marta Andrades-Amate[2], Pablo Peñalver[2,4], Juan C. Morales[4], Juan A. González-Vera[2], Marta Gutiérrez-Rodríguez[1,], Angel Orte[2,*]*

[1] Instituto de Química Médica, IQM-CSIC, 28006, Madrid.

[2] Nanoscopy-UGR Lab, Dept. Fisicoquímica, Facultad de Farmacia, Universidad de Granada. 18071, Granada (Spain).

[3] Departamento de Bioquímica y Biología Molecular II, Facultad de Farmacia, Universidad de Granada. 18071, Granada (Spain).

[4] Instituto de Parasitología y Biomedicina López-Neyra, CSIC, Granada.

E-mail: mgutierrez@iqm.csic.es ; angelort@ugr.es

Supplementary Experimental Section 3
General Information 3
Synthesis of intermediate reagents and novel ADOTA and DAOTA dyes **1-8** 4
Spectroscopy measurements 11
DFT calculations 11
Cell microscopy and image analysis 12
Photophysical properties of compounds 1-8 14
Table S1. Photophysical properties of ADOTA **1** in different solvents. 14
Table S2. Photophysical properties of ADOTA **2** in different solvents. 15
Table S3. Photophysical properties of ADOTA **3** in different solvents. 16
Table S4. Photophysical properties of ADOTA **4** in different solvents. 17
Table S5. Photophysical properties of ADOTA **5** in different solvents. 18
Table S6. Photophysical properties of ADOTA **6** in different solvents. 19
Table S7. Photophysical properties of DAOTA **7** in different solvents. 20
Table S8. Photophysical properties of DAOTA **8** in different solvents. 21

Figure S1. Representative k_f (black symbols) and k_{nr} (red symbols) values of dyes **1-8** in selected solvents. The radiative, k_f and nonradiative, k_{nr}, rates demonstrated mostly invariable values for k_f ((34±4)×10^6 s^{-1} for most ADOTAs and (28±5)×10^6 s^{-1} for DAOTAs). A particular case was ADOTA **5** that exhibited lower Φ_f values and k_f rates in the range of (11±5)× 10^6 s^{-1}. Interestingly, k_{nr} rates exhibited dependence with the solvent, increasing its value in more apolar solvents. 22

Solvatochromism analysis 23

Figure S2. Plots of the weight of the coefficients of the solvents scales, Sdp (red), Sp (gray), SA (blue), and SB (pink), in the correlations of νabs (**a**), νem (**b**), and τ_f (**c**) for dyes **1-8**. **d**) Experimental and predicted λ_{abs} (circles) and λ_{em} (diamonds) of dyes **1-8** in different solvents, as obtained from the solvatochromic analysis. The solvent scale parameters employed in the estimation of the predicted values are those indicated in each case. 24

Molecular orbitals of triangulenium dyes 1-8 25

Figure S3. Canonical representation of the frontier orbitals (HOMO and LUMO) for the 8 dyes and their corresponding energy gap. 25

Lipophilicity of dyes 1-8 26

Table S9. Estimated logP values for dyes **1-8** using different algorithms. 26

Cytotoxicity of dyes 1-8 27

Table S10. EC_{50} values of dyes **1-8** in HeLa and MRC5 cells. 27

Supporting cell microscopy figures 28

Figure S4. FLIM images of dye **2** (λ_{ex} = 485 nm, detection bandpass at 605/50 nm) in HeLa cells to study uptake kinetics. The dye was added at time 0, and images were acquired every 10 min. The intensity quantification plot is also shown, exhibiting the photostability of the dye. 28

Figure S5. Additional colocalization imaging of dyes **1-8** (green channel, λ_{ex} = 485 nm, detection bandpass at 605/50 nm) and MT (red channel, λ_{ex} = 485 nm, detection bandpass at 685/70 nm) in HeLa cells. The right column of images shows the binary masks in both channels and colocalized pixels in yellow for the quantification of colocalized areas. 29

Figure S6. Additional representative FLIM images of ADOTAs **1-6** and DAOTAs **7-8** (2 μM) in HeLa cells (λ_{ex} = 485 nm; detection band 580-630 nm). The pseudocolor scale represents the τ_f values. Scale bars represent 10 μm. Representative images of autofluorescence are also shown, although their intensity is enhanced 7-fold to illustrate the low τ_f values. 30

Interaction of 1-8 with CT-DNA 31

Figure S7. Fluorescence lifetime τ_f of ADOTAs **1-6** and DAOTAs **7** and **8** (1.2 μM) in the absence and the presence of CT-DNA (1.2 μM bp). λ_{ex} = 530 nm. 31

Super-resolution microscopy images 32

Figure S8. Representative super-resolution SIM images of dye **7** (2 μM) in fixed HeLa cells (λ_{ex} = 561 nm; detection at 615 nm). 32

Figure S9. Comparative images in confocal and STED mode of ADOTA dyes **3–6** (2 μM) in HeLa cells (λ_{ex} = 485 nm; depletion laser at 775 nm; detection at 615 nm). 33

Annex 1. NMR spectra 34

References 48

Supplementary Experimental Section

General Information

All reagents were commercially sourced and used without further purification. Microwave reactions were performed using Biotage Initiator+ 2.0. Experiments were carried out in sealed microwave process vials utilizing the standard absorbance level (400 W maximum power). The temperature was measured with an IR sensor on the outside of the reaction vessel. Silica gel 60 F254 precoated plates (Merck, 0.2 mm layer thickness) were used for TLC and visualized with ultraviolet light of wavelength 254 nm and/or 365 nm. Flash chromatography was performed manually using silica gel 60 (40–64 μm230-400 mesh ASTM) or aluminum oxide as the stationary phase. The isolated products were characterized by NMR, HPLC, liquid chromatography mass spectrometry (HPLC-MS) and high-performance mass spectrometry (HRMS). The final products obtained were lyophilized on a virtis BenchTop Pro. Analytical HPLC chromatograms were recorded on Waters equipment with a Sunfire C18 column (3.5 μm, 4.6 mm x 50 mm), a flow rate of 1.5 mL/min and an appropriate gradient of A (CH_3CN) in B (H_2O) with 0.05% TFA as additive. Peak detection was performed at a wavelength of 220 and/or 254 nm. HPLC-MS chromatograms were obtained with a Waters 2695 coupled to a Waters Micromass ZQ spectrometer. The separation was performed with a Sunfire C18 column (3.5 μm, 4.6 mm x 50 mm), a flow rate of 1.0 mL/min and as mobile phase, an appropriate gradient of A (CH_3CN) in B (H_2O) with 0.1% formic acid as additive. Mass spectra were obtained using the electrospray ESI technique. High Resolution Mass Spectrum (HRMS) was performed in an Agilent 6520 Accurate-Mass Q-TOF LC-MS equipped with a liquid chromatograph HP-1260 Infinity II coupled to a mass spectrometer with hybrid mass analyzer Q-TOF 6546. ACN:H_2O (75:25, v:v) was used as mobile phase at 0.5 mL/min. The ionization source was an ESI interface working in the positive or negative ion mode. The electrospray voltage was set at 4.5 kV, the fragmentor voltage at 150 V and the drying gas temperature at 225 °C. Nitrogen (99.5% purity) was used as nebulizer (138 kPa) and drying gas (10 L/min). Usually, an error equal or lower than 5 ppm is allowed. In this technique $[M+H]^+$, $[M+NH_4]^+$ and $[M+Na]^+$ or $[M-H]^-$, $[M-2H]^{2-}$, $[M-3H]^{3-}$ and $[M-4H]^{4-}$ peaks, for positive or negative mode respectively, are the most significant signals along with [M]. NMR spectra (^1H and ^{13}C) were mainly performed in CD_3CN, DMSO-d_6, MeOD and $CDCl_3$ at room temperature on a Bruker-400 or JEOL-400 spectrometers. The chemical shifts (δ) are expressed in parts per million (ppm) taking as reference the signal of the used solvent. Multiplicities of the ^1H NMR signals are denoted by s(singlet), d (doublet), dd (doublet of doublet), dt (doublet of the triplet), t (triplet), quin (quintet), m (multiplet), br.s (broad singlet).

Synthesis of intermediate reagents and novel ADOTA and DAOTA dyes **1-8**

The following sections describe the synthesis reactions and protocols to obtain the intermediate reagents and the final products 1-8. After purification, as detailed in the main text, NMR spectra (^{1}H and ^{13}C) were obtained for characterization, performed in CD_3CN, DMSO-d_6, MeOD and $CDCl_3$ at room temperature. The chemical shifts (δ) are expressed in parts per million (ppm) taking as reference the signal of the used solvent. Multiplicities of the ^{1}H NMR signals are denoted by s (singlet), d (doublet), dd (doublet of doublet), dt (doublet of the triplet), t (triplet), quin (quintet), m (multiplet), br.s (broad singlet).

Synthesis of 9. Tris(2,6-dimethoxyphenyl)methylium, compound **9**, was prepared according to a previously published procedure.[1]

General procedure A for the synthesis of the acridinium salts 10-15. To a solution of tris(2,6-dimethoxyphenyl)methylium **9** (1.00 equiv) and the corresponding primary amine (2.00 equiv) in acetonitrile (0.1 M), 2,6-lutidine (1.90 equiv) was added. The mixture was heated to 80°C in a Biotage Initiator+ 2.0 microwave reactor using the standard absorbance level (400 W maximum power). When the reaction is completed, and after cooling to room temperature, an aqueous solution of $NaBF_4$ (0.2 M) was added and extracted with dichloromethane. The organic layer was dried over Na_2SO_4, filtered and evaporated under reduced pressure. The residue was recrystallized from dichloromethane-diethyl ether, obtaining the desired products as a red powder.

*10-(3-cyanophenyl)-9-(2,6-dimethoxyphenyl)-1,8-dimethoxy-acridin-10-ium tetrafluoroborate (**10**)*

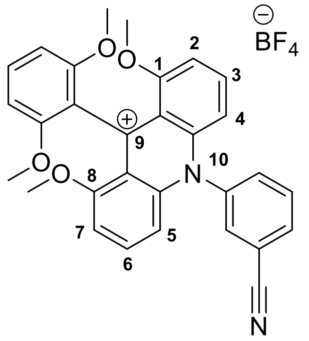

Following the general procedure A, compound **10** was prepared from **9** (797.7 mg, 1.56 mmol, 1.00 equiv) and 3-aminobenzonitrile (368.6 mg, 3.12 mmol, 2.00 equiv). After 2 h, the reaction was completed and **10** was obtained as a red powder. Yield: 860.3 mg (98 %). **HPLC** (Sunfire C_{18}, 15-95% of A in B, 10 min); t_R = 5.66 min. **LC-MS (m/z)**: 478 $[M+H]^+$. **^{1}H NMR (400 MHz, CD_3CN) δ (ppm)**: 8.25 – 8.21 (m, 1H, *H2 cyanophenyl*), 8.07 – 7.90 (m, 7H, *H4, H5 and H6 cyanophenyl*, *Acridinium H3, H2, H6 and H7*), 7.48 (t, J = 8.4 Hz, 1H, *Ar H4*), 7.10 (d, J = 8.1 Hz, 2H*, Acridinium H4 and H5*), 6.89 – 6.80 (m, 2H, *Ar H3 and H5*), 3.61 (s, 6H, *OMe*), 3.58 (s, 6H, *OMe*). **^{13}C NMR (100 MHz, CD_3CN) δ (ppm)**: 161.04, 160.68, 156.40, 156.34, 143.35, 140.62, 139.84, 135.79, 133.49, 133.42, 132.54, 120.17, 115.96, 111.37, 107.35, 104.39, 56.29.

*10-(4-acetamidophenyl)-9-(2,6-dimethoxyphenyl)-1,8-dimethoxy-acridin-10-ium tetrafluoroborate (**11**)*

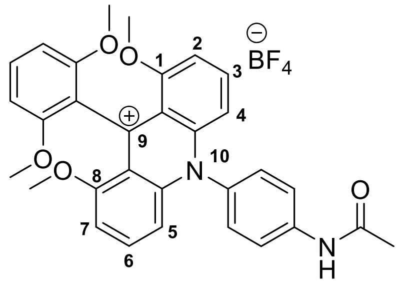

Following the general procedure A, compound **11** was prepared from **9** (100 mg, 0.19 mmol, 1.00 equiv) and 4-aminoacetanilide (57.1 mg, 0.38 mmol, 2.00 equiv). After 35 min, the reaction

was completed and **11** was obtained as a red powder. Yield: 98.4 mg (87 %). **HPLC** (Sunfire C_{18}, 20-95% of A in B, 5 min); t_R = 4.42 min. **LC-MS (m/z)**: 509 $[M+H]^+$. **^{1}H NMR (400 MHz, CD_3CN) δ (ppm)**: 8.82 (s, 1H, *NH*), 8.07 – 8.02 (m, 2H, *Acridinium H4 and H5*), 7.96 (dd, J = 9.0, 8.0 Hz, 2H, *Acridinium H3 and H6*), 7.53 – 7.44 (m, 3H, *H3 and H5 acetamidophenyl; Ar H4*), 7.07 (dd, J = 8.2, 0.9 Hz, 2H, *Acridinium H2 and H7*), 6.99 (dd, J = 9.1, 0.9 Hz, 2H, *Ar H3 and H5*), 6.81 (d, J = 8.4 Hz, 2H, *H2 and H6 acetamidophenyl*), 3.60 (s, 6H, *OMe*), 3.56 (s, 6H, *OMe*), 2.18 (s, 3H, *CH_3*). **^{13}C NMR (100 MHz, CD_3CN) δ (ppm)**: 170.28, 161.10, 159.74, 156.77, 144.20, 142.64, 140.38, 134.27, 130.56, 129.24, 122.35, 122.01, 120.60, 120.31, 112.04, 107.44, 104.67, 57.75, 56.59, 24.44.

*9-(2,6-dimethoxyphenyl)-1,8-dimethoxy-10-(pyridin-3-yl)-acridin-10-ium tetrafluoroborate (**12**)*

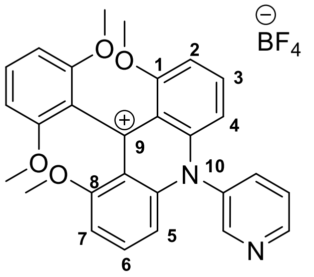

Following the general procedure A, compound **12** was prepared from **9** (100 mg, 0.19 mmol, 1.00 equiv) and 3-aminopyridine (35.8 mg, 0.38 mmol, 2.00 equiv). After 45 min, the reaction was completed and **12** was obtained as a red powder. Yield: 101.1 mg (98 %). **HPLC** (Sunfire C_{18}, 20-95% of A in B, 5 min); t_R = 3.82 min. **LC-MS (m/z)**: 454 $[M+H]^+$. **^{1}H NMR (400 MHz, CD_3CN) δ (ppm)**: 9.07 (dd, J = 4.8, 1.5 Hz, 1H, *Pyridine H2*), 8.79 (dd, J = 2.6, 0.8 Hz, 1H, pyridine H4), 8.06 (ddd, J = 8.2, 2.6, 1.5 Hz, 1H, *pyridine H6*), 7.99 (dd, J = 9.0, 8.1 Hz, 2H, *Acridinium H3 and H6*), 7.89 (ddd, J = 8.2, 4.8, 0.8 Hz,1H, *pyridine H5*), 7.48 (t, J = 8.4 Hz, 1H, *Ar H4*), 7.10 (d, J = 8.1 Hz, 2H, *Acridinium H2 and H7*), 6.88 (dd, J = 9.0, 0.8 Hz, 2H, *Ar H3 and H5*), 6.82 (d, J = 8.4 Hz, 2H, *Acridinium H4 and H5*), 3.61 (d, J = 1.6 Hz, 6H, *OMe*), 3.58 (s, 6H, *OMe*).**^{13}C NMR (100 MHz, CD_3CN) δ (ppm)**: 161.40, 160.98, 156.73, 153.40, 149.42, 144.09, 140.98, 137.03, 136.84, 130.72, 126.91, 120.55, 120.17, 111.66, 107.70, 104.74, 57.87, 56.64.

*9-(2,6-dimethoxyphenyl)-1,8-dimethoxy-10-(pyridin-3-ylmethyl)-acridin-10-ium tetrafluoroborate (**13**):*

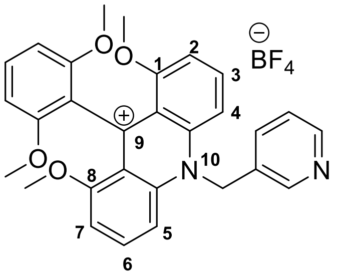

Following the general procedure A, compound **13** was prepared from **9** (100 mg, 0.19 mmol, 1.00 equiv) and 3-pycolylamine (38.7 µL, 0.38 mmol, 2.00 equiv). After 1 min, the reaction was completed and **13** was obtained as a red powder. Yield: 80.5 mg (76 %). **HPLC** (Sunfire C_{18}, 15-95% of A in B, 10 min); t_R = 4.61 min. **LC-MS (m/z)**: 468 $[M+H]^+$. **^{1}H NMR (400 MHz, CD_3CN) δ (ppm)**: 8.60 (dd, *J* = 4.9, 1.6 Hz, 1H, *Pyridine H2*), 8.56 (d, *J* = 2.5 Hz, 1H, *Pyridine H4*), 8.12 (dd, *J* = 9.1, 8.1 Hz, 2H, *Acridinium H3 and H6*), 7.70 (m, 2H, *Ar H4 and Pyridine H6*), 7.52 – 7.42 (m,2H, *Ar H3 and H5*), 7.38 – 7.33 (m, 1H, *Pyridine H5*), 7.11 (d, *J* = 8.1 Hz, *2H, Acridinium H2 and H7*), 6.80 (d, *J* = 8.4 Hz, 2H, *Acridinium H4 and H5*), 6.34 (s, 2H, *CH_2*), 3.59 (s, 6H, *OMe*), 3.56 (s, 6H, *OMe*). **^{13}C NMR (100 MHz, CD_3CN) δ (ppm)**: 161.79, 160.15, 150.62, 148.62, 143.34, 141.64, 134.66, 130.56, 130.35, 124.84, 124.25, 120.98, 120.31, 110.12, 107.75, 104.65, 57.79, 56.58, 55.21.

*9-(2,6-dimethoxyphenyl)-1,8-dimethoxy-10-(pyridin-4-ylmethyl)-acridin-10-ium tetrafluoroborate (**14**)*

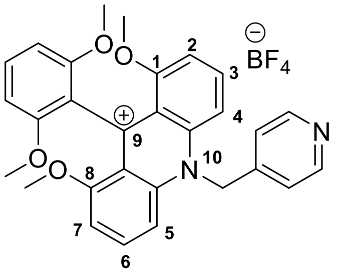

Following the general procedure A, compound **14** was prepared from **9** (100 mg, 0.19 mmol, 1.00 equiv) and 4-pycolylamine (38.7 µL, 0.38 mmol, 2.00 equiv). After 1 min, the reaction was completed and **14** was obtained as a red powder. Yield: 93.6 mg (89 %). **HPLC** (Sunfire C_{18}, 15-95% of A in B, 5 min); t_R = 3.56 min. **LC-MS (m/z)**: 468 $[M+H]^+$. **^{1}H NMR (400 MHz, CD_3CN) δ (ppm)**: 8.61 – 8.58 (m, 2H, *pyridine H5 and H3*), 8.11 (dd, J = 9.1, 8.1 Hz, 2H, *Acridinium H3 and H6*), 7.62 (dd, J = 9.2, 0.8 Hz, 2H, *Acridinium H2 and H7*), 7.46 (t, J = 8.4 Hz, 1H, *Ar H4*), 7.21 – 7.16 (m, 2H, *pyridine H2 and H6*), 7.11 (d, J = 8.0 Hz, 2H, *Acridinium H4 and H5*), 6.80 (d, J = 8.3 Hz, 2H, *Ar H3 and H5*), 6.32 (s, 2H, *CH_2*), 3.59 (s, 6H, *OMe*), 3.57 (s, 6H, *OMe*). **^{13}C NMR (100 MHz, CD_3CN) δ (ppm)**: 161.79, 160.34, 156.63, 151.44, 143.44, 143.30, 141.67, 130.58, 121.95, 120.90, 110.03, 107.76, 104.63, 57.79, 56.57, 56.21.

*9-(2,6-dimethoxyphenyl)-1,8-dimethoxy-10-(pyridin-2-ylmethyl)-acridin-10-ium tetrafluoroborate (**15**)*

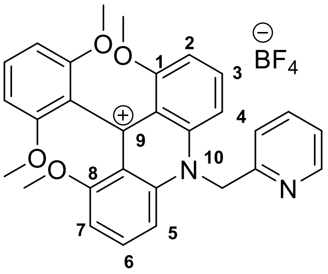

Following the general procedure A, compound **15** was prepared from **9** (100 mg, 0.19 mmol, 1.00 equiv) and 2-pycolylamine (39.2 µL, 0.38 mmol, 2.00 equiv). After 5 min, the reaction was completed and **15** was obtained as a red powder. Yield: 81.9 mg (78 %). **HPLC** (Sunfire C_{18}, 20-95% of A in B, 5 min); t_R = 4.35 min. **LC-MS (m/z)**: 468 $[M+H]^+$. **^{1}H NMR (400 MHz, CD_3CN) δ (ppm)**: 8.35 (ddd, J = 4.9, 1.8, 1.0 Hz, 1H, *pyridine H3*), 8.08 (dd, J = 9.2, 8.1 Hz, 2H, *Acridinium H3 and H6*), 7.92 (td, J = 7.7, 1.7 Hz, 1H, *Ar H4*), 7.68 (ddd, J = 20.7, 8.6, 0.9 Hz, 3H, *Pyridine H5 and acridinium H7 and H7*), 7.46 (t, J = 8.4 Hz, 1H, *pyridine H4*), 7.36 (ddd, J = 7.6, 4.9, 1.1 Hz, 1H, *pyridine H6*), 7.08 (d, J = 8.0 Hz, 2H, *acridinium H4 and H5*), 6.80 (d, J = 8.4 Hz, 2H, *Ar H3 and H5*), 6.43 (s, 2H, *CH_2*), 3.59 (s, 6H, *OMe*), 3.55 (s, 6H, *OMe*). **^{13}C NMR (100 MHz, CD_3CN) δ (ppm)**: 161.49, 159.29, 156.68, 153.74, 150.59, 143.55, 141.05, 138.55, 130.48, 124.56, 122.98, 120.72, 120.39, 110.67, 107.57, 104.64, 57.78, 57.65, 56.61.

General procedure B for the synthesis of ADOTA compounds **1-6**

Method B1. A modification procedure to that reported by R. Vilar *et al* was used.[1] In a round-bottom flask was added the pyridinium chloride and heating at 200°C, until complete melting occurred. Then, the corresponding acridinium salt (1.00 equiv) was added and heating at 200 °C for 2 h. The product was precipitated by the addition of a solution of $NaBF_4$ 0.2 M and cooled to room temperature. The red precipitated was filtered off and washed with water. The solid was dissolved in acetonitrile and concentrated *in vacuo*. Then, the material was dissolved in dichloromethane, dried over Na_2SO_4, filtered and concentrated *in vacuo*, obtaining a red solid. The residue was recrystallized from acetonitrile-diethyl ether, obtaining the desired products as a red powder.

Method B2.[2] In a round-bottom flask were added the corresponding acridinium salt (1.00 equiv), pyridine hydrochloride and were dissolved in pyridine 0.2 M. The mixture was stirred at 140 °C for 5 h. Then, the reaction mixture was poured onto ice/water. The addition of $HBF_4 \cdot Et_2O$ lead to the formation of a red precipitate, that was filtrated. The residue was recrystallized from acetonitrile-diethyl ether, obtaining the desired products as a red powder.

*4-(3-cyanophenyl)-4-aza-8,12-dioxatriangulenium tetrafluoroborate (**1**):*

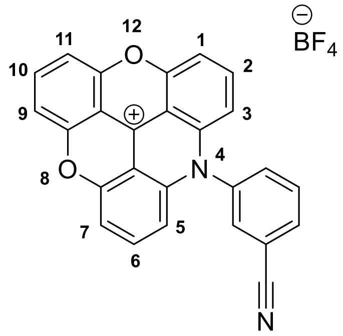

Following the general procedure B1, compound **1** was prepared from **10** (524 mg, 0.93 mmol, 1.00 equiv) and pyridinium chloride (13.8 g). Yield: 247 mg (80 %). **HPLC** (Sunfire C_{18}, 15-95% of A in B, 10 min); t_R = 4.72 min. **LC-MS (m/z)**: 386 $[M+H]^+$. **^{1}H NMR (400 MHz, CD_3CN) δ (ppm)**: 8.24 – 8.13 (m, 4H, *H2, H4, H5 and H6 cyanophenyl*), 8.03 (dd, J = 16.8, 8.8 Hz, 2H, *Ar H2 and H6*), 7.89 (dd, J = 8.0, 1.0 Hz, 2H, *Ar H10*), 7.60 (d, J = 1.5 Hz, 2H, *Ar H1 and H7*), 7.58 (d, J = 1.8 Hz, 2H, *Ar H3 and H5*), 6.89 (d, J = 8.8 Hz, 2H, *Ar H9 and H11*). **^{13}C NMR (100 MHz, CD_3CN) δ (ppm)**: 153.66, 153.39, 142.97, 142.36, 141.61, 141.23, 137.94, 135.72, 134.00, 133.93, 133.05, 116.37, 112.53, 111.72, 110.48, 108.98, 106.52. **HR-MS (ESI-TOF)** m/z: [M+] Calcd for $C_{26}H_{13}O_2N_2^+$, 385.0977, found 385.0976.

*4-(4-acetamidophenyl)-4-aza-8,12-dioxatriangulenium tetrafluoroborate (**2**)*

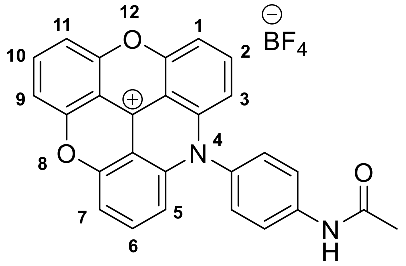

Following the general procedure B2, compound **2** was prepared from **11** (79.6 mg, 0.13 mmol, 1.00 equiv) and pyridinium chloride (1.3 g). The red solid was recrystallized several times with dichloromethane-pentane. Yield: 41.5 mg (76 %).**HPLC** (Sunfire C_{18}, 20-95% of A in B, 5 min); t_R = 3.42 min. **LC-MS (m/z)**: 418 $[M+H]^+$. **^{1}H NMR (400 MHz, CD_3CN) δ (ppm)**: 8.80 (s, 1H, *NH amide*), 8.20-8.11 (m, 3H, *Ar H2, H6 and H10*), 8.07 – 8.03 (m, 2H, *H3 and H5 acetamidophenyl*), 7.56 (d, J = 1.7 Hz, 2H, *Ar H9 and H11*), 7.54 (d, J = 1.4 Hz, 2H, *Ar H1 and H7*), 7.50 – 7.46 (m, 2H, *Ar H3 and H5,*), 6.98 (d, J = 8.8 Hz, 2H, *H2 and H6 acetamidophenyl*), 2.17 (s, 3H, *CH_3*). **^{13}C NMR (100 MHz, CD_3CN) δ (ppm)**: 170.21, 153.76, 153.60, 143.25, 142.54, 141.61, 141.39, 141.14, 132.15, 129.38, 122.51, 112.73, 112.48, 112.25, 110.35, 109.41, 24.41. **HR-MS (ESI-TOF)** m/z: [M+] Calcd for $C_{27}H_{17}O_3N_2^+$, 417.1239, found 417.1238.

*4-(pyridin-3-yl)-4-aza-8,12-dioxatriangulenium tetrafluoroborate (**3**)*

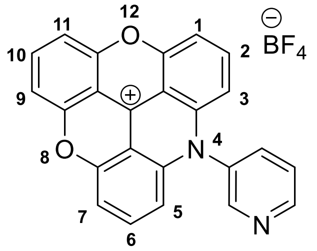

Following the general procedure B1, compound **3** was prepared from **12** (81.6 mg, 0.15 mmol, 1.00 equiv) and pyridinium chloride (2.50 g). Yield: 47.9 mg (71 %). **HPLC** (Sunfire C_{18}, 2-95% of A in B, 5 min); t_R = 4.80 min. **LC-MS (m/z)**: 362 $[M+H]^+$. **^{1}H NMR (400 MHz, CD_3CN) δ (ppm)**: 9.08 – 9.02 (m, 1H, *pyridine H2*), 8.83 (d, J = 2.5 Hz, 1H, *Ar H10*), 8.74 – 8.72 (m, 2H, *Ar H2 and H6*), 8.58 (tt, J = 7.9, 1.6 Hz, 1H, *pyridine H6*), 8.22 – 8.08 (m, 2H, *Ar H3 and H5*), 8.06 – 8.02 (m, 2H, *Ar H1 and H7*), 7.93 (dd, J = 8.2, 4.9 Hz, 1H, *pyridine H4*), 7.57 (dd, J = 8.4, 4.5 Hz, 2H, *Ar H9 and H11*), 6.91 (d, J = 8.8 Hz, 1H, *pyridine H5*). **^{13}C NMR (100 MHz, CD_3CN) δ (ppm)**: 153.95, 153.65, 152.63, 149.56, 148.10, 143.28, 142.96, 142.21, 141.96, 141.53, 138.52, 128.47, 127.72, 112.83, 111.96, 110.81, 109.30, 57.87, 56.64. **HR-MS (ESI-TOF)** m/z: [M+] Calcd for $C_{24}H_{13}O_2N_2^+$, 361.0977, found 361.0976.

*4-(pyridin-3-ylmethyl)-4-aza-8,12-dioxatriangulenium tetrafluoroborate (**4**)*

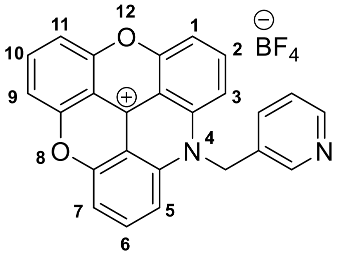

Following the general procedure B1, compound **4** was prepared from **13** (57.1 mg, 0.10 mmol, 1.00 equiv) and pyridinium chloride (1.74 g). After 30 min, the reaction was complete. The crude was purified by column chromatography using a mixture of dichloromethane:methanol (from 100:0 to 80:20). Yield: 8.50 mg (18 %). **HPLC** (Sunfire C_{18}, 2-95% of A in B, 10 min); t_R = 5.54 min. **LC-MS (m/z)**: 376 $[M+H]^+$. **^{1}H NMR (400 MHz, CD_3CN) δ (ppm)**: 8.71 (d, J = 5.9 Hz, 1H, *pyridine H2*), 8.44 (d, J = 8.3 Hz, 1H, *pyridine H4*), 8.32 (t, J = 8.6 Hz, 2H, *Ar H2 and H6*), 8.23 (t, J = 8.5 Hz, 1H, *Ar H10*), 8.03 (dd, J = 8.3, 5.8 Hz, 1H, *pyridine H5*), 7.67 – 7.59 (m, 7H, *pyridine H6 and Ar H1, H3, H5, H7, H9, H11*), 6.16 (s, 2H, *CH_2*). **^{13}C NMR (100 MHz, CD3CN) δ (ppm)**: 154.58, 153.49, 146.34, 142.57, 142.39, 141.76, 141.55, 140.68, 135.68, 128.83, 112.76, 111.17, 110.76, 109.82, 49.82. **HR-MS (ESI-TOF)** m/z: [M+] Calcd for $C_{25}H_{15}O_2N_2^+$, 375.1134, found 375.1134.

*4-(pyridin-4-ylmethyl)-4-aza-8,12-dioxatriangulenium tetrafluoroborate (**5**)*

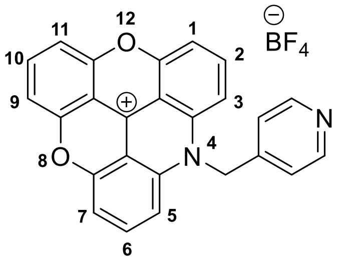

Following the general procedure B2, compound **5** was prepared from **14** (66.7 mg, 0.12 mmol, 1.00 equiv) and pyridinium chloride (1.3 g). The crude was purified by column chromatography using a mixture of dichloromethane:methanol (from 100:0 to 80:20). Yield: 31.1 mg (56 %). **HPLC** (Sunfire C_{18}, 2-95% of A in B, 5 min); t_R = 4.45 min. **LC-MS (m/z)**: 376 $[M+H]^+$. **^{1}H NMR (500 MHz, CD_3OD) δ (ppm)**: 9.00 (d, J = 6.0 Hz, 2H, *pyridine H2 and H6*), 8.59 (t, J = 8.0 Hz, 2H, *Ar*

H2 and H6), 8.33 (dd, J = 8.8, 8.2 Hz, 1H, *Ar H10*), 8.23 (t, J = 8.5 Hz, 2H, *pyridine H3 and H5*), 7.76 (d, J = 8.9 Hz, 2H, *Ar H3 and H5*), 7.67 (d, J = 8.2 Hz, 3H, *Ar H1 and H7*), 7.64 (d, J = 8.0 Hz, 2H, *Ar H9 and H11*), 6.20 (s, 2H, *CH_2*). **^{13}C NMR (100 MHz, CD_3OD) δ (ppm)**: 155.11, 154.10, 143.53, 142.51, 142.45, 141.30, 129.54, 112.81, 111.18, 110.97, 110.36, 107.35, 30.76. **HR-MS (ESI-TOF)** m/z: [M+] Calcd for $C_{25}H_{15}O_2N_2^+$, 375.1134, found 375.1133.

*4-(pyridin-2-ylmethyl)- 4-aza-8,12-dioxatriangulenium tetrafluoroborate (**6**):*

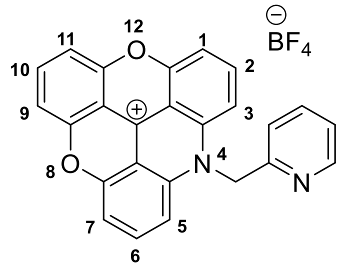

Following the general procedure B1, compound **6** was prepared from **15** (139 mg, 0.25 mmol, 1.00 equiv) and pyridinium chloride (4.23 g). After 30 min, the reaction was complete. Yield: 63.6 mg (55%). **HPLC** (Sunfire C_{18}, 2-95% of A in B, 5 min); t_R = 4.95 min. **LC-MS (m/z)**: 376 [M+H]$^+$. **^{1}H NMR (400 MHz, CD_3CN) δ (ppm)**: 8.41 (d, *J* = 4.8 Hz, 1H, *pyridine H3*), 8.27 (t, *J* = 8.5 Hz, 2H, *Ar H2 and H6*), 8.15 (td, *J* = 8.5, 1.5 Hz, 1H, *pyridine H5*), 7.90 (t, *J* = 7.8 Hz, 1H, *pyridine H4*), 7.74 (d, *J* = 8.9 Hz, 2H, *Ar H1 and H7*), 7.57 (dt, *J* = 8.2, 1.5 Hz, 3H, *Ar H10, H3 and H5*), 7.53 (dd, *J* = 8.5, 1.8 Hz, 2H, *Ar H9 and H11*), 7.39 (dd, *J* = 7.7, 4.8 Hz, 1H, *pyridine H6*), 6.12 (s, 2H, *CH_2*). **^{13}C NMR (100 MHz, CD3CN) δ (ppm)**: 154.03, 153.27, 153.14, 149.52, 148.08, 142.19, 142.05, 141.09, 128.47, 125.01, 123.56, 112.54, 111.42, 110.58, 109.51, 106.52, 53.28. **HR-MS (ESI-TOF)** m/z: [M+] Calcd for $C_{25}H_{15}O_2N_2^+$, 375.1134, found 375.1133.

General procedure C for the synthesis of DAOTA compounds **7-8**

In a 10 mL microwave vial, the corresponding ADOTA triangulene (1.00 equiv) is added and dissolved in acetonitrile (0.08 M). Then, the corresponding primary amine (25.0–50.0 equiv) is added gradually. The mixture was heated to 100 °C in a Biotage Initiator+ 2.0 microwave reactor using the standard absorbance level (400 W maximum power). Then, the reaction was cooled at room temperature and the solvent was removed *in vacuo*.

*8-amino-4-(3-cyanophenyl)-4,8-diaza-12-oxatriangulenium tetrafluoroborate (**7**)*

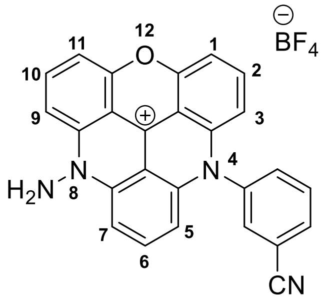

Following the general procedure C, compound **7** was prepared from **1** (50.0 mg, 0.11 mmol, 1.00 equiv) and hydrazine monohydrate (0.26 mL, 50.0 equiv). After 2 h the reaction was completed. The crude product is purified by flash chromatography using alumina and a 0–5% gradient of MeOH in CH_2Cl_2 as the eluent, affording **7** as a violet solid. Yield: 5.00 mg (9 %). **HPLC** (Sunfire C_{18}, 20-95% of A in B, 5 min); t_R = 4.23 min. **LC-MS (m/z)**: 400 $[M+H]^+$. **^{1}H NMR (400 MHz, CD_3CN) δ (ppm)**: 8.18 – 8.07 (m, 4H, *H1, H2, H3 and H4 cyanophenyl*), 8.06 – 8.03 (m, 1H, *Ar H6*), 8.01 (d, *J* = 7.9 Hz, 1H, *Ar H10*), 7.96 – 7.91 (m, 1H, *Ar H2*), 7.87 – 7.78 (m, 4H, *Ar H3, H5, H7 and H9*), 7.30 (dd, *J* = 11.3, 8.1 Hz, 1H, *Ar H11*), 6.56 (dd, *J* = 23.2, 8.5 Hz, 1H, *Ar H1*), 5.47 (s, 2H, *NH_2*). **^{13}C NMR (75 MHz, CD_3CN) δ (ppm)**: 153.38, 153.26, 144.06, 143.46, 142.77, 141.92, 141.07, 140.30, 139.83, 139.27, 138.89, 135.55, 134.84, 134.37, 133.79, 116.71, 111.73, 111.11, 110.36, 109.91, 109.37, 108.47, 108.16, 107.83, 107.18. **HR-MS (ESI-TOF)** m/z: [M+] Calcd for $C_{26}H_{15}ON_4^+$, 399.1246, found 399.1244.

*8-(2-aminoethyl)-4-(3-cyanophenyl)-4,8-diaza-12-oxatriangulenium tetrafluoroborate (**8**)*

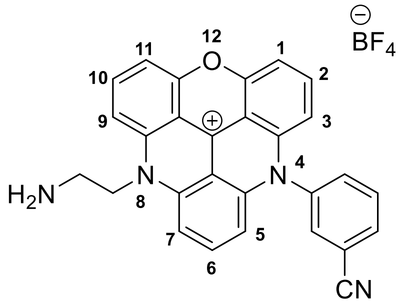

Following the general procedure C, compound **8** was prepared from **1** (50.0 mg, 0.11 mmol, 1.00 equiv) and ethylenediamine (0.18 mL, 25.0 equiv). After 1 h the reaction was completed. The crude product was purified by recrystallization with dichloromethane-diethyl ether, affording **8** as a violet solid. Yield: 47.5 mg (84 %). **HPLC** (Sunfire C_{18}, 2-50% of A in B, 5 min); t_R = 5.18 min. **LC-MS (m/z)**: 428 $[M+H]^+$. **^{1}H NMR (400 MHz, CD_3CN) δ (ppm)**: 8.18 – 8.11 (m, 2H, *Ar H2 and H6*), 8.03 (tt, *J* = 8.0, 4.4 Hz, 2H, *Ar H3 and H5*), 7.94 (t, *J* = 1.9 Hz, 1H, *Ar H10*), 7.85 (q, *J* = 8.6 Hz, 2H, *Ar H1 and H11*), 7.75 (d, *J* = 8.9 Hz, 1H, *H2 cyanophenyl*), 7.61 (d, *J* = 8.8 Hz, 1H, *H4 cyanophenyl*), 7.39 (d, *J* = 8.1 Hz, 1H, *Ar H7*), 7.32 (d, *J* = 8.2 Hz, 1H, *Ar H9*), 6.58 (d, *J* = 8.7 Hz, 1H, *H6 cyanophenyl*), 6.53 (d, *J* = 8.5 Hz, 1H, *H5 cyanophenyl*), 4.59 (t, *J* = 7.1 Hz, 2H, *N_{Ar}-CH_2*), 3.21 (t, *J* = 7.1 Hz, 2H, *$\underline{CH_2}$-NH_2*). **^{13}C NMR (100 MHz, CD_3CN) δ (ppm)**: 153.73, 153.44, 143.20, 142.10, 141.97, 141.86, 141.62, 140.21, 139.83, 139.32, 139.06, 135.60, 134.89, 134.44, 133.88, 125.73, 116.84, 112.21, 111.09, 110.98, 109.90, 109.64, 109.16, 108.23, 107.96, 107.93, 51.48, 39.06. **HR-MS (ESI-TOF)** m/z: [M+] Calcd for $C_{28}H_{19}ON_4^+$, 427.1559, found 427.1557.

Spectroscopy measurements

For spectroscopic studies, at least, two concentrations of each triangulenium dye were prepared in spectroscopy grade solvents. Absorption spectra were collected on a Cary 4000 (Agilent Technologies) spectrophotometer using a 1 cm path length cuvette. Prior to each measurement, a baseline correction was performed using the corresponding pure solvent. Absorbance values were adjusted to an optimal range (0.05–0.2) to minimize inner-filter effects and detector saturation. All spectra were corrected using wavelength-dependent calibration factors provided by the instrument manufacturer. Emission spectra were acquired on a Jasco FP-8300 spectrofluorometer equipped with a 1 cm cuvette. Samples were excited at two different wavelengths, separated by approximately 10 nm to verify spectral consistency and provide independent measurements of quantum yields. Emission scans were collected with 2.5 nm excitation and emission slit widths, starting 10 nm above the excitation wavelength to avoid scattering interferences. For fluorescence quantum yield (Φ_f) determination, the emission spectra were corrected for wavelength-dependent instrument sensitivity and Φ_f were obtained using the relative method by comparing emission/absorption ratios against a reference of known quantum yield (Rhodamine 101 standard in ethanol, $\Phi_{f,reference} = 0.91$)[3] and QY values were calculated using the comparative method, based on the reference dye's known quantum yield. Time-resolved fluorimetry was performed on a PicoQuant FluoTime 200, equipped with a 530 nm pulsed laser (Picoquant, P-FA-530B), working at a repetition rate of 5 MHz, as set by a PDL-800 laser driver (PicoQuant), providing a 200 ns temporal detection window. The instrument response function (IRF) was recorded daily using a Ludox colloidal silica suspension to account for temporal dispersion and ensure accurate lifetime determination. Three independent fluorescence decay traces were collected at λ_{em}^{max} and $\lambda_{em}^{max} \pm 5nm$, for each solution, including at least two different concentrations of each dye in each solvent. Fluorescence decay traces were globally analyzed using PicoQuant EasyTau software, employing an iterative reconvolution procedure. The decays were fitted to mainly to monoexponential functions. In a few cases, biexponential functions were required. In such cases, the intensity-weighted average lifetime was reported in Tables S1-S8. The quality of the fits was assessed based on the χ^2 values and residuals distribution.

DFT calculations

Electronic structure Density Functional Theory (DFT) calculations were performed using the ORCA 6.0.1 package, employing the dispersion-corrected wB97X-D4 functional, with def2-TZVP Ahlrichs basis set and the corresponding auxiliary basis sets were used for all atoms, and taking advantage of the Resolution of Identity approximation for coulomb and HF exchange integrals (RIJCOSX approximation). The SCF calculations were of the spin-polarized type and tight convergence criteria were used. Initial coordinates for geometry optimization were generated on Avogadro Software and pre-optimized with a Molecular Mechanics MMFF94s force field with a steepest descent algorithm with 5000 steps and a 10^{-10} convergence tolerance. To guarantee that the converged structures were energy local minima harmonic frequencies calculations were done to check for the presence of imaginary frequencies. The dispersion correction was employed to model more accurately the non-covalent interactions that may be present intramolecularly. A conductor-like polarizable continuum model (CPCM) for water, toluene and dioxane was included in all calculations to account for solvent effects and to compare for potential differences in molecular orbital distributions arising from non-specific solvent interactions.

Time-Dependent DFT (TD-DFT) calculations were performed employing the same level of theory, with the same basis and CPCM to account for solvent influence over the first ten singlet excited states. Excited state geometry optimization was performed only for the first excited state.

Frontier molecular orbitals representation was done with ChemCraft Software with an isovalue of 0.04. Electrostatic Potential was calculated and plotted with Avogadro Software over the Van der Waals surfaces. Transition molecular orbitals were calculated using the ORCA_plot module for the $S_0 \rightarrow S_1$ transition. Energy of the HOMO molecular orbitals was extracted as obtained from the calculations. But, to avoid energy gap over or underestimation due to functional limitations, the LUMO energy was calculated as the HOMO energy plus the energy obtained from the TD-DFT for the first transitions, as this only involves the HOMO and LUMO orbitals.

The estimated lipophilicity, in terms of logP, and other properties to assess the subcellular localization of the dyes was performed using the algorithms in Molinspiration[4] and SWISSADME.[5-6]

Cell microscopy and image analysis

HeLa cells were cultured using Dulbecco's Modified Eagle's Medium (DMEM, ThermoFisher) complemented with a final concentration of 10% of fetal bovine serum (FBS). To thaw cryopreserved cells, the vial was gently agitated in a 37°C water bath for approximately 2 minutes. After thawing, the vial was promptly removed, and its surface was decontaminated with 70% ethanol. All subsequent steps were performed under strict aseptic conditions. The cell suspension was transferred into a centrifuge tube containing 9 mL of complete culture medium and centrifuged at 400g for 5 minutes. The resulting cell pellet was resuspended in fresh complete culture medium and seeded into an appropriately sized culture flask. Prior to cell seeding, the culture medium had been equilibrated in a CO_2 incubator to stabilize the pH within the optimal range (7.0–7.6) and prevent excessive alkalinity. Finally, the culture was incubated at 37°C in a 95% humidified atmosphere with 5% CO_2 to ensure optimal cell growth and viability.

Fluorescence lifetime imaging (FLIM) was performed using a confocal microscope (Abberior Instruments GmbH, Heidelberg, Germany) equipped with a Multiharp 150 4N time-correlated single-photon counting (TCSPC) module (PicoQuant GmbH, Germany). The system was configured with a UPlanSApo 100× oil-immersion objective (NA 1.4), and the pinhole was set to 1 Airy unit. The samples were excited with a 485 nm pulsed laser, working at 5 MHz for a TCSPC time window of 200 ns with a time resolution of 160 ps/channel. FLIM data acquisition used a 200 ns time window with a 600 µs dwell time per pixel. Colocalization experiments employed two excitation lasers: the 485 nm laser for the triangulenium dye and a 640 nm laser for MitoTracker Deep Red, MT (Sigma Aldrich) mitochondria staining dye. Emission signals were collected using avalanche photodiodes (APDs) with bandpass filters: 605/50 nm for triangulenium and 685/70 nm for MT. For colocalization imaging, the system operated in pixel-step mode, with 10 µs excitation/emission collection per laser pair per pixel, resulting in a total dwell time of 20 µs per pixel. Super-resolution stimulated emission depletion (STED) microscopy was performed in the same instrument, using a 775-nm, pulsed, toroidal, depletion laser.[7] The depletion laser is overlapped spatially and temporally (pulses) with the 485-nm excitation laser. The dwell time for STED imaging was 60 µs per pixel.

In these experiments, cells were treated with 1× PBS prepared by diluting commercial 10× PBS (Sigma-Aldrich, P5493-1L). Prior to staining, cell cultures were washed twice with 200 µL of 1× PBS to eliminate potential background interference from the pigmented medium. Subsequently, 200 µL of triangulenium dye (2 µM in 1× PBS) was added to each well of an 8-well µ-Slide (Ibidi). For colocalization experiments, 25 nM MT was co-applied. Cells were incubated for 30-40 minutes at 37°C in a 5% CO_2 humidified atmosphere, and subsequently placed in the microscope stage for imaging.

Prior to imaging, we performed a prescan of each well to identify regions of interest. Initial imaging was performed on an 80×80 μm^2 area encompassing the target cells, with a spatial resolution of 150 nm/pixel. Subsequently, higher-resolution images of selected sub-regions (ranging from 20 to 50 μm^2) were acquired for final fluorescence lifetime imaging microscopy (FLIM), achieving a spatial resolution between 85 and 63 nm/pixel. Colocalization imaging was conducted at a spatial resolution of 50 nm/pixel. STED imaging was performed with a smaller pixel size (20 nm/pixel) for enhancing the potential sub-diffraction limited patterns.

For colocalization analysis, the intensity images obtained from colocalization experiments were analyzed using Fiji.[8] Initial visual assessment was performed by overlaying the red channel for MT fluorescence and the green channel for the triangulenium dye, so that colocalized areas appeared in yellow. The regions of interest (ROIs) in each channel were selected using an automatic intensity threshold, based on the *moments* algorithm, built-in in Fiji. Then, the Mander's colocalization coefficients were estimated as the fraction of colocalized pixels with respect to the total selected pixels for each channel. Average values from at least 10 different images were obtained for each triangulenium dye derivative. To quantify colocalization we did not use Pearson's coefficient as a suitable criterion, since for some of the dyes the subcellular distribution was not specific on mitochondria.

FLIM images were analyzed using SymphoTime 64 software (PicoQuant), by performing pixelwise fittings of the fluorescence decay traces to a biexponential decay function, using an iterative reconvolution method and an instrument response function (IRF) calculated from the global traces. First, a 2×2 spatial binning and an 8-channel time binning in the TCSPC scale was performed to enhance the individual decay traces. Finally, the FLIM images were reconstructed

by adjusting the intensity (grayscale) and lifetime (color) scales in Fiji (distribution of ImageJ), using the intensity-weighted average lifetime from the biexponential fitting.

Super-resolution structured illumination microscopy (SIM) was performed on a Nikon AX system built upon a Nikon Eclipse Ti2, equipped with multi-line lasers, Nikon D-LEDI multi-line LEDs and Hamamatsu ORCA Flash 4.0 sCMOS monochromatic sensor camera. HeLa cells were fixed upon 1 h incubation with the dye, using *p*-formaldehyde and ProLong™ Diamond antifade mountant medium.

Photophysical properties of compounds 1-8

Table S1. Photophysical properties of ADOTA **1** in different solvents.

**Solvent**	**λ^{max}_{abs} /nm**	**λ^{max}_{em} /nm**	**ε /$M^{-1}cm^{-1}$**	**τ_f /ns** ^{a,b}	**Φ_f** ^{c}
Methanol	536.5	559.5	7350	20.23	0.786
Water	538	557	9059	20.17	0.773
MeCN	538	561	8640	21.6	0.832
Ethanol	531.5	560	8974	20.64[d]	0.701
Isoprop.	537	559	7308	20.97	0.719
Butanol	538	562	7042	20.52	0.760
Pentanol	536	561	4963	20.37[d]	0.695
Dioxane	537.5	564.5	7090	16.17	0.439
Octanol	538	563	7425	20.62[d]	0.743
DMSO	541	568.5	9550	13.62	0.440
Toluene	536.5	568.5	7717	16.48[d]	0.640

[a] The reported τ_f values are the measured lifetime, when monoexponential decays were obtained, or the intensity-weighted average lifetime in multiexponential traces, averaged over the values obtained at the different wavelengths. The complete table of fitting results is included as a supporting data sheet in the supplementary materials of the manuscript. [b] The lifetime error ranges between 0.005 and 0.05 ns. [c] The quantum yield error ranges between 0.003 and 0.014. [d] From biexponential fitting showing a short decay time, whose amplitude is < 20%.

Table S2. Photophysical properties of ADOTA **2** in different solvents.

**Solvent** [a]	**λ^{max}_{abs} /nm**	**λ^{max}_{em} /nm**	**ε /$M^{-1}cm^{-1}$**	**τ_f /ns** [b,c]	**Φ_f** [d]
Methanol	540	567.5	11268	15.57	0.552
Water	541	563	13700	16.58	0.578
MeCN	541.5	569.5	12791	16.01	0.584
Ethanol	540.5	568	13546	15.22	0.554
Isoprop.	540.8	568.5	11013	15.83	0.591
Butanol	542.4	573	10771	15.15	0.565
Pentanol	542.8	573	9284	14.72[e]	0.568
Dioxane	537	572	11453	3.71[f]	0.109
Octanol	543.2	576	10669	12.86[f]	0.495
DMSO	541	577	14668	5.35	0.214

[a] Not soluble in toluene. [b] The reported τ_f values are the measured lifetime, when monoexponential decays were obtained, or the intensity-weighted average lifetime in multiexponential traces, averaged over the values obtained at the different wavelengths. The complete table of fitting results is included as a supporting data sheet in the supplementary materials of the manuscript. [c] The lifetime error ranges between 0.005 and 0.05 ns. [d] The quantum yield error ranges between 0.007 and 0.030. [e] From biexponential fitting showing a short decay time, whose amplitude is < 20%. [f] From biexponential fitting showing a short decay time, whose amplitude is > 20%.

Table S3. Photophysical properties of ADOTA **3** in different solvents.

Solvent	λ^{max}_{abs} /nm	λ^{max}_{em} /nm	ε /M^{-1}cm^{-1}	τ_f /ns [b,c]	Φ_f [d]
Methanol	536.7	560.5	7361	20.98	0.788
Water	537.5	558	8322	19.50	0.744
MeCN	538.5	562.5	8863	21.93	0.835
Ethanol	536.5	561.8	8905	20.91	0.808
Isoprop.	536.9	560	7170	21.10	0.843
Butanol	538.9	562	7045	20.66	0.825
Pentanol	539.4	562.9	4187	20.60	0.846
Dioxane	537	567.5	8043	17.30[e]	0.647
Octanol	540.5	564.3	6545	20.47	0.620
DMSO	539	570.8	8254.5	14.34	0.565
Toluene	538.2	571	7316	19.71[a]	0.663

[a] Scattering due to certain insolubility. [b] The reported τ_f values are the measured lifetime, when monoexponential decays were obtained, or the intensity-weighted average lifetime in multiexponential traces, averaged over the values obtained at the different wavelengths. The complete table of fitting results is included as a supporting data sheet in the supplementary materials of the manuscript. [c] The lifetime error ranges between 0.007 and 0.010 ns. [d] The quantum yield error ranges between 0.003 and 0.030. [e] From biexponential fitting showing a short decay time, whose amplitude is < 20%.

Table S4. Photophysical properties of ADOTA **4** in different solvents.

**Solvent** [a]	λ^{max}_{abs} **/nm**	λ^{max}_{em} **/nm**	**ε /M**$^{-1}$**cm**$^{-1}$	τ_f **/ns** [b,c]	Φ_f [d]
Methanol	534.6	555.4	2387	21.64	0.856
Water	535	552.5	4472	20.58	0.603
MeCN	535.5	556.5	3945	22.37	0.803
Ethanol	535.5	556.8	4551	21.62	0.656
Isoprop.	535.4	557.3	2466	21.60	0.809
Butanol	537.4	558	2204	21.41	0.896
Pentanol	537.6	558	2021	21.31	0.809
Dioxane	538.5	564	2861	18.59[e]	0.395
Octanol	540	561.8	2310	21.21	0.744
DMSO	537	564.3	2784	17.58	0.610

[a] Not soluble in toluene. [b] The reported τ_f values are the measured lifetime, when monoexponential decays were obtained, or the intensity-weighted average lifetime in multiexponential traces, averaged over the values obtained at the different wavelengths. The complete table of fitting results is included as a supporting data sheet in the supplementary materials of the manuscript. [c] The lifetime error ranges between 0.007 and 0.24 ns. [d] The quantum yield error ranges between 0.002 and 0.050. [e] From biexponential fitting showing a short decay time, whose amplitude is > 20%.

Table S5. Photophysical properties of ADOTA **5** in different solvents.

Solvent	λ^{max}_{abs} /nm	λ^{max}_{em} /nm	ε /$M^{-1}cm^{-1}$	τ_f /ns [a,b]	Φ_f [c]
Methanol	534.5	555.5	2220	21.88	0.230
Water	534.5	553	2060	20.57	0.230
MeCN	536	557	1890	22.49	0.240
Ethanol	534.5	557	1810	21.76[d]	0.270
Isoprop.	535	557.5	1920	21.55	0.240
Butanol	537	558.5	1770	21.34	0.240
Pentanol	537	559.5	2180	21.16[d]	0.230
Dioxane	540.5	564	1920	16.14[d]	0.040
Octanol	539.5	562	2120	20.58[d]	0.190
DMSO	536	563.5	1740	17.5	0.270
Toluene	549	567.5	1510	16.21[d]	0.080

[a] The reported τ_f values are the measured lifetime, when monoexponential decays were obtained, or the intensity-weighted average lifetime in multiexponential traces, averaged over the values obtained at the different wavelengths. The complete table of fitting results is included as a supporting data sheet in the supplementary materials of the manuscript. [b] The lifetime error ranges between 0.01 and 0.12 ns. [c] The quantum yield error ranges between 0.01 and 0.06. [d] From biexponential fitting showing a short decay time, whose amplitude is < 20%.

Table S6. Photophysical properties of ADOTA **6** in different solvents.

Solvent	λ^{max}_{abs} /nm	λ^{max}_{em} /nm	ε /M^{-1}cm^{-1}	τ_f /ns [a,b]	Φ_f [c]
Methanol	536.2	559.2	9086	19.66[d]	0.639
Water	536.5	555	9024	19.45	0.625
MeCN	538.5	560.5	8317	21.60	0.708
Ethanol	537.5	561.5	8564	19.72	0.627
Isoprop.	537.8	562	8964	19.62[d]	0.658
Butanol	539.6	562	8945	19.48[d]	0.717
Pentanol	539.7	562.4	7086	19.54[d]	0.704
Dioxane	542	567	5974	16.72[e]	0.537
Octanol	542.2	564	7795	19.55[d]	0.687
DMSO	539	567.5	8867	16.82	0.538
Toluene	553.8	568.5	7623	18.24[e]	0.659

[a] The reported τ_f values are the measured lifetime, when monoexponential decays were obtained, or the intensity-weighted average lifetime in multiexponential traces, averaged over the values obtained at the different wavelengths. The complete table of fitting results is included as a supporting data sheet in the supplementary materials of the manuscript. [b] The lifetime error ranges between 0.004 and 0.13 ns. [c] The quantum yield error ranges between 0.005 and 0.05. [d] From biexponential fitting showing a short decay time, whose amplitude is < 20%. [e] From biexponential fitting showing a short decay time, whose amplitude is > 20%.

Table S7. Photophysical properties of DAOTA **7** in different solvents.

Solvent	λ^{max}_{abs} /nm	λ^{max}_{em} /nm	ε /$M^{-1}cm^{-1}$	τ_f /ns [a,b]	Φ_f [c]
Methanol	558.5	596	8223	13.63	0.395
Water	555.5	594	8592	11.51[e]	0.312
MeCN	557	597	8994	15.60	0.483
Ethanol	560.5	598.5	11409	13.85[e]	0.412
Isoprop.	563	597.5	10722	13.94[e]	0.417
Butanol	563	599	10790	13.70[e]	0.419
Pentanol	562.5	599	10575	13.23[d]	0.417
Dioxane	564	607.5	7446	9.95[e]	0.325
Octanol	564.5	600.5	11455	12.50[d]	0.402
DMSO	561	610	9117	10.79[e]	0.412
Toluene	564	608	7159	9.51[f]	0.287

[a] The reported τ_f values are the measured lifetime, when monoexponential decays were obtained, or the intensity-weighted average lifetime in multiexponential traces, averaged over the values obtained at the different wavelengths. The complete table of fitting results is included as a supporting data sheet in the supplementary materials of the manuscript. [b] The lifetime error ranges between 0.004 and 0.03 ns. [c] The quantum yield error ranges between 0.002 and 0.07. [d] From biexponential fitting showing a short decay time, whose amplitude is < 20%. [e] From biexponential fitting showing a short decay time, whose amplitude is > 20%. [f] From triexponential fitting.

Table S8. Photophysical properties of DAOTA **8** in different solvents.

**Solvent**	**λ^{max}_{abs} /nm**	**λ^{max}_{em} /nm**	**ε /$M^{-1}cm^{-1}$**	**τ_f /ns** [a,b]	**Φ_f** [c]
Methanol	555	586.5	6700	14.65[e]	0.353
Water	552.5	582	7129	16.78	0.494
MeCN	558.5	594	5045	14.95[e]	0.350
Ethanol	555.5	589.5	7267	11.00[f]	0.278
Isoprop.	558.5	588.5	6733	11.70[e]	0.251
Butanol	559	589	6779	16.58[e]	0.295
Pentanol	559.5	591	7000	12.90[f]	0.292
Dioxane	560.5	594	5992	16.56[d]	0.402
Octanol	561	591	6864	13.90[e]	0.312
DMSO	563	594	7171	14.40[e]	0.279
Toluene	541.5	596.5	5027	14.54[e]	0.122

[a] The reported τ_f values are the measured lifetime, when monoexponential decays were obtained, or the intensity-weighted average lifetime in multiexponential traces, averaged over the values obtained at the different wavelengths. The complete table of fitting results is included as a supporting data sheet in the supplementary materials of the manuscript. [b] The lifetime error ranges between 0.004 and 0.03 ns. [c] The quantum yield error ranges between 0.003 and 0.02. [d] From biexponential fitting showing a short decay time, whose amplitude is <20%. [e] From biexponential fitting showing a short decay time, whose amplitude is >20%. [f] From triexponential fitting.

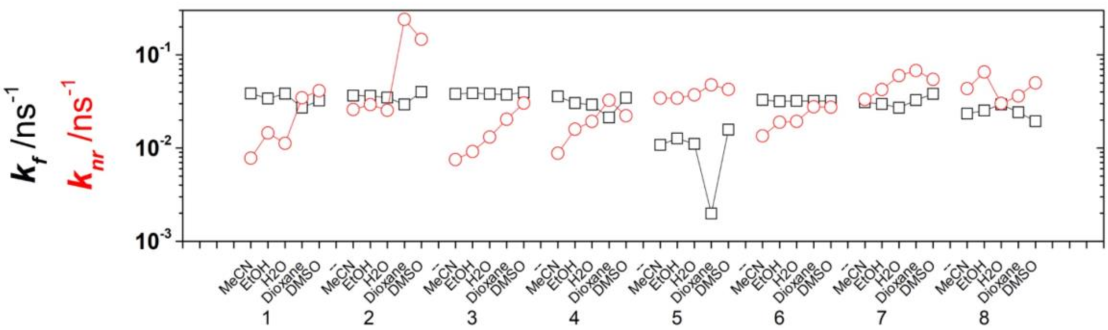

Figure S1. Representative k_f (black symbols) and k_{nr} (red symbols) values of dyes **1**-**8** in selected solvents. The radiative, k_f and nonradiative, k_{nr}, rates demonstrated mostly invariable values for k_f ((34±4)×10^6 s^{-1} for most ADOTAs and (28±5)×10^6 s^{-1} for DAOTAs). A particular case was ADOTA **5** that exhibited lower Φ_f values and k_f rates in the range of (11±5)× 10^6 s^{-1}. Interestingly, k_{nr} rates exhibited dependence with the solvent, increasing its value in more apolar solvents.

Solvatochromism analysis

To get more insights into the solvent effects over the photophysical properties of the dyes, we decided to perform an in-depth study of the solvatochromic behavior of the dyes. Such study was performed using the formalism established by J. Catalán, who set four solvent scales for general solvent effects (solvent dipolarity, SdP, and solvent polarizability, SP) and specific solvent effects (solvent acidity, SA, and solvent basicity, SB).[24] The solvent effect on the photophysical property (y) is extracted by performing a multilinear regression to the equation:

$$y=y_0+a_{SA}\cdot SA+b_{SB}\cdot SB+c_{SP}\cdot SP+d_{SdP}\cdot SdP \qquad \text{(S1)}$$

where a_{SA}, b_{SB}, c_{SP} and d_{SdP} are adjustable parameters for each one of the solvent scales. The fittings are performed considering initially all four solvent scales, then the properties that are not relevant are discarded based on very low values of the coefficients and very large associated errors in the fitting. Removing 1, 2, or even 3 of the solvent scales provided new values for the remaining coefficients. The statistical significance of the fittings with reduced parameters was judged by the regression coefficient (r^2), the χ^2 value, and the F-test.[3]

In our study, we performed the multivariate correlations for the absorption and emission energies ($\bar{\nu}_{abs}$ and $\bar{\nu}_{em}$), the lifetime τ_f, and the radiative and nonradiative rates (k_f and k_{nr}), extracting which of the solvent scales has more effect over the photophysical properties. Figure S2 shows the main results of the solvatochromism analysis for all the studied dyes. The fact that the triangulene moiety is charged and the very long photoluminescence lifetime values are the main reasons behind the solvatochromic dependency on solvent polarizability observed for most of the properties. In all cases, the emission energy is also regulated by the solvent acidity. Increasing acidity causes less stabilization of the excited state (positive values of the SA coefficient for the correlation of $\bar{\nu}_{em}$ values) leading to blue shifts. Since this is a common feature of all the dyes tested, the specific interaction must be related to the triangulenium moiety, which contains the –O– and –N– basic bridges. Solvent acidity interacting with these centers would reduce the donation of electrons to the core, decreasing the stabilization of the excited state.

In all ADOTAs modified with pyridin moieties (**3**-**6**), the solvent polarizability is the main parameter that contributes to the stability of the excited state, leading to red shifts in both absorption and emission, and correlated with faster decay times. This is confirmed through the very good correlation of $\bar{\nu}_{abs}$, $\bar{\nu}_{em}$, and τ_f with the Bayliss function[9] $f(n) = (n^2–1)/(2\cdot n^2+1)$ (where n is the solvent refractive index). Importantly, the HOMO and LUMO molecular orbitals (Figure S3) only showed involvement of the pyridin nitrogen when the relative atom position was *meta*- (dyes **3** and **4**), but not in dyes **5** and **6**, in which solvent dipolarity and basicity also contribute to blue shifts in absorption. This difference is consistent with the resonant forms of the pyridine ring. In all these pyridine-modified ADOTAs, the solvent features have very little influence over k_f rates, while k_{nr} increases with polarizability.

In DAOTA **8**, TD-DFT calculations demonstrated that the C-α of the primary amine group carries electron density in the HOMO orbital, leading to PET towards the triangulene core upon excitation (Figure 2 in main text and Figure S3). Moreover, this amine group carries a notable negative charge. Hence, solvent acidity stabilizes the ground state, leading to blue shifts in both absorption and emission. In contrast, the primary amine group in DAOTA **7** barely holds any negative charge. The solvent dipolarity stabilizes the ground state, leading to absorption blue shifts, whereas electronic polarizability mainly stabilizes the excited state, leading to emission red shifts and faster decays. An effect of solvent acidity or basicity on the photoluminescence τ_f values was found for dyes in which partial PET quenching occurs, namely ADOTA **2** and DAOTAs **7** and **8**. This suggests that specific proton transfer interactions can modulate the efficiency of the PET process.

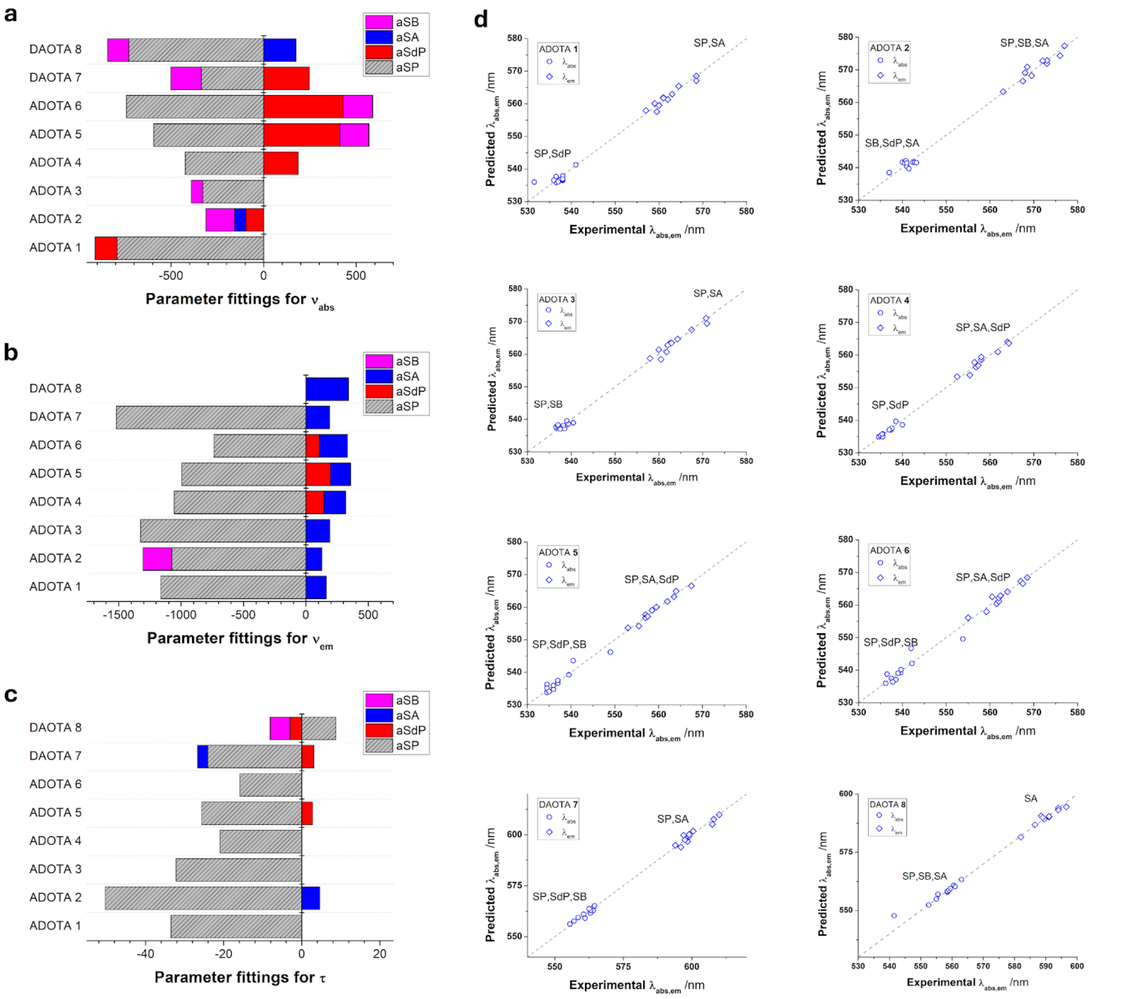

Figure S2. Plots of the weight of the coefficients of the solvents scales, Sdp (red), Sp (gray), SA (blue), and SB (pink), in the correlations of $\overline{\upsilon}_{abs}$ (**a**), $\overline{\upsilon}_{em}$ (**b**), and τ_f (**c**) for dyes **1**-**8**. **d**) Experimental and predicted λ_{abs} (circles) and λ_{em} (diamonds) of dyes **1**-**8** in different solvents, as obtained from the solvatochromic analysis. The solvent scale parameters employed in the estimation of the predicted values are those indicated in each case.

Molecular orbitals of triangulenium dyes 1-8

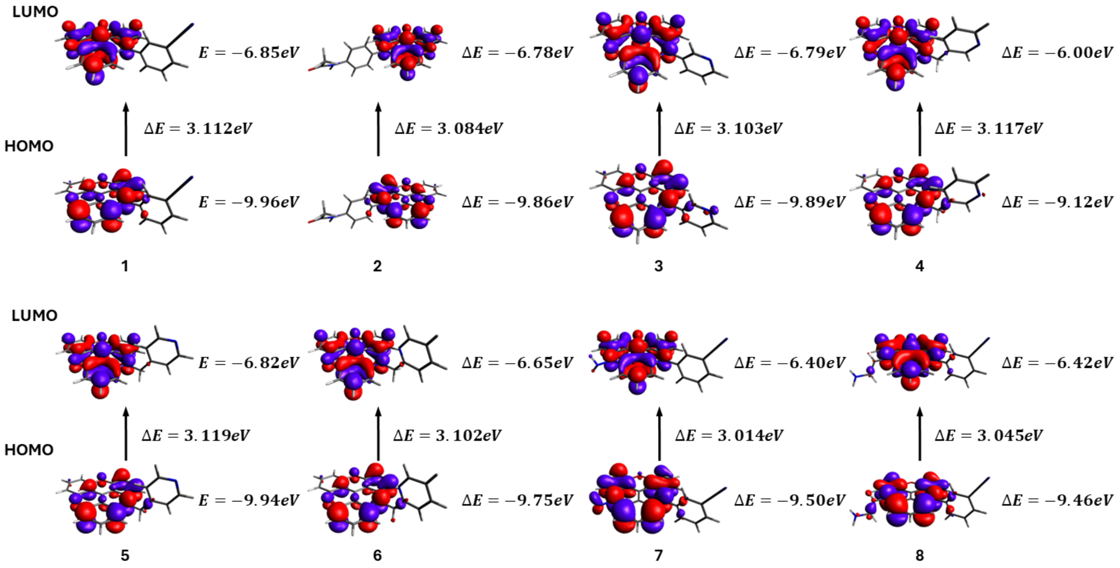

Figure S3. Canonical representation of the frontier orbitals (HOMO and LUMO) for the 8 dyes and their corresponding energy gap.

Lipophilicity of dyes 1-8

Quantitative structure-activity relationship (QSAR) models have been successfully applied to predict, among other properties, subcellular localization of drugs and fluorophores.[10-12] Predicting properties based on molecular fragments allows setting rules of thumb for subcellular delivery. One of the most important parameters is lipophilicity, quantified in terms of logP (partition coefficient octanol:water). Mitochondrial localization is fostered for lipophilic cations, with LogP values ranging from 0 to 4. All our ADOTA and DAOTA dyes fulfill these features. The fact that the dyes are cationic and the main moiety is largely planar also facilitates interaction with DNA. As described in the main text, many of these dyes are also found interacting with nuclear DNA.

Table S9. Estimated logP values for dyes **1-8** using different algorithms.

Compd.	Molinspiration miLogP 2.2 [4]	SWISSADME iLogP [5-6]
**1**	3.58	3.08
**2**	2.86	2.88
**3**	2.78	2.64
**4**	2.72	2.62
**5**	2.67	2.62
**6**	2.79	2.63
**7**	2.93	2.39
**8**	2.35	2.45

Cytotoxicity of dyes 1-8

MRC-5 cell line (human lung fibroblast) was grown in monolayer at 37 °C, 5% CO2 in DMEM medium (1 g/L glucose) supplemented with 100 U/mL penicillin, 100 mg/mL streptomycin, 10% hiFBS and 2 mM L-glutamine. HeLa (human cervical adenocarcinoma) cells were grown in monolayers at 37 °C, 5% CO2 in DMEM medium (4.5 g/L glucose) supplemented with 10% hiFBS, 100 U/mL penicillin, 100 mg/mL streptomycin and 2 mM L-glutamine. Non-essential aminoacids were also added to HeLa culture.

Cytotoxicity of the triangulenium derivatives in MRC-5 and HeLa cells was determined by the alamarBlue® assay (ThermoFisher scientific).[13] Briefly, 1 mM stock solutions of the compounds in DMSO were prepared and the final DMSO percentage in each well was adjusted to be less than 1%. $5{\times}10^3$ cells in 100 µL per well were seeded for 24 h before compound addition to allow the cells to attach and stabilize. They were subsequently incubated at 37 °C, 5% CO2 in 96-wells plates alone (controls) or in the presence of increasing concentration of compounds for 72 h. Then, 20 µL of resazurin (1× solution, 0.11 mg/mL) were then added to each well and the cells were incubated for another 4 hours at 37 °C. Finally, cells were lysed by adding 50 µL of SDS 3% per well. Then, the plate was incubated at 37 °C for an extra hour and fluorescence intensity was recorded with an Infinite F200 plate reader (Tecan Austria, GmbH, Grödig, Austria), using 550/590 nm as the excitation/ emission wavelengths. Negligible interference from the triangulenium dyes luminescence was detected while fitting IC50 curves. Results are expressed as the concentration of compound that reduces cell growth by 50% versus untreated control cells (IC50). Data are presented as the average of at least three independent measurements all conducted in triplicate conditions.

Table S10. EC_{50} values of dyes **1-8** in HeLa and MRC5 cells.

Compd.	EC_{50} (HeLa) /µM	EC_{50} (MRC5) /µM
**1**	5.8 ± 0.3	2.1 ± 0.5
**2**	6.8 ± 2.4	2.0 ± 0.1
**3**	4.4 ± 0.9	1.3 ± 0.8
**4**	10.2 ± 2.1	9.7 ± 0.8
**5**	6.7 ± 0.7	>10
**6**	8.6 ± 0.1	2.8 ± 0.5
**7**	3.6 ± 0.1	0.4 ± 0.2
**8**	8.6 ± 1.3	1.3 ± 0.4

Supporting cell microscopy figures

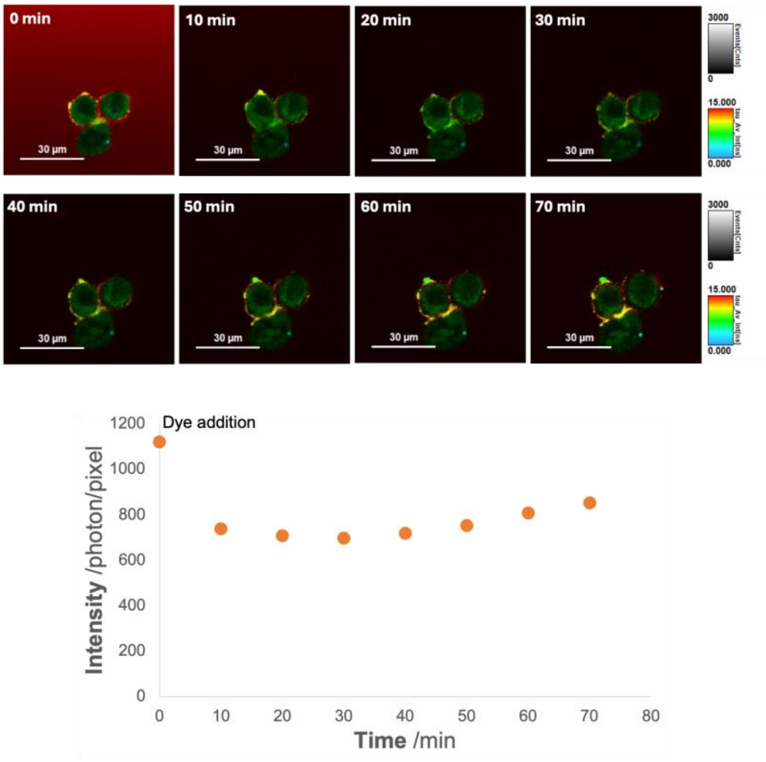

Figure S4. FLIM images of dye **2** (λ_{ex} = 485 nm, detection bandpass at 605/50 nm) in HeLa cells to study uptake kinetics. The dye was added at time 0, and images were acquired every 10 min. The intensity quantification plot is also shown, exhibiting the photostability of the dye.

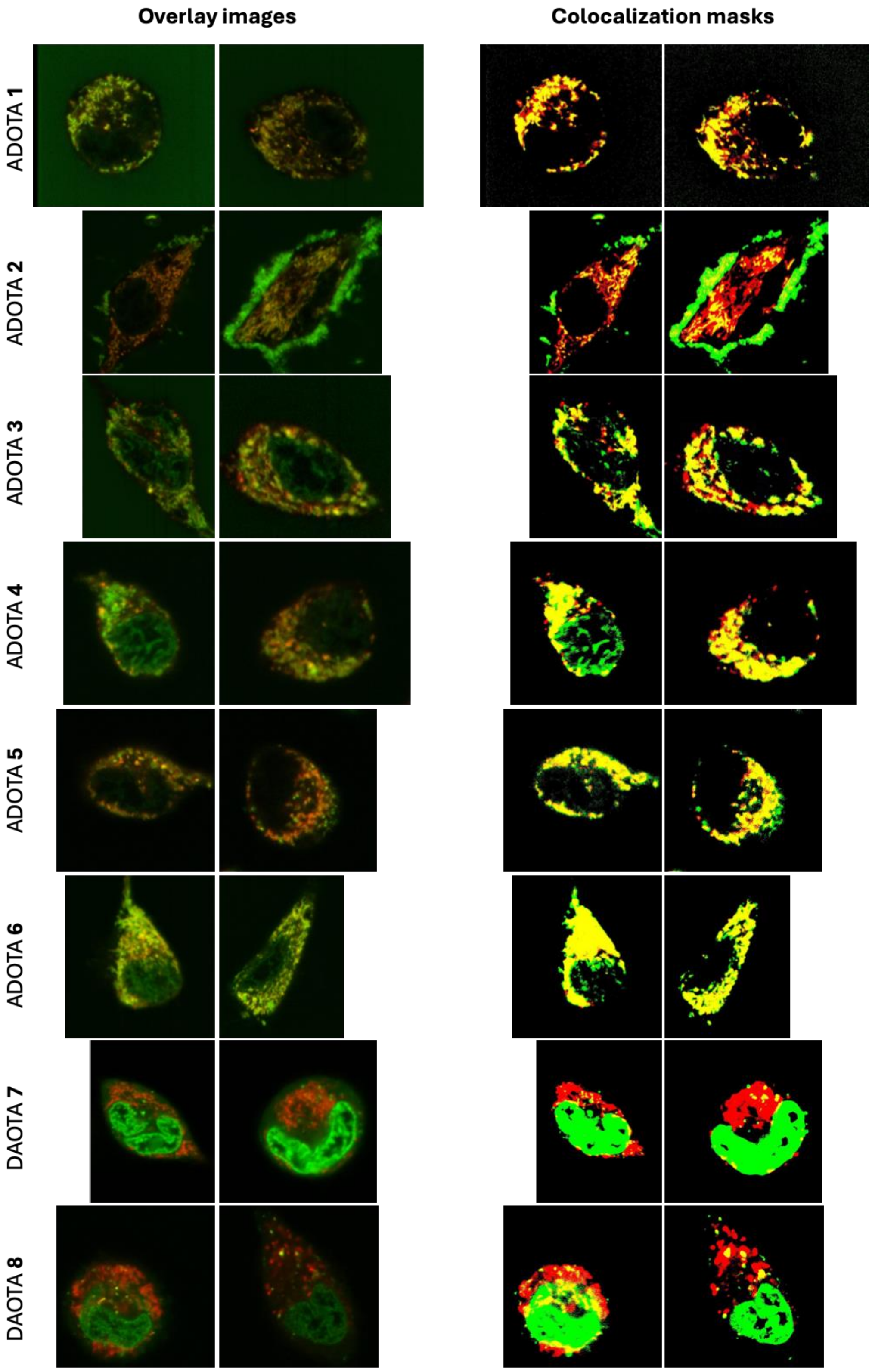

Figure S5. Additional colocalization imaging of dyes **1**-**8** (green channel, λ_{ex} = 485 nm, detection bandpass at 605/50 nm) and MT (red channel, λ_{ex} = 485 nm, detection bandpass at 685/70 nm) in HeLa cells. The right column of images shows the binary masks in both channels and colocalized pixels in yellow for the quantification of colocalized areas.

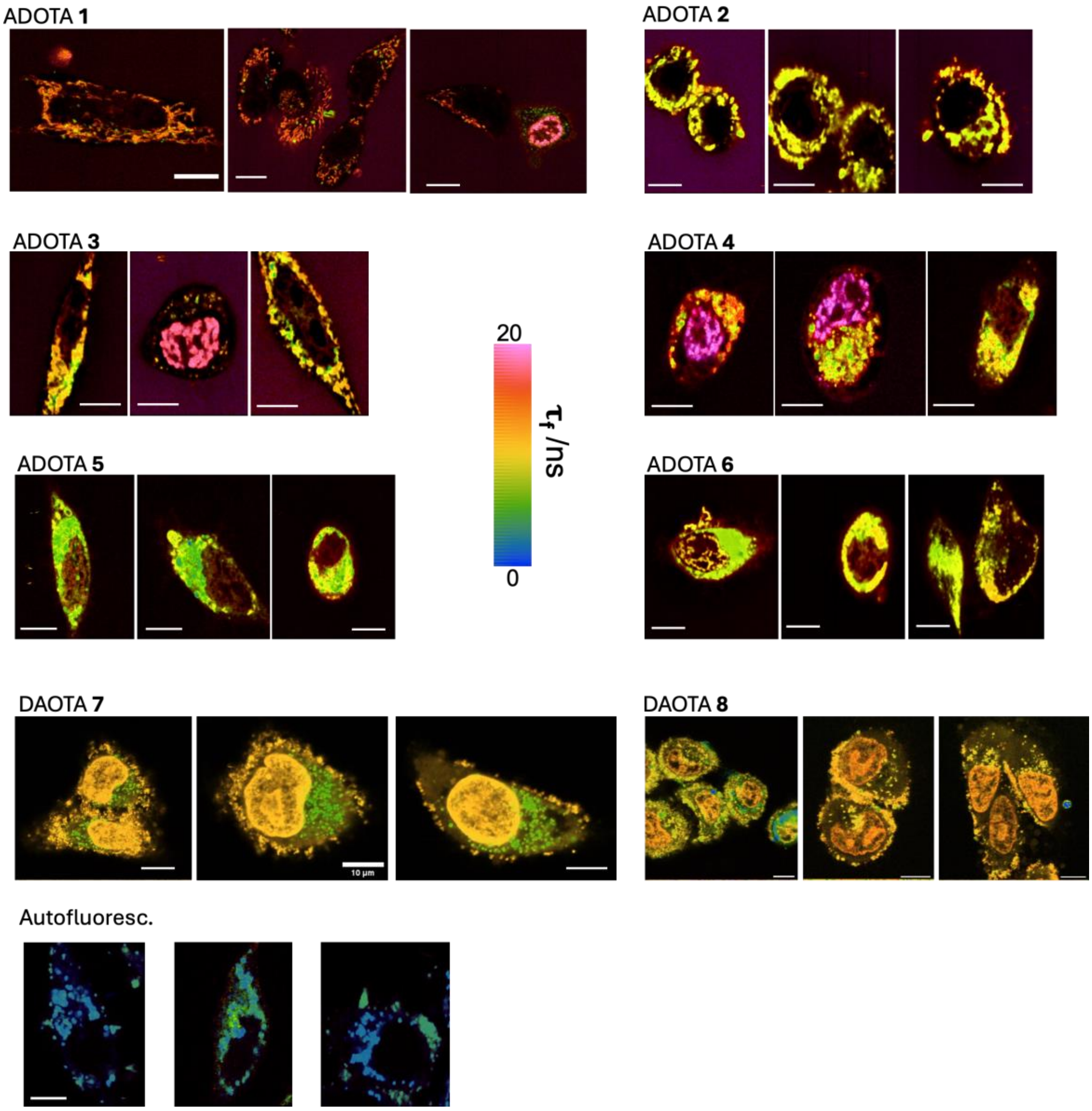

Figure S6. Additional representative FLIM images of ADOTAs **1**-**6** and DAOTAs **7**-**8** (2 μM) in HeLa cells (λ_{ex} = 485 nm; detection band 580-630 nm). The pseudocolor scale represents the τ_f values. Scale bars represent 10 μm. Representative images of autofluorescence are also shown, although their intensity is enhanced 7-fold to illustrate the low τ_f values.

Interaction of 1-8 with CT-DNA

Calf thymus DNA (CT-DNA, Sigma Aldrich) was resuspended in Tris 10 mM, KCl 100 mM, pH 7.4 buffer. Fluorescence lifetime, τ_f, were obtained from solutions of dyes **1-8** (1.2 μM) in the same buffer and in the presence of 1.2 μM (in base-pairs) of CT-DNA. Figure S6 shows the τ_f values of the dyes in the absence and the presence of CT-DNA. Three different decay traces were obtained at λ_{max}, λ_{max} + 5 nm, and λ_{max} – 5 nm. Global fittings to biexponential functions were performed, and the intensity-weighted average values are reported as τ_f.

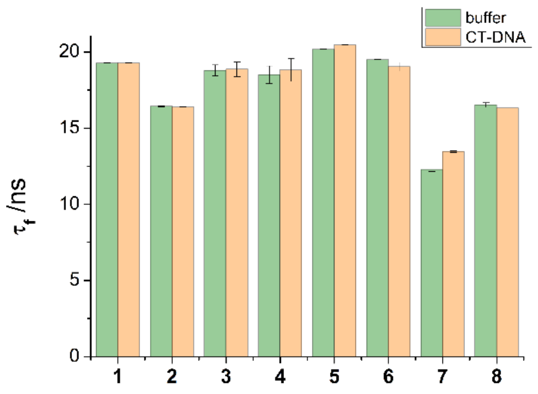

Figure S7. Fluorescence lifetime τ_f of ADOTAs **1**-**6** and DAOTAs **7** and **8** (1.2 μM) in the absence and the presence of CT-DNA (1.2 μM bp). λ_{ex} = 530 nm.

Super-resolution microscopy images

We wanted to test whether some of the dyes were susceptible for state-of-the-art super-resolution microscopy techniques. We tested DAOTA **7**, a candidate that showed excellent nucleus internalization and high brightness, in structured illumination microscopy (SIM). SIM images (Figure S7) exhibited excellent resolution levels for chromatin and nuclei staining.

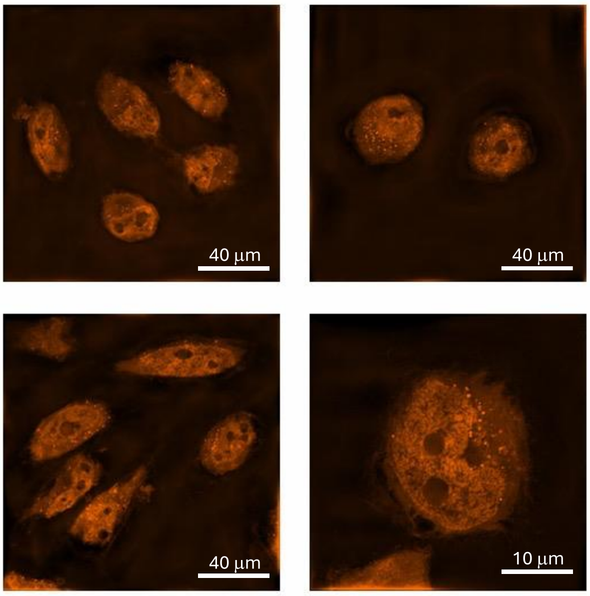

Figure S8. Representative super-resolution SIM images of dye **7** (2 µM) in fixed HeLa cells (λ_{ex} = 561 nm; detection at 615 nm).

We also tested the dyes with the largest mitochondrial accumulation (ADOTAs **3**-**6**, see Table 1 in the main text) in super-resolution STED microscopy (Figure S8). STED microscopy enhances resolution levels by reducing the excitation probe volume through overlapping a toroidal-shaped, red, high power depletion laser. We performed measurements seeking to optimize the depletion laser power and the relative delay between the excitation laser pulse and the depletion laser pulse. However, resolution levels did not improve under STED mode in any of the cases. This is due to the fact that overlapping between the energy of the depletion laser and the emission spectra of the dyes is not large, causing a poor depletability upon excitation.

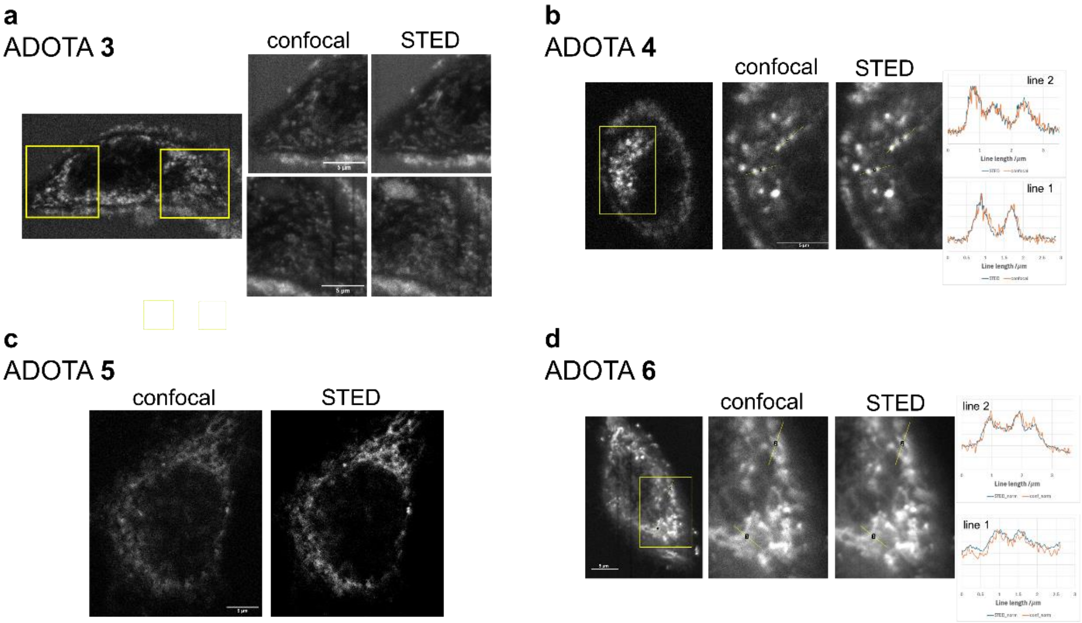

Figure S9. Comparative images in confocal and STED mode of ADOTA dyes **3**–**6** (2 µM) in HeLa cells (λ_{ex} = 485 nm; depletion laser at 775 nm; detection at 615 nm).

Annex 1. NMR spectra

*NMR of acridinium salts (**10**-**15**)*

10-(3-cyanophenyl)-9-(2,6-dimethoxyphenyl)-1,8-dimethoxy-acridin-10-ium tetrafluoroborate (**10**):

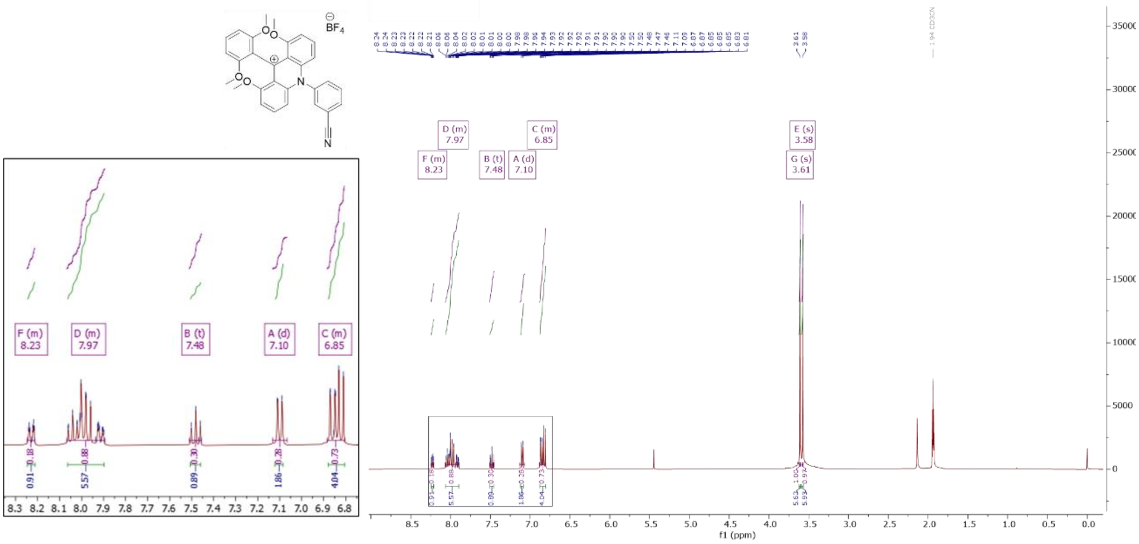

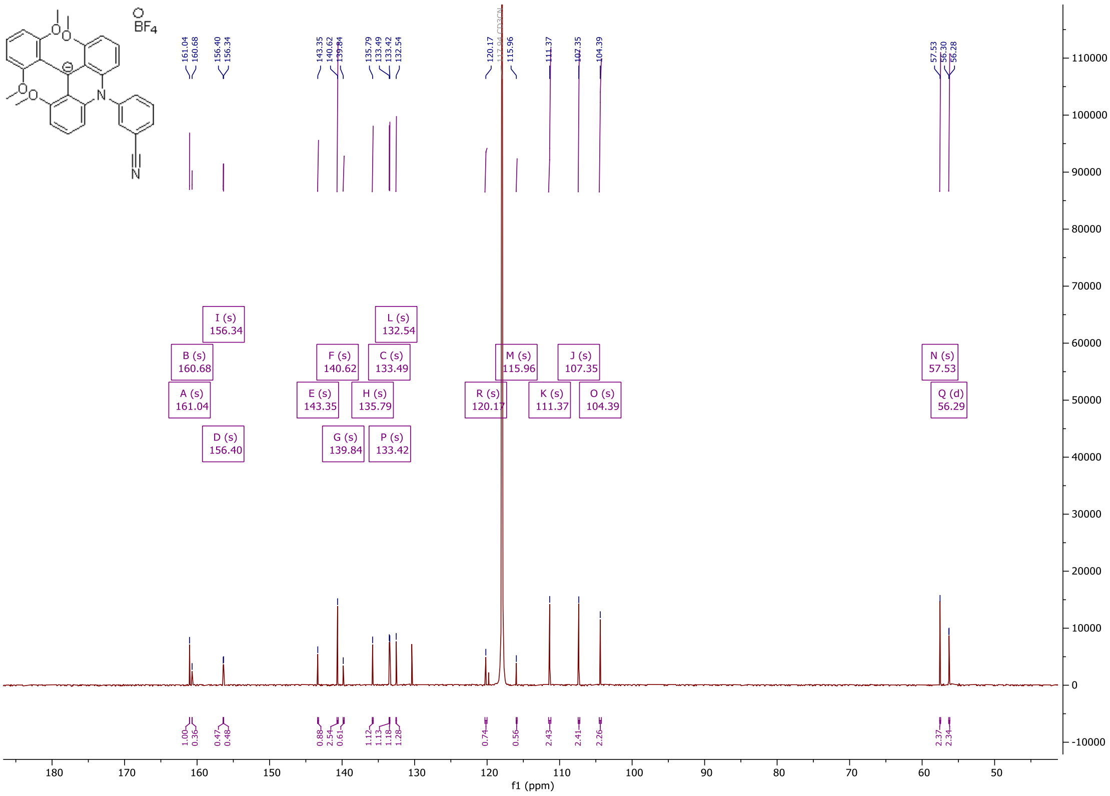

10-(4-acetamidophenyl)-9-(2,6-dimethoxyphenyl)-1,8-dimethoxy-acridin-10-ium tetrafluoroborate (**11**)

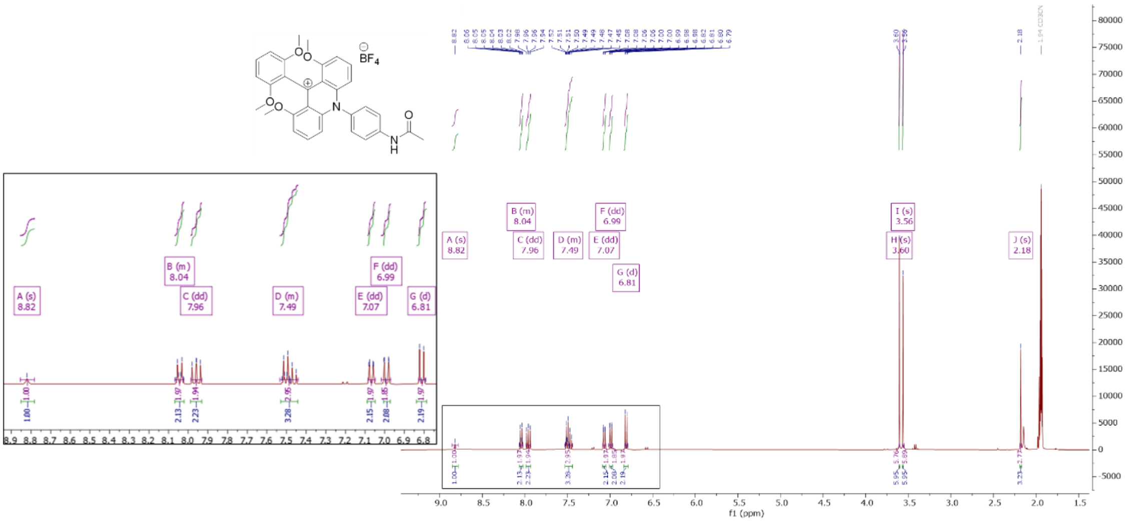

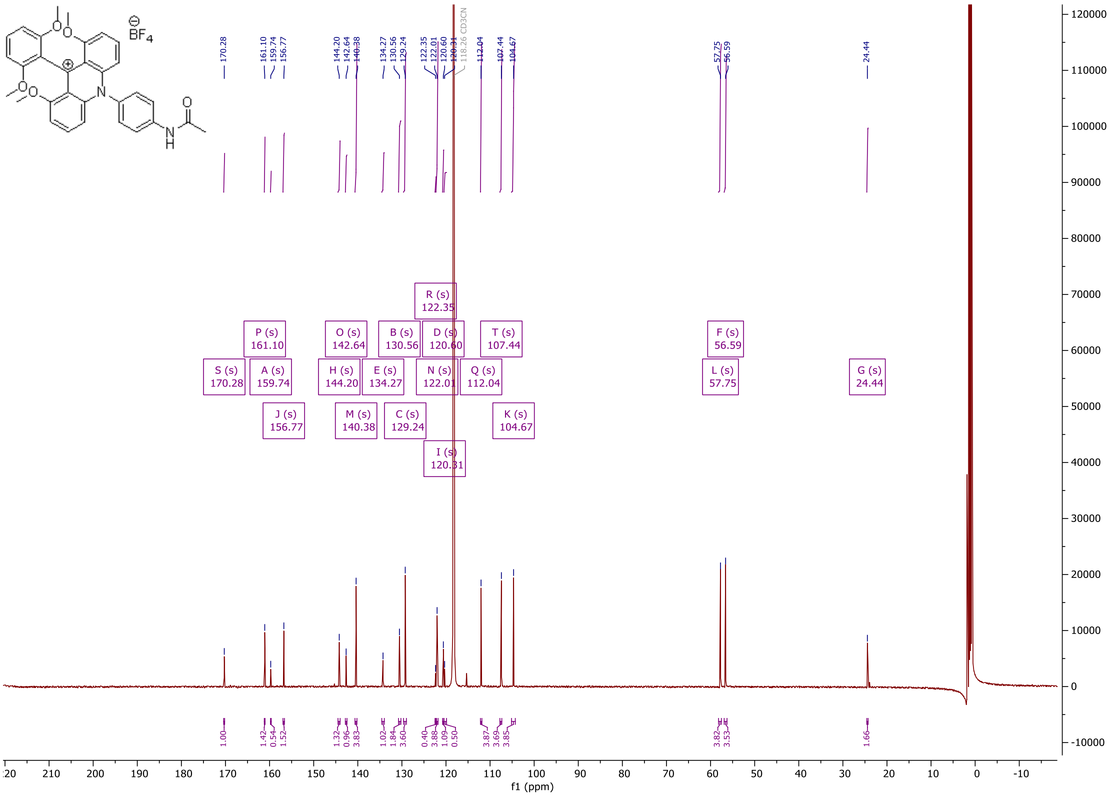

9-(2,6-dimethoxyphenyl)-1,8-dimethoxy-10-(pyridin-3-yl)-acridin-10-ium tetrafluoroborate (**12**)

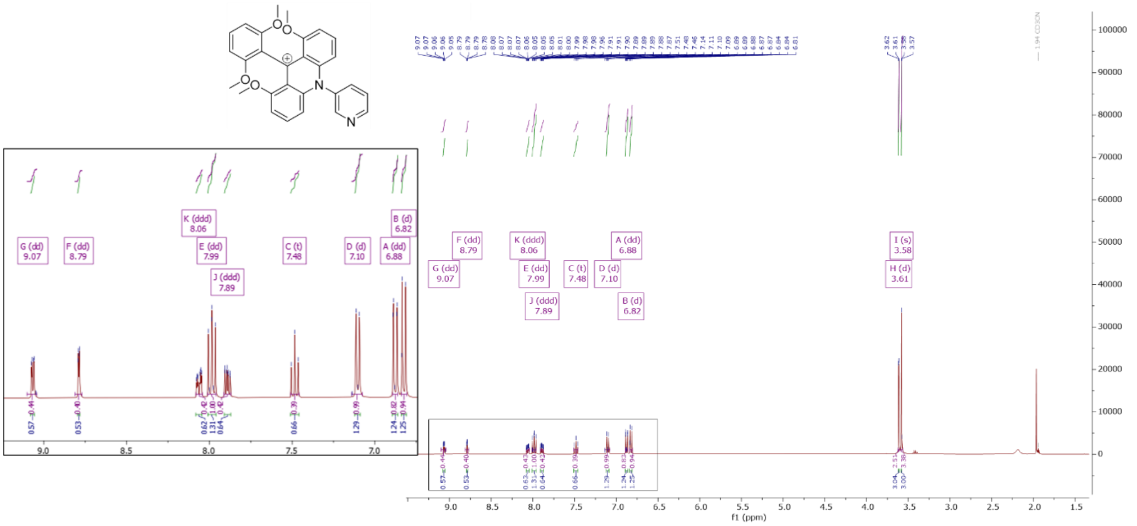

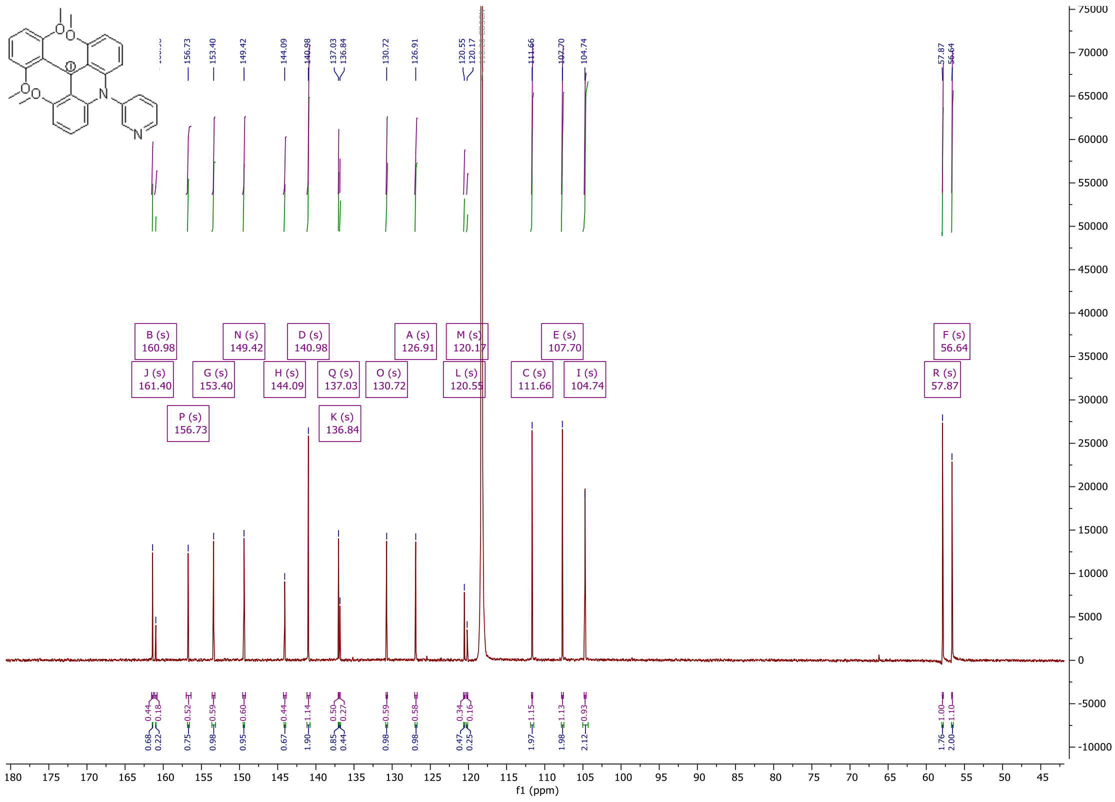

9-(2,6-dimethoxyphenyl)-1,8-dimethoxy-10-(pyridin-3-ylmethyl)-acridin-10-ium tetrafluoroborate (**13**)

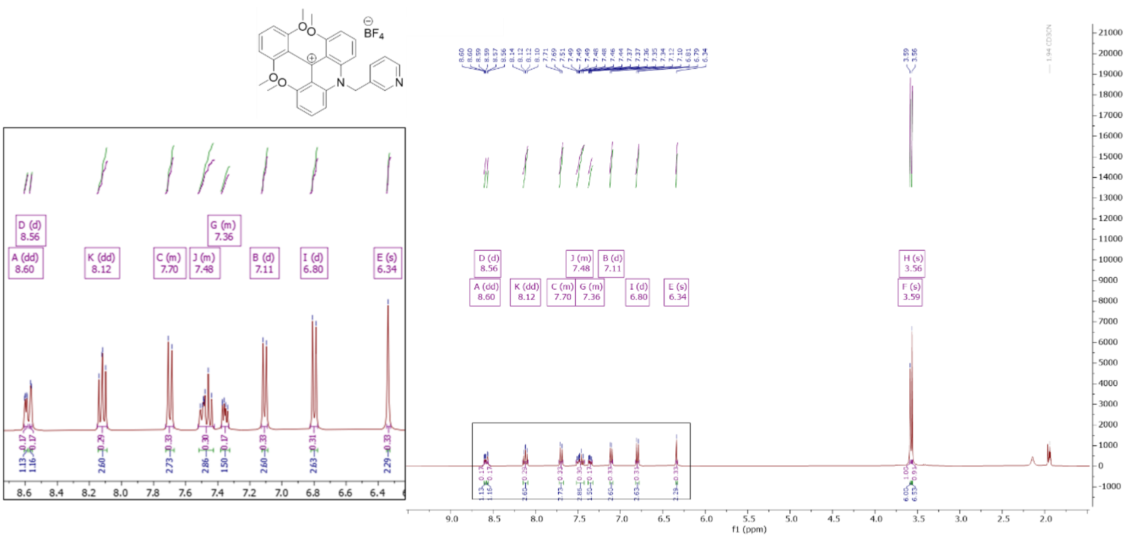

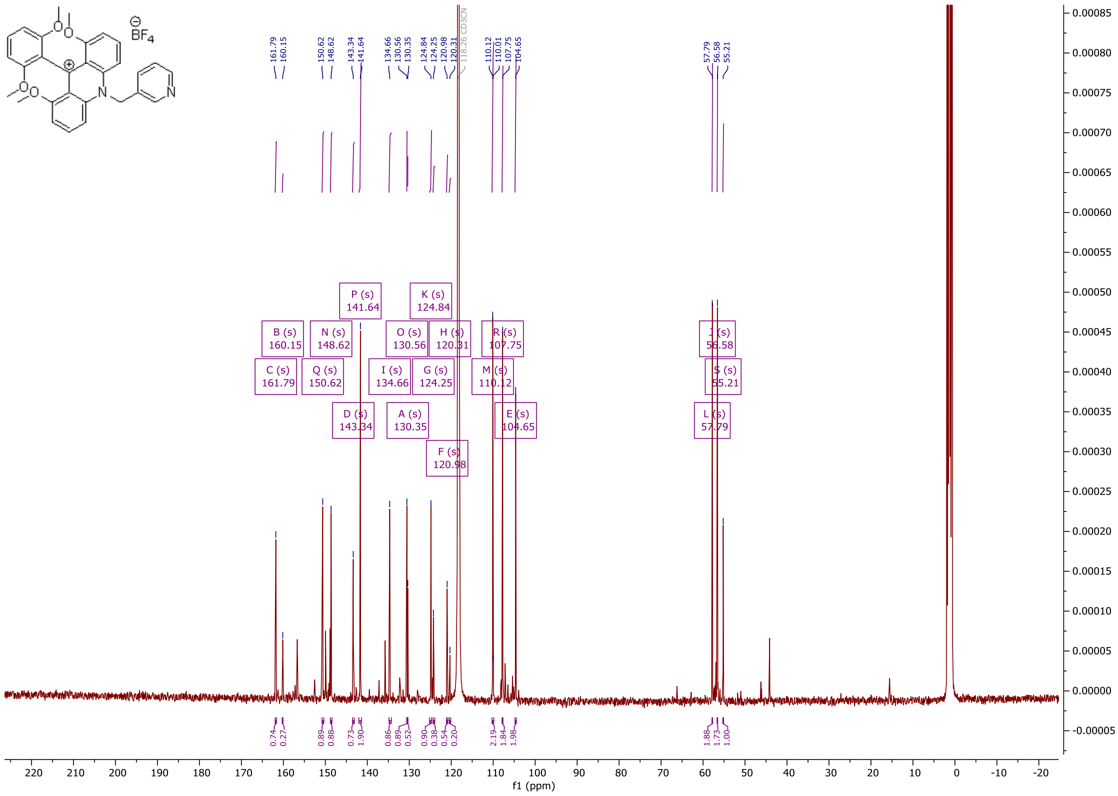

9-(2,6-dimethoxyphenyl)-1,8-dimethoxy-10-(pyridin-4-ylmethyl)-acridin-10-ium tetrafluoroborate (**14**)

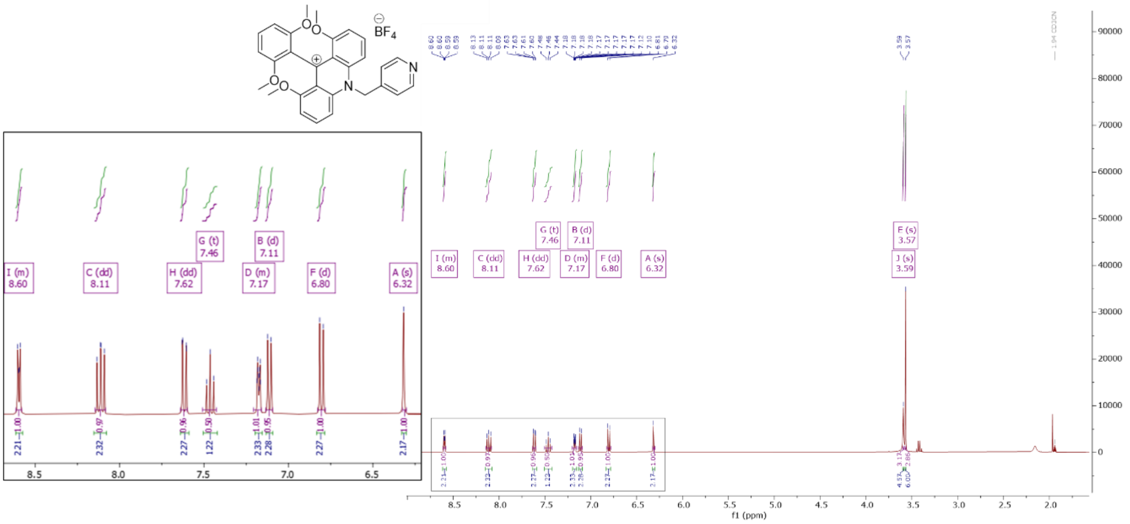

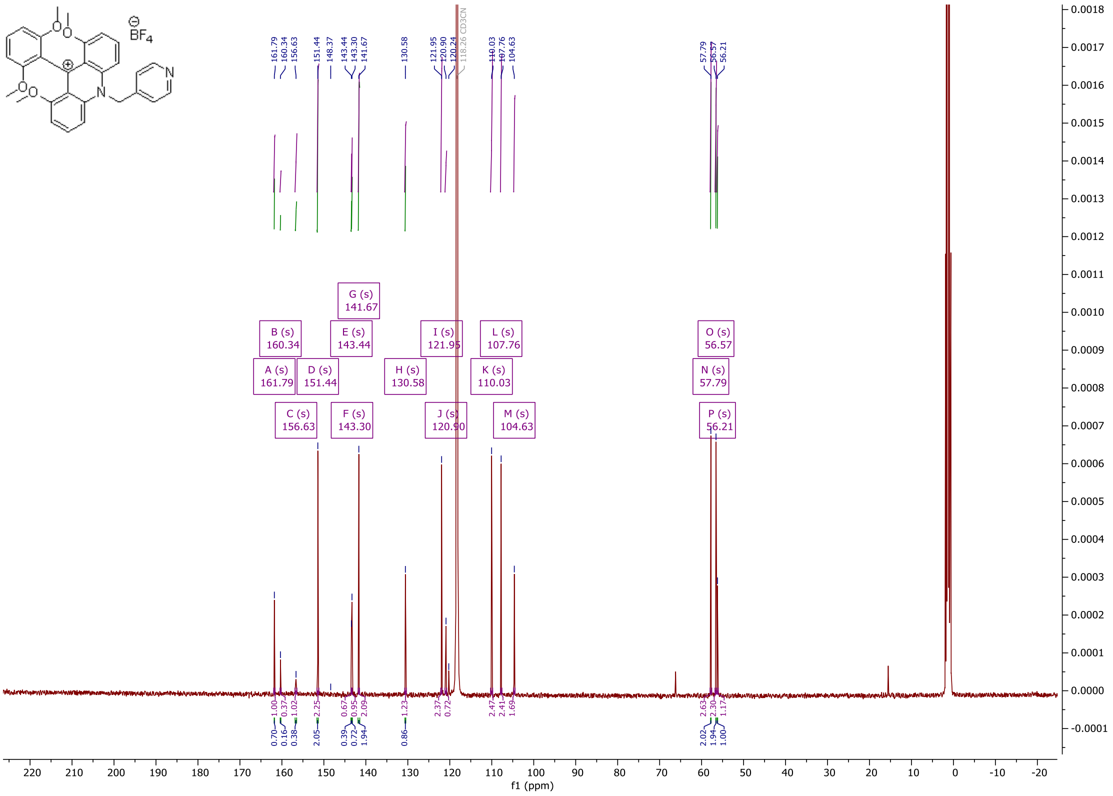

9-(2,6-dimethoxyphenyl)-1,8-dimethoxy-10-(pyridin-2-ylmethyl)-acridin-10-ium tetrafluoroborate (**15**)

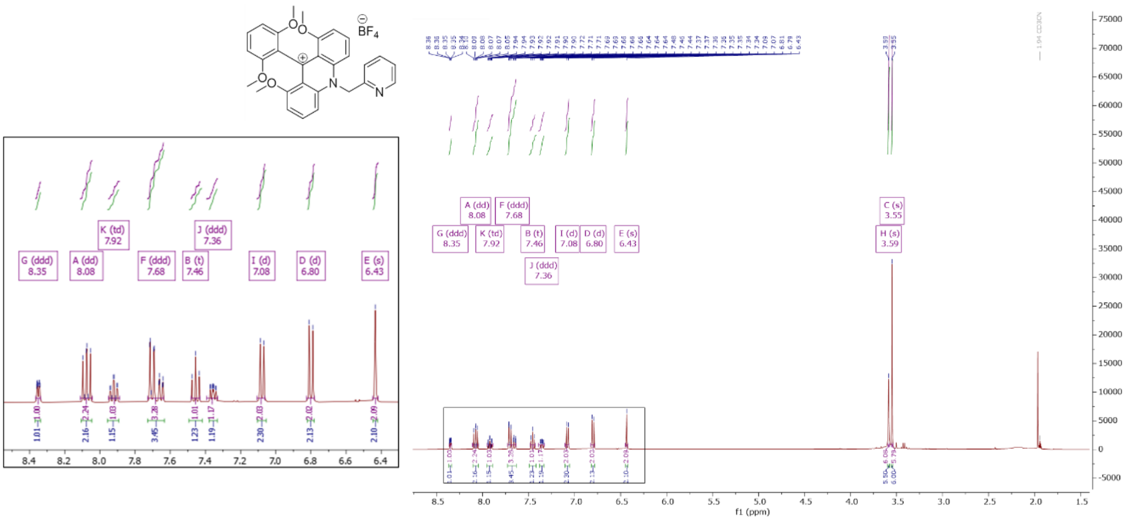

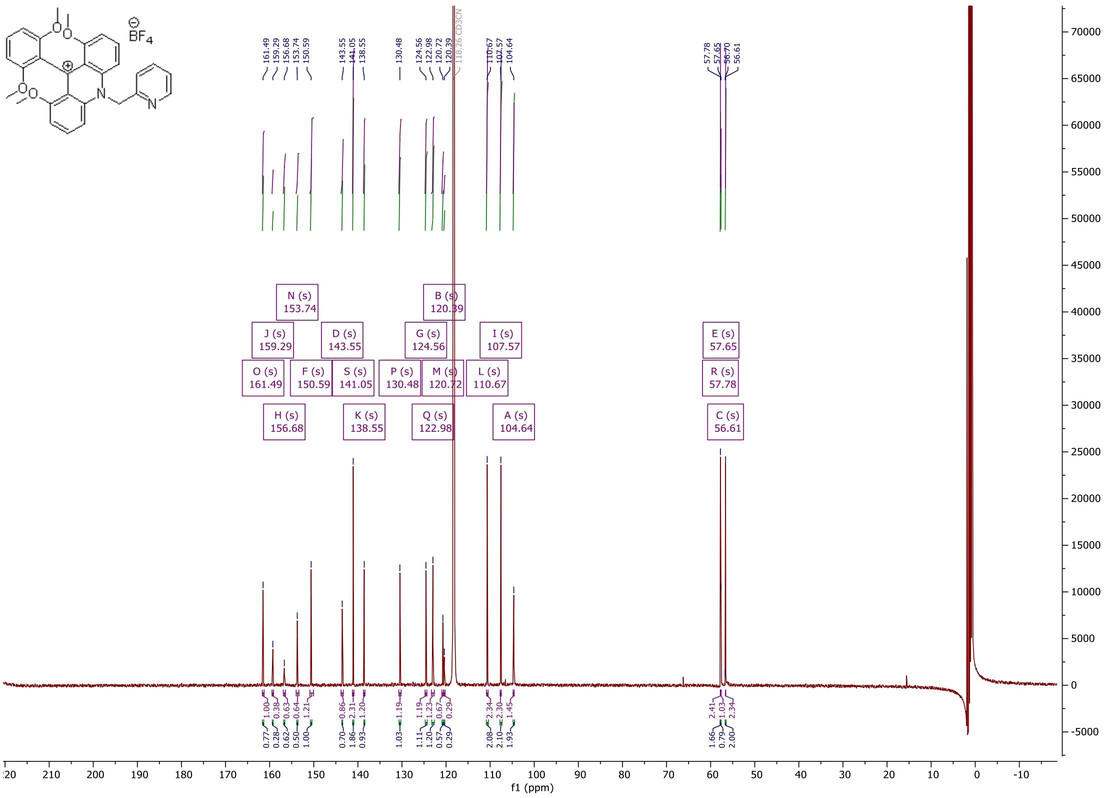

*NMR of ADOTA compounds (**1-6**)*

4-(3-cyanophenyl)-4-aza-8,12-dioxatriangulenium tetrafluoroborate (**1**)

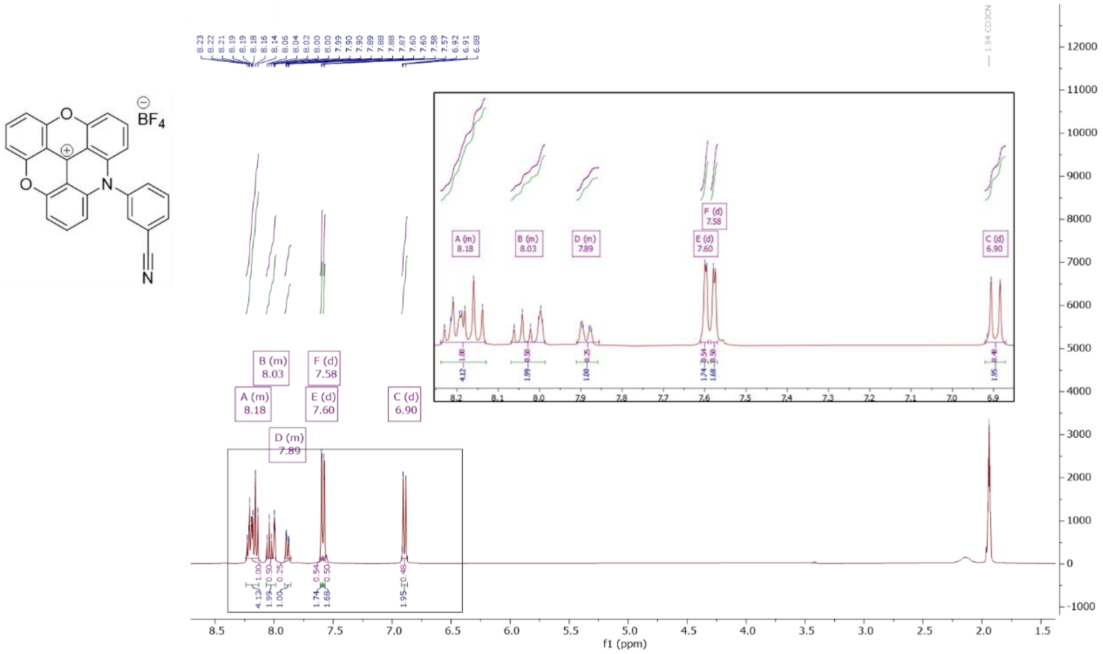

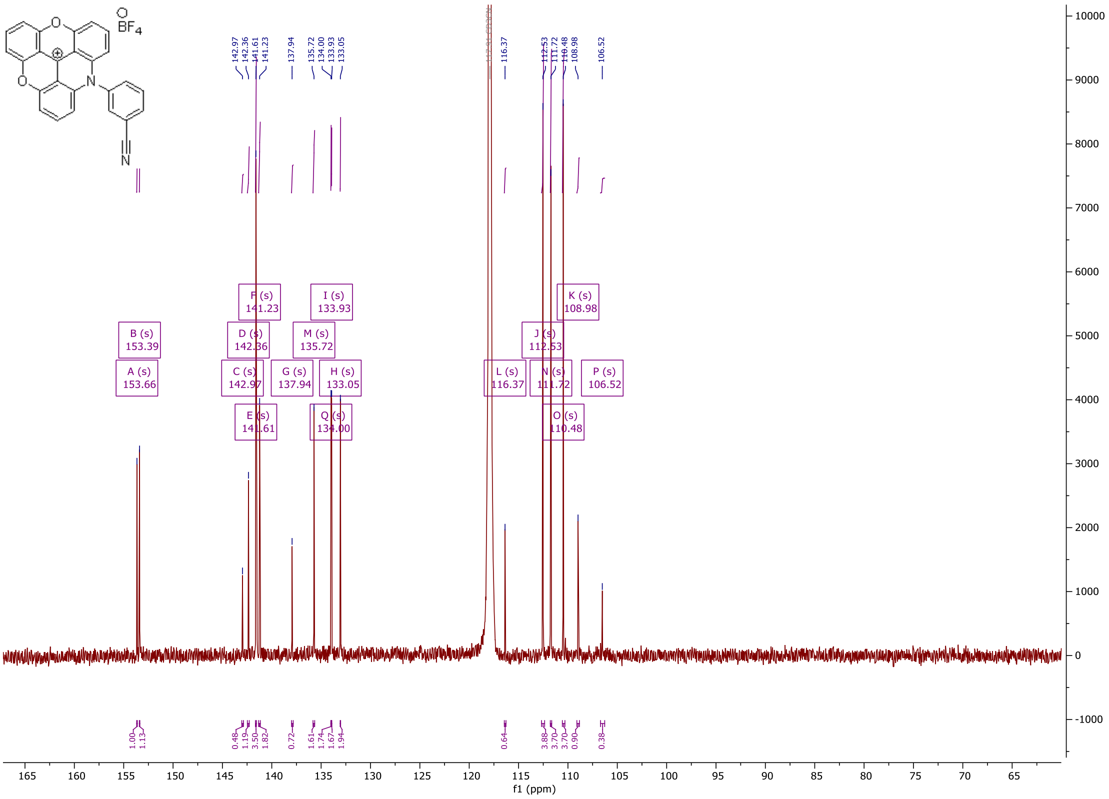

4-(4-acetamidophenyl)-4-aza-8,12-dioxatriangulenium tetrafluoroborate (**2**)

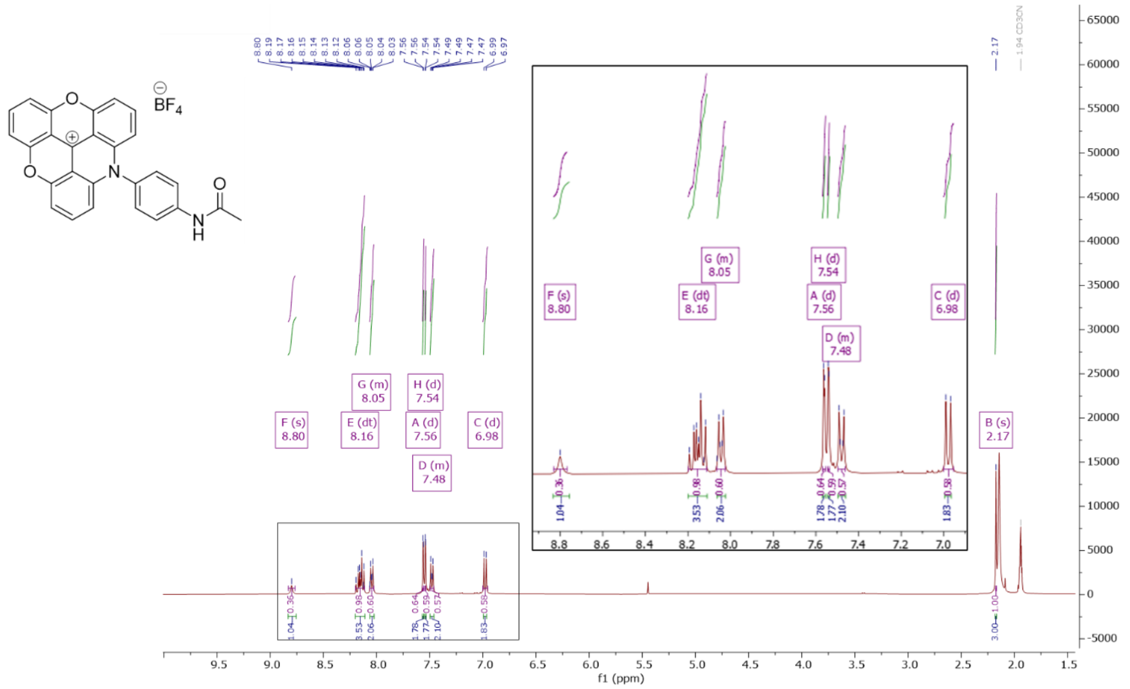

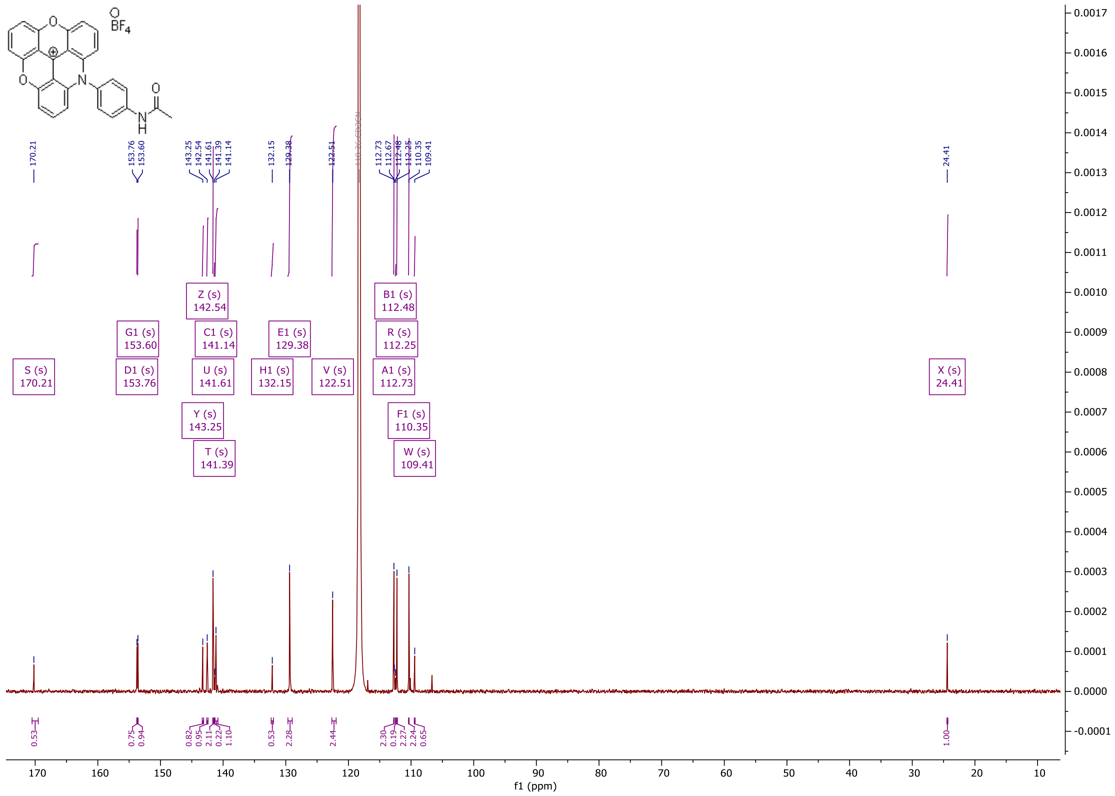

4-(pyridin-3-yl)-4-aza-8,12-dioxatriangulenium tetrafluoroborate (**3**)

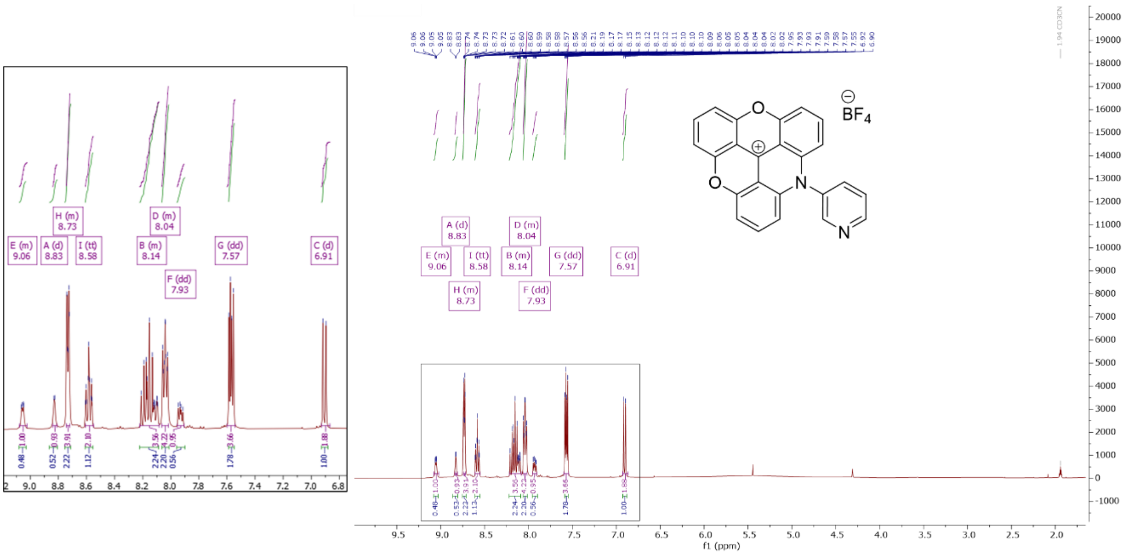

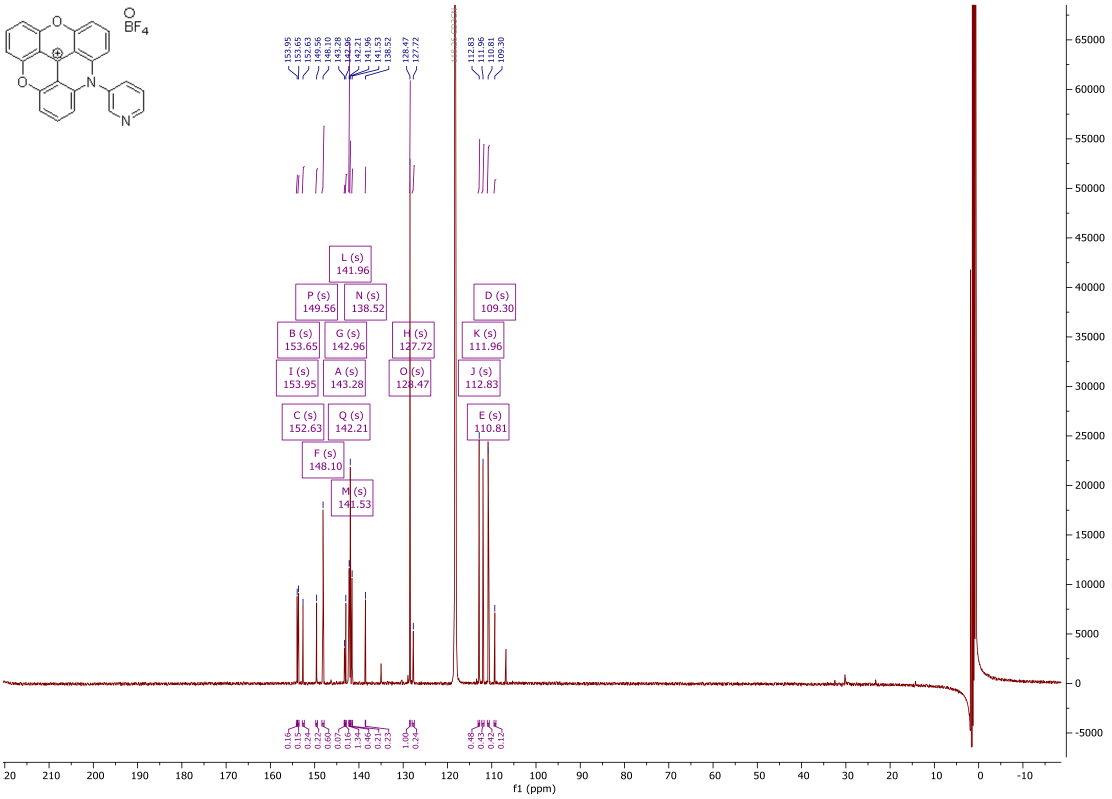

4-(pyridin-3-ylmethyl)-4-aza-8,12-dioxatriangulenium tetrafluoroborate (**4**)

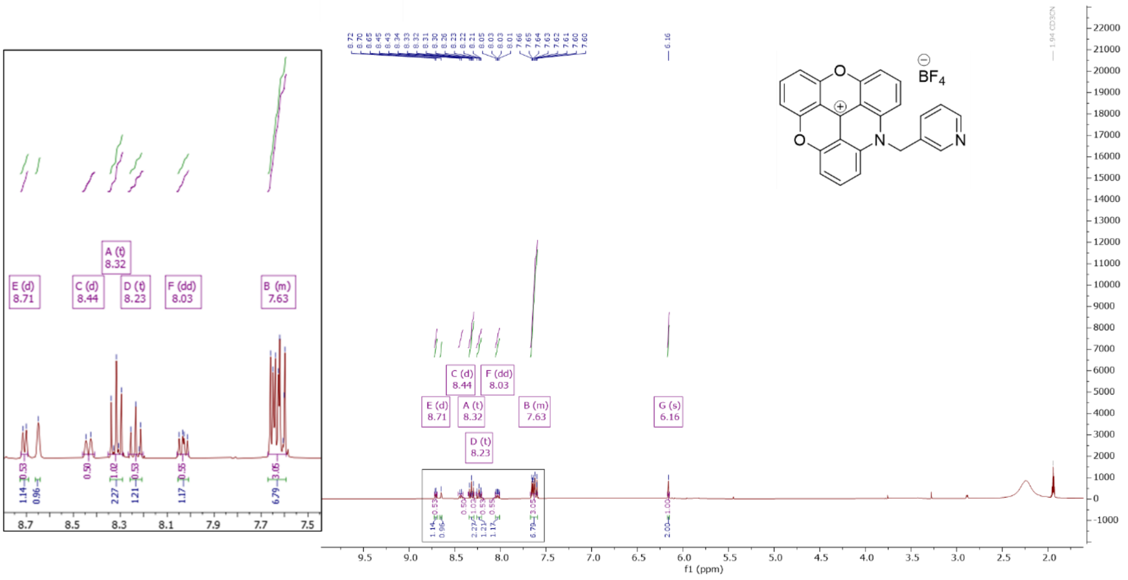

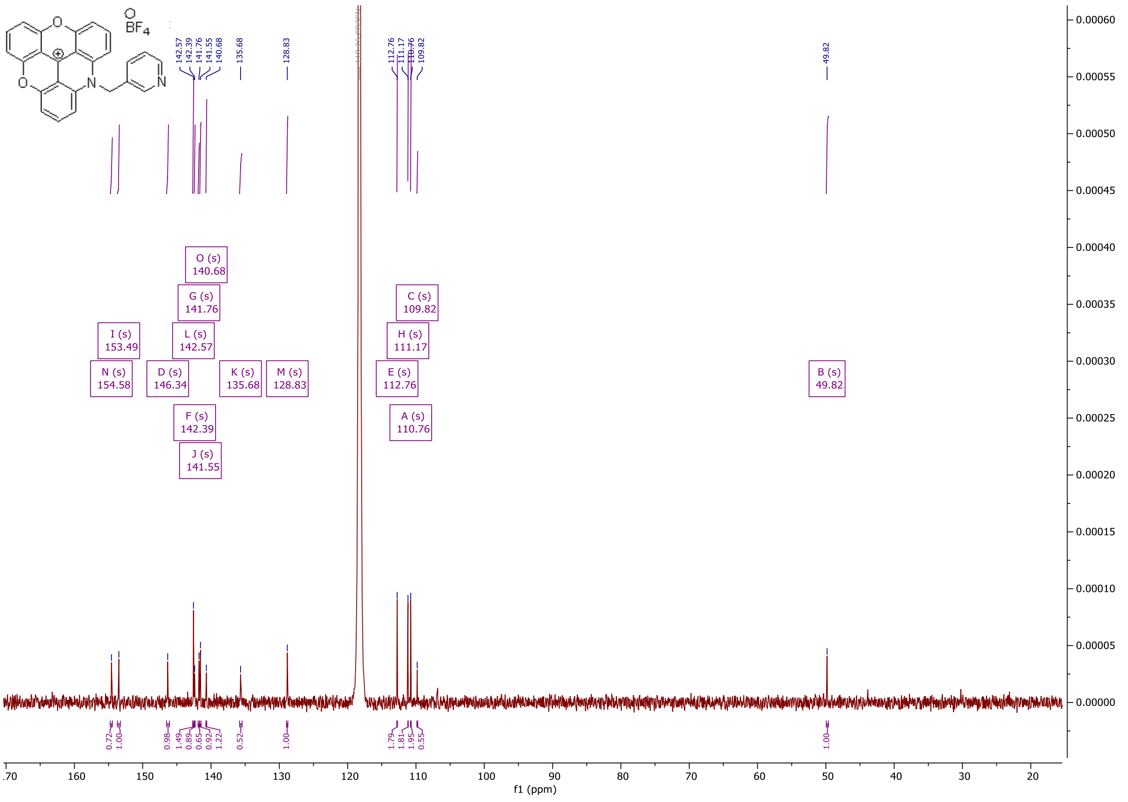

4-(pyridin-4-ylmethyl)-4-aza-8,12-dioxatriangulenium tetrafluoroborate (**5**)

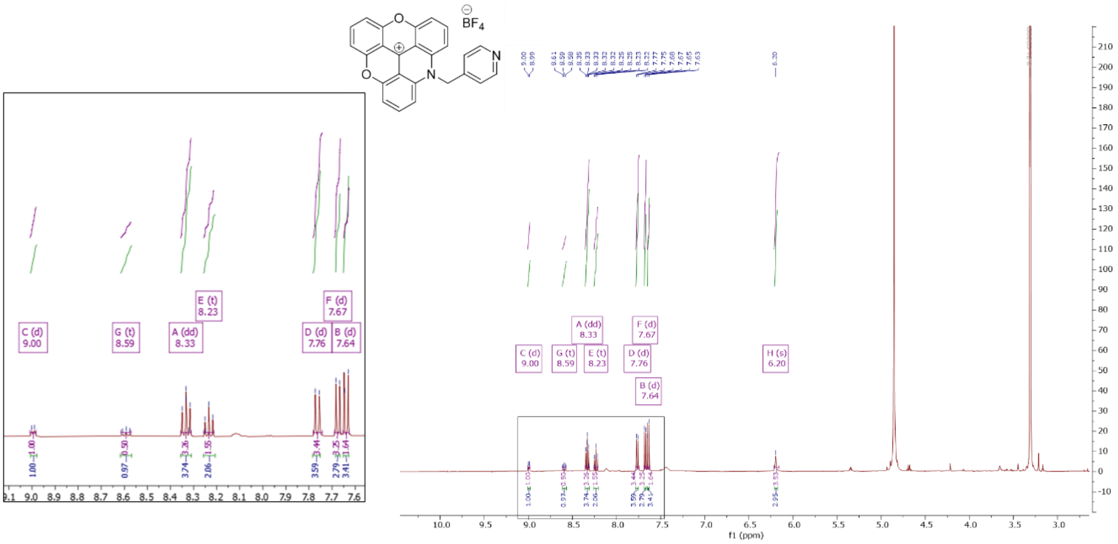

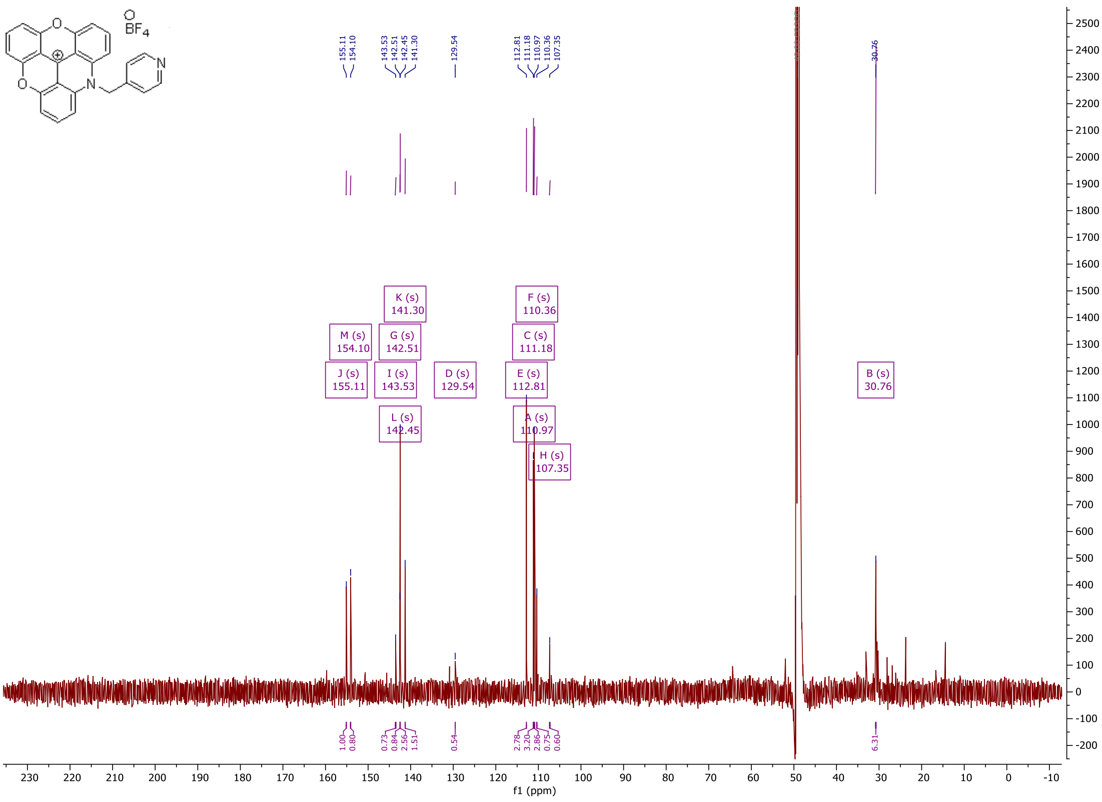

4-(pyridin-2-ylmethyl)- 4-aza-8,12-dioxatriangulenium tetrafluoroborate (**6**)

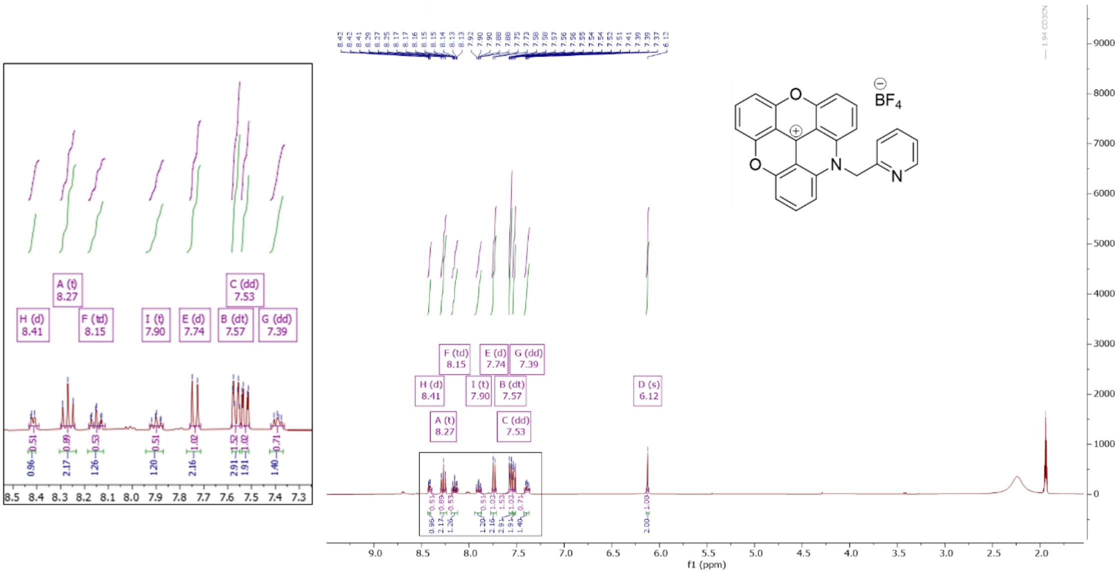

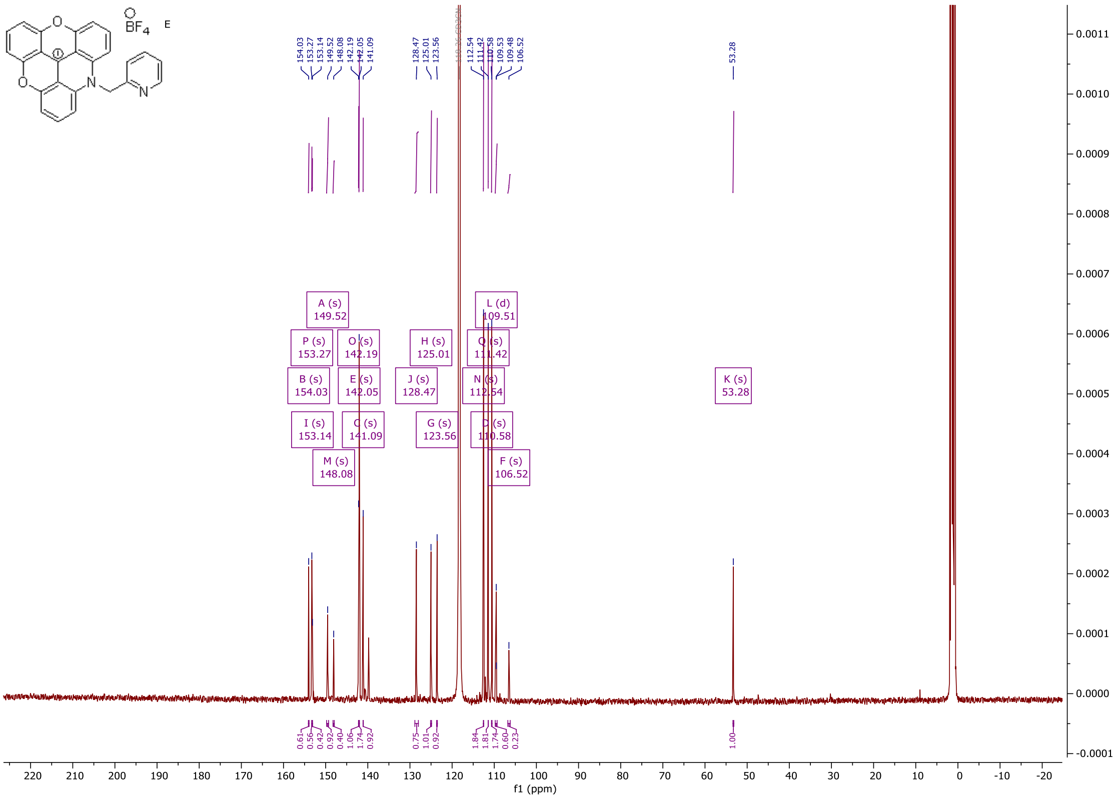

NMR of DAOTA compounds (**7**-**8**)

8-amino-4-(3-cyanophenyl)-4,8-diaza-12-oxatriangulenium tetrafluoroborate (**7**)

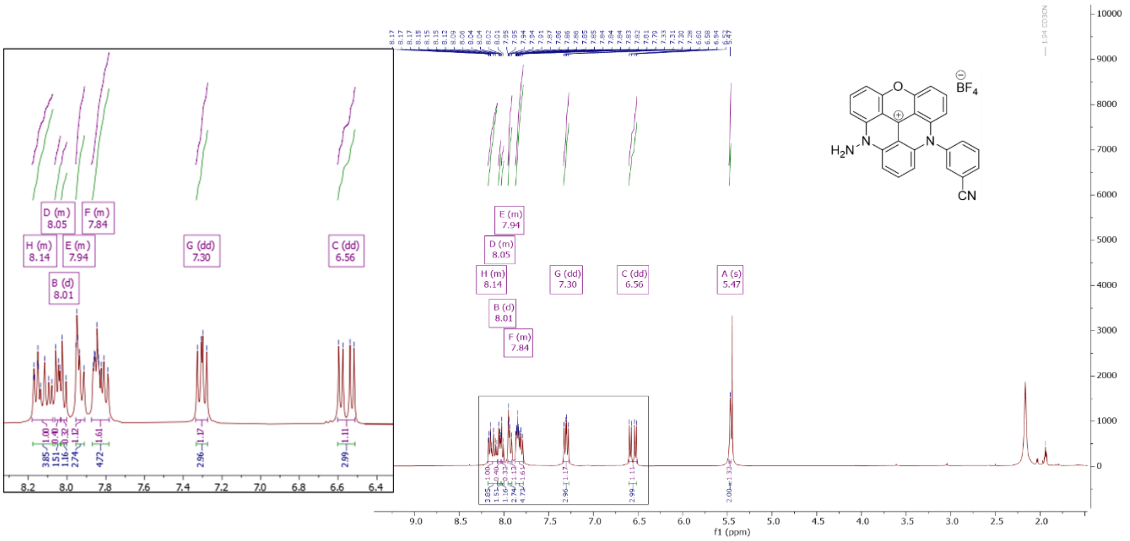

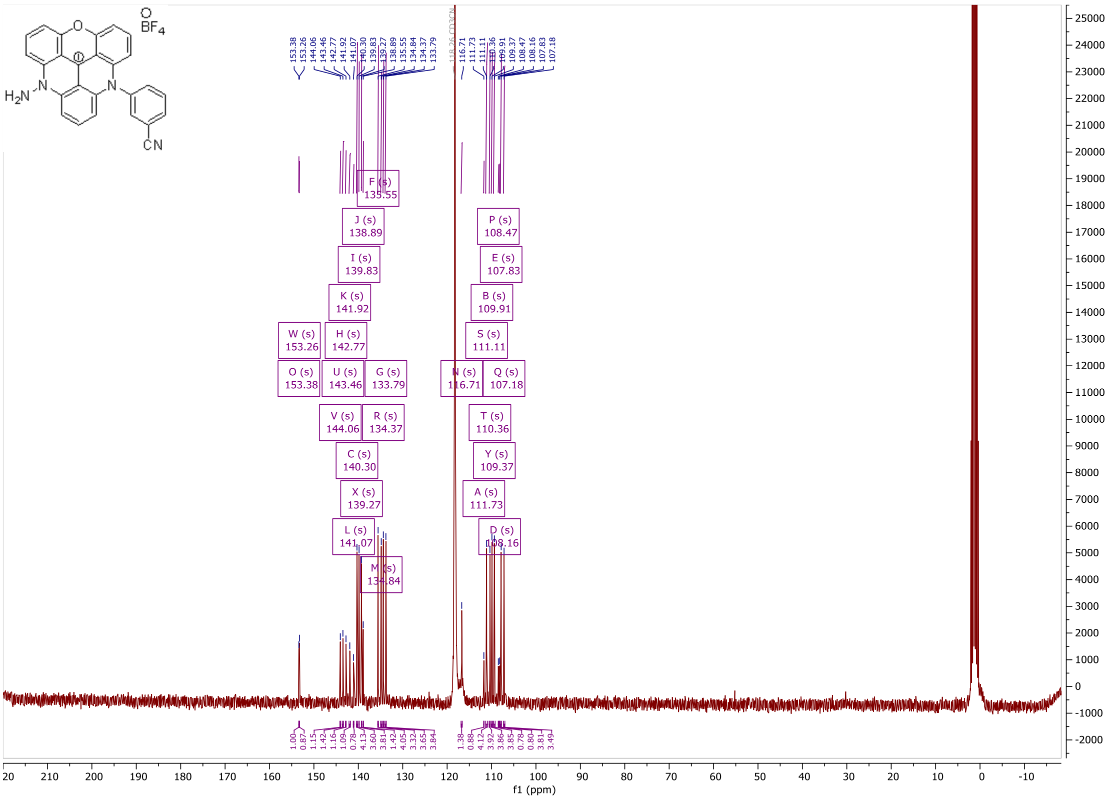

8-(2-aminoethyl)-4-(3-cyanophenyl)-4,8-diaza-12-oxatriangulenium tetrafluoroborate (**8**)

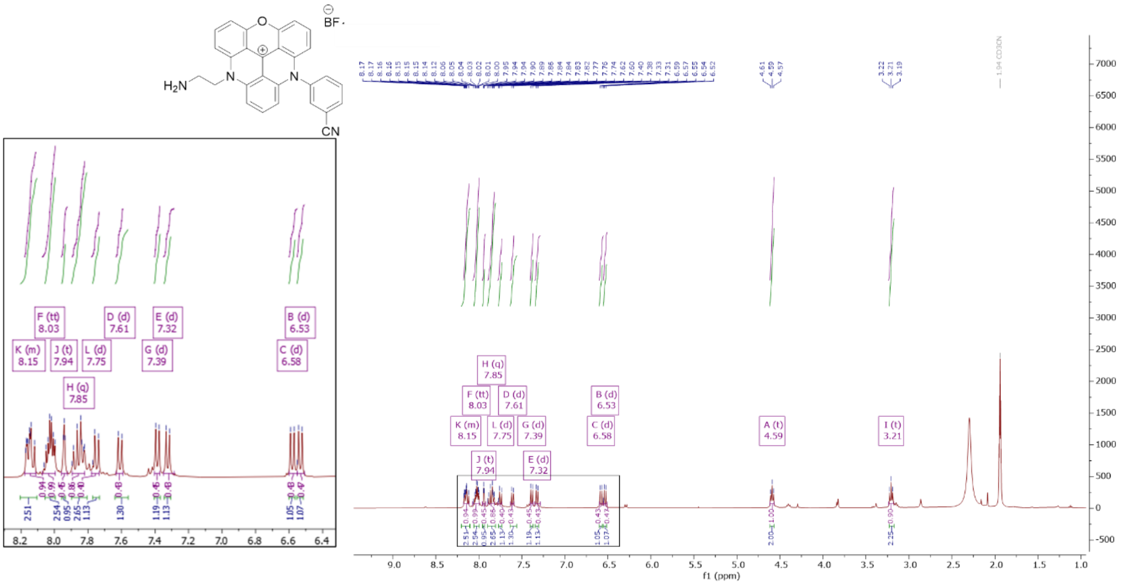

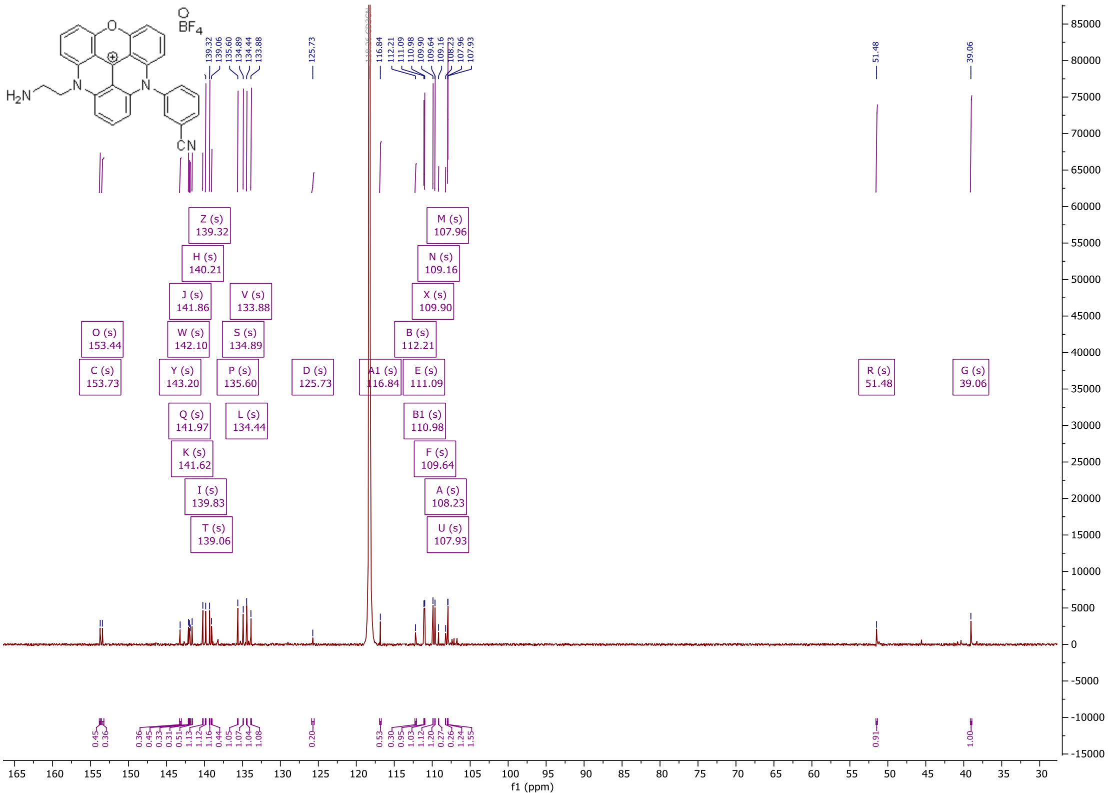

References

1. Shivalingam, A.; Izquierdo, M. A.; Marois, A. L.; Vyšniauskas, A.; Suhling, K.; Kuimova, M. K.; Vilar, R., The interactions between a small molecule and G-quadruplexes are visualized by fluorescence lifetime imaging microscopy. *Nat. Commun.* **2015,** *6*, 8178. DOI: 10.1038/ncomms9178.
2. Rosenberg, M.; Junker, A. K. R.; Sørensen, T. J.; Laursen, B. W., Fluorescence pH Probes Based on Photoinduced Electron Transfer Quenching of Long Fluorescence Lifetime Triangulenium Dyes. *ChemPhotoChem* **2019,** *3*, 233-242. DOI: 10.1002/cptc.201800266.
3. González-Vera, J. A.; Lv, F.; Escudero, D.; Orte, A.; Guo, X.; Hao, E.; Talavera-Rodriguez, E. M.; Jiao, L.; Boens, N.; Ruedas-Rama, M. J., Unusual spectroscopic and photophysical properties of solvatochromic BODIPY analogues of Prodan. *Dyes and Pigments* **2020**, 108510. DOI: 10.1016/j.dyepig.2020.108510.
4. Molinspiration miLogP 2.2. https://www.molinspiration.com/services/logp.html (accessed August 2025).
5. Daina, A.; Michielin, O.; Zoete, V., iLOGP: A Simple, Robust, and Efficient Description of n-Octanol/Water Partition Coefficient for Drug Design Using the GB/SA Approach. *J. Chem. Inf. Model.* **2014,** *54*, 3284-3301. DOI: 10.1021/ci500467k.
6. Daina, A.; Michielin, O.; Zoete, V., SwissADME: a free web tool to evaluate pharmacokinetics, drug-likeness and medicinal chemistry friendliness of small molecules. *Sci. Rep.* **2017,** *7*, 42717. DOI: 10.1038/srep42717.
7. Jeong, S.; Widengren, J.; Lee, J.-C., Fluorescent Probes for STED Optical Nanoscopy. *Nanomaterials* **2022,** *12*, 21. DOI.
8. Schindelin, J.; Arganda-Carreras, I.; Frise, E.; Kaynig, V.; Longair, M.; Pietzsch, T.; Preibisch, S.; Rueden, C.; Saalfeld, S.; Schmid, B.; Tinevez, J.-Y.; White, D. J.; Hartenstein, V.; Eliceiri, K.; Tomancak, P.; Cardona, A., Fiji: an open-source platform for biological-image analysis. *Nat. Meth.* **2012,** *9*, 676-682. DOI: 10.1038/nmeth.2019.
9. Jiao, L.; Yu, C.; Wang, J.; Briggs, E. A.; Besley, N. A.; Robinson, D.; Ruedas-Rama, M. J.; Orte, A.; Crovetto, L.; Talavera, E. M.; Alvarez-Pez, J. M.; Van der Auweraer, M.; Boens, N., Unusual spectroscopic and photophysical properties of meso-tert-butylBODIPY in comparison to related alkylated BODIPY dyes. *RSC Adv.* **2015,** *5*, 89375-89388. DOI: 10.1039/C5RA17419H.
10. Horobin, R. W., Predicting Mitochondrial Targeting by Small Molecule Xenobiotics Within Living Cells Using QSAR Models. In *Mitochondrial Medicine: Volume II, Manipulating Mitochondrial Function*, Weissig, V.; Edeas, M., Eds. Springer New York: New York, NY, 2015; pp 13-23.
11. Horobin, R.; Stockert, J.; Rashid-Doubell, F., Uptake and localization mechanisms of fluorescent and colored lipid probes. Part 3. Protocols for predicting intracellular localization of lipid probes using QSAR models. *Biotechnic & Histochemistry* **2015,** *90*, 255-263. DOI: 10.3109/10520295.2015.1006680.
12. Horobin, R. W., Where do dyes go inside living cells? Predicting uptake, intracellular localisation, and accumulation using QSAR models. *Color. Technol.* **2014,** *130*, 155-173. DOI: doi:10.1111/cote.12093.
13. Belmonte-Reche, E.; Martínez-García, M.; Guédin, A.; Zuffo, M.; Arévalo-Ruiz, M.; Doria, F.; Campos-Salinas, J.; Maynadier, M.; López-Rubio, J. J.; Freccero, M.; Mergny, J.-L.; Pérez-Victoria, J. M.; Morales, J. C., G-Quadruplex Identification in the Genome of Protozoan Parasites Points to Naphthalene Diimide Ligands as New Antiparasitic Agents. *J. Med. Chem.* **2018,** *61*, 1231-1240. DOI: 10.1021/acs.jmedchem.7b01672.
